# Rapid formation and real-time observation of micron-sized conjugated nanofibers with tunable lengths and widths in 20 minutes by living crystallization-driven self-assembly†

**DOI:** 10.1039/d0sc02891f

**Published:** 2020-07-29

**Authors:** Sanghee Yang, Tae-Lim Choi

**Affiliations:** Department of Chemistry, Seoul National University Seoul 08826 Korea tlc@snu.ac.kr

## Abstract

Preparing well-defined semiconducting nanostructures from conjugated polymers is of paramount interest for organic optoelectronic devices. Several studies have demonstrated excellent structural and size control from block copolymers (**BCPs**) containing non-conjugated blocks *via* crystallization-driven self-assembly (CDSA); however, the precise control of their size and shape remains a challenge due to their poor solubility, causing rapid and uncontrolled aggregation. This study presents a new type of fully conjugated BCP comprising two polyacetylene derivatives termed poly(cyclopentenylene-vinylene) to prepare semiconducting 1D nanofibers. Interestingly, the widths of nanofibers were tuned from 12 to 32 nm based on the contour lengths of their crystalline core blocks. Their lengths could also be controlled from 48 nm to 4.7 μm using the living CDSA. Monitoring of the growth kinetics of the living CDSA revealed the formation of micron-sized 1D nanofibers in less than 20 min. The rapid CDSA enabled us to watch real-time growth using confocal fluorescence microscopy.

## Abstract 2

New fully conjugated block copolymers formed semiconducting 1D nanofibers with excellent structural and size control. The rapid living CDSA enabled us to watch the real-time video of the whole self-assembly process.

## Graphical abstract

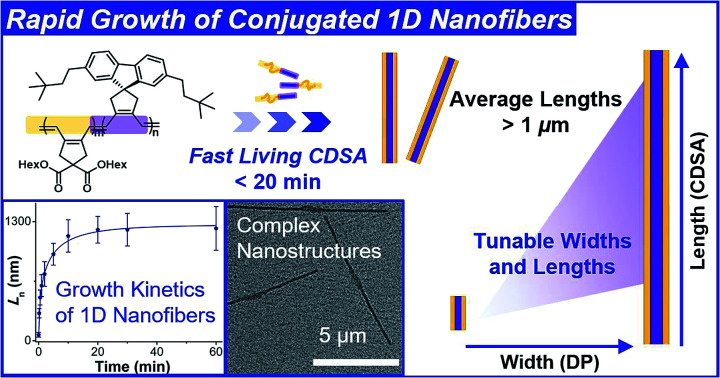

## Introduction

Conductive molecules are highly advantageous for sensors, functional coatings, and electronic devices.^1–4^ Among them, conjugated polymers have gained enormous attention due to their advantageous physical properties including their low weight and flexibility.^5–7^ For example, poly(3-hexylthiophene) (P3HT),^8–10^ poly(*para*-phenylenevinylene) (PPV),^11^ and polyfluorene (PF)^12^ have been widely used in various device applications. Notably, several reports have highlighted the important relationship between the performance of electronic materials and their nanostructures.^13–17^ Therefore, constructing nanostructures by using polymer self-assembly to enable precise control on size and shape has become important for device applications.^16,17^

There have been numerous studies on the control of polymeric nanostructures over decades. Many uniform nanostructures have been created using various amphiphilic block copolymers (**BCPs**) with differing solubilities.^18–20^ More recently, pioneered by Ian Manners group, an ingenious method termed Crystallization-Driven Self-Assembly (CDSA) was developed. The CDSA method enables the control of the nanostructure with excellent precision.^21–31^ Many **BCPs** containing semicrystalline polymers such as polyferrocenylsilane (PFS),^21–23^ poly(ε-caprolactone) (PCL),^24,25^ polyethylene (PE),^26,27^ and polylactide (PLA)^28,29^ have been successful in forming various uniform nanostructures from 0D-micelle to 3D-supermicelles *via* CDSA. Despite excellent structural control, the CDSA method has one drawback; it generally takes several hours to days for complete assembly. To broaden the utility of these nanostructures, conjugated oligomers and polymers have been used to form uniform nanostructures *via* this CDSA method.^30–34^ However, this strategy may be complicated and challenging due to the strong π–π interaction among conjugated polymers. This reduces their solubility leading to easy aggregation, and disrupting controlled self-assembly. Whilst this issue may be resolved by synthesizing **BCPs** containing highly soluble non-conjugating shells,^33,34^ this insulating block would inevitably limit the potential of the resulting partially semiconducting nanostructures as electronic materials.

**Scheme 1:**
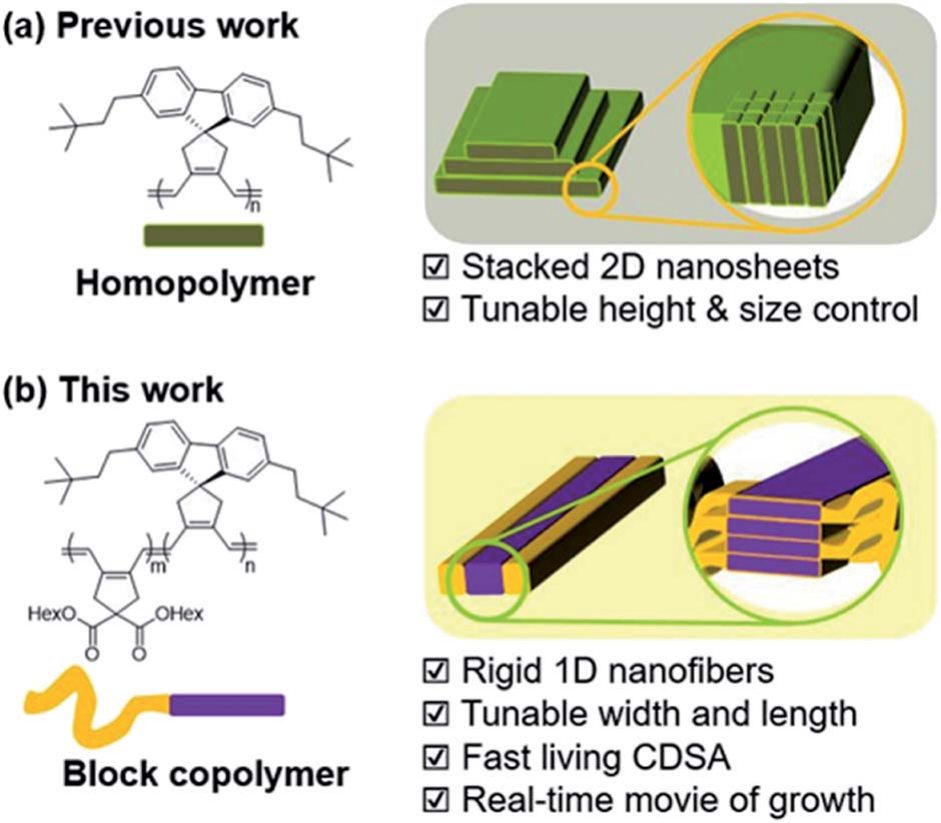
New strategy to prepare 1D nanofibers with tunable widths and lengths *via* rapid self-assembly.

To accelerate the self-assembly process and produce nanostructures more efficiently, a one-pot technique named Polymerization-Induced CDSA (PI-CDSA) was developed where CDSA successful occurred during or after the polymerization.^35–37^ For the spontaneous formation of the conjugated nanoparticles, we have developed another strategy termed *In situ* Nanoparticlization of Conjugated Polymers (INCP).^38–42^ INCP is a process whereby insoluble conjugated polymers are intentionally introduced as the second block. During the synthesis of **BCPs**, the strong π–π interaction induces spontaneous nanoparticlization, producing semiconducting nanostructures without post-treatment.^38–40^ A recent study reported large 2D structures from crystalline poly(cyclopentenylene-vinylene) (PCPV) consisting of fluorene and bulky side chains such as neohexyl and silyl groups. Interestingly, the height of the individual 2D sheets was determined by the degree of polymerization (DP) of these homopolymers as their rigid PCPV backbones were self-assembled side-by-side without chain-folding (Scheme 1a).^41,42^

Despite the lack of precise control over the nanostructures, this crystalline PCPV showed potential and its expansion to BCP microstructures may provide insights on achieving higher control of the nanostructures. Herein, we report the formation of well-defined semiconducting 1D nanofibers from **BCPs** having the PCPV as the core block and another PCPV as a soluble shell block.^43^ The width of the nanofibers was precisely controlled by the DP of the core block due to the living cyclopolymerization, and the length was controlled *via* the living CDSA. These two living processes led to not only narrow dispersity of width and length, but also the successful formation of block comicelles (Scheme 1b). Interestingly, this CDSA occurred rapidly, taking 10 min to reach micron-sized lengths, thereby allowing direct visualization of this self-assembly growth using confocal laser optical microscopy.

## Results and discussion

To prepare uniform nanostructures, we synthesized fully conjugated **BCPs** consisting of the first PCPV block with soluble dihexyl side-chains (**M1**) and the crystalline second block from **M2**. To minimize dispersities, various **BCPs** (with the fixed [**M1**]/[**I**] ratio of 50) were polymerized in tetrahydrofuran (THF) at 0 °C using the third-generation Grubbs catalyst (**G3**).^44^ After the completion of polymerizations, the reactions were quenched by excess ethyl vinyl ether and the polymers were isolated by precipitation in methanol at 25 °C. Six **P150**-*b*-**P2ns** were prepared with [**M2**]/[**I**] ratios from 10 to 66 in excellent isolated yields (Table 1).

**Table 1:** Living cyclopolymerization to prepare various **BCPs**

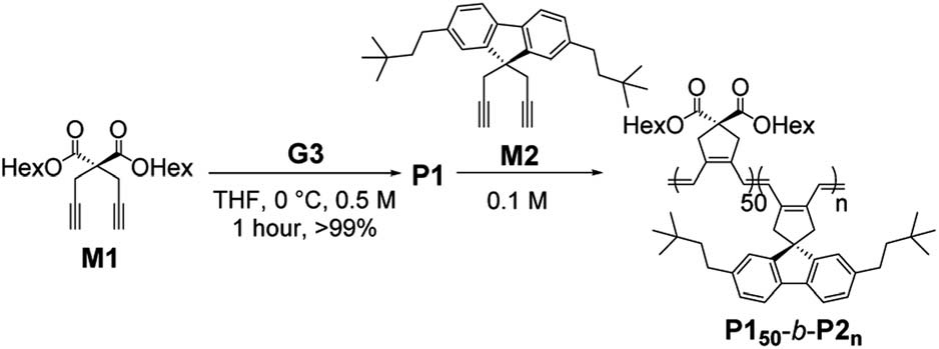						
Entry	[**M1**] : [**M2**] : [cat]	Time	Conv.^a^ (**M2**)	Yield (%)	*M*_n_ b ^,^ c (kDa)	*Đ* b ^,^ c
1	50 : 10 : 1	2.5 h	>99%	91	23.3^b^	1.15^b^
2	50 : 22 : 1	4 h	>99%	98	38.6^b^	1.10^b^
3	50 : 33 : 1	6 h	>99%	90	47.4^b^	1.13^b^
4	50 : 44 : 1	8 h	>99%	95	10 440^c^	1.56^c^
5	50 : 55 : 1	9 h	>99%	92	70 350^c^	1.26^c^
6	50 : 66 : 1	11 h	>99%	92	115 800^c^	1.13^c^

a Calculated by ^1^H NMR analysis in CDCl_3_ before precipitation.

b Determined by chloroform SEC, calibrated using polystyrene standards.

c Determined by AF4 fractograms in chloroform using 0.205 as a d*n*/d*c* value.

**Fig. 1:**
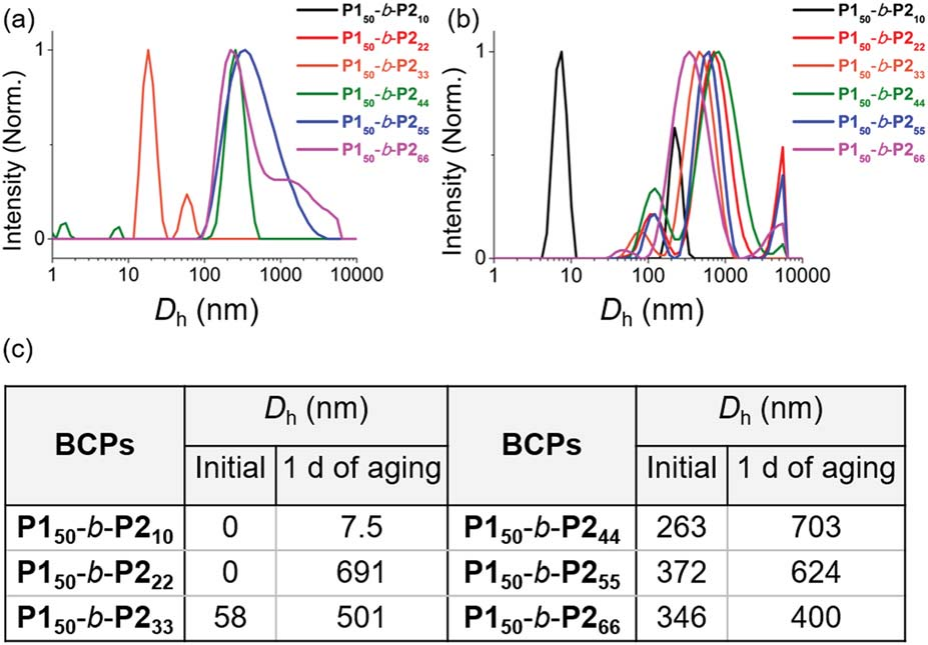
DLS profiles of **BCPs** solutions (1 g L^−1^ chloroform) (a) without aging and (b) after 1 day of aging at 25 °C. (c) A table of *D*_h_ values of the DLS profiles in (a) and (b).

We characterized the purified **BCPs** by ^1^H nuclear magnetic resonance (NMR) spectroscopy to get some clues about spontaneous self-assembly. For **P150**-*b*-**P210** and **P150**-*b*-**P222**, signals from both blocks were observed with expected integrations from the feed ratios, indicating low degrees of aggregation in chloroform. However, as the DP of **P2** increased to 33, signals for the **P2** block were only 28% of that expected from the feed ratio. The integration values further decreased, reaching a minimum of 11% for **P150**-*b*-**P266** despite the full conversion of **M2** (Fig. S1 and S2†).^41^ This phenomenon agrees well with the previous investigation supporting for the INCP mechanism where longer **BCPs** spontaneously formed more crystalline cores which then, were not detectable in ^1^H NMR analysis.^38–40,45^ To better characterize the **BCPs** by NMR, we attempted various deuterated solvents such as benzene, 1,4-dioxane, chlorobenzene, and *o*-dichlorobenzene to dissolve both blocks, but still chloroform was the best solvent for the **BCPs** (Fig. S3 and S4†). Fortunately, with ^1^H NMR analysis at 47 °C in chloroform, more quantitative analysis was possible for **P150**-*b*-**P233** and **P150**-*b*-**P244** due to better solubility of **P2** at the higher temperature (Fig. S5†). More definitive support for INCP was provided by dynamic light scattering (DLS) analysis which gives hydrodynamic diameters (*D*_h_, these values should be treated as qualitative estimation). When all **BCPs** were dissolved in 1 g L^−1^ chloroform, large *D*_h_ from 58 nm for **P150**-*b*-**P233** to 346 nm for **P150**-*b*-**P266** were observed in accordance with the ^1^H NMR analysis, and these *D*_h_ values were retained even at 0.0001 g L^−1^ (Fig. 1a and S6†). The direct indication of successful INCP using **P150**-*b*-**P266** was obtained from the TEM imaging, DLS, and UV-Vis analysis of the *in situ* sample from the reaction solution (Fig. S7†). Due to the INCP, we could only measure the molecular weight (*M*_n_) of smaller **BCPs** (for **P150**-*b*-**P210**: 23.3 kDa, **P150**-*b*-**P222**: 38.6 kDa, and **P150**-*b*-**P233**: 47.4 kDa) by chloroform size-exclusion chromatography (SEC). This linear increase in *M*_n_s and dispersities (*Đ*) lower than 1.15 supported successful living cyclopolymerization. Fortunately, the *M*_n_s of strongly aggregated larger **BCPs** could be estimated using an advanced technique known as asymmetric flow field-flow fractionation (AF4) analysis to determine *M*_n_ up to 115 MDa, supporting *in situ* self-assembly (Table 1 entries 4 to 6, and Fig. S8†).^46,47^

To further promote self-assembly, we aged BCP solutions (1 g L^−1^ chloroform) at 25 °C for 1 d, and found an overall increase in *D*_h_, up to 700 nm under identical conditions, except for **P150**-*b*-**P210** (Fig. 1b and c).^48^ The **BCP** having the shortest core block of **P210** was still in an unimeric state due to its low crystallinity. A further decrease in the signals for the core block **P2***via* the ^1^H NMR spectra was also observed. The integration values for **P2** in **P150**-*b*-**P222** decreased from 100 to 74% and, for **P150**-*b*-**P244–66**, these signals were barely observable, indicating quantitative self-assembly in the absence of unimers (Fig. S9†). The UV-Vis analysis of these conjugated **BCPs** showed much stronger vibronic peaks at 595 nm indicating the formation of more ordered structures (Fig. S10–S12†). However, ^1^H NMR and DLS analysis showed that the initial **BCPs** in 1 g L^−1^ dichloromethane (DCM) solution were already undergoing self-assembly even without aging. This more facile and rapid self-assembly may be due to the lower solubility of **P2** in DCM than in chloroform leading to more rapid crystallization (Fig. S13†).

**Fig. 2:**
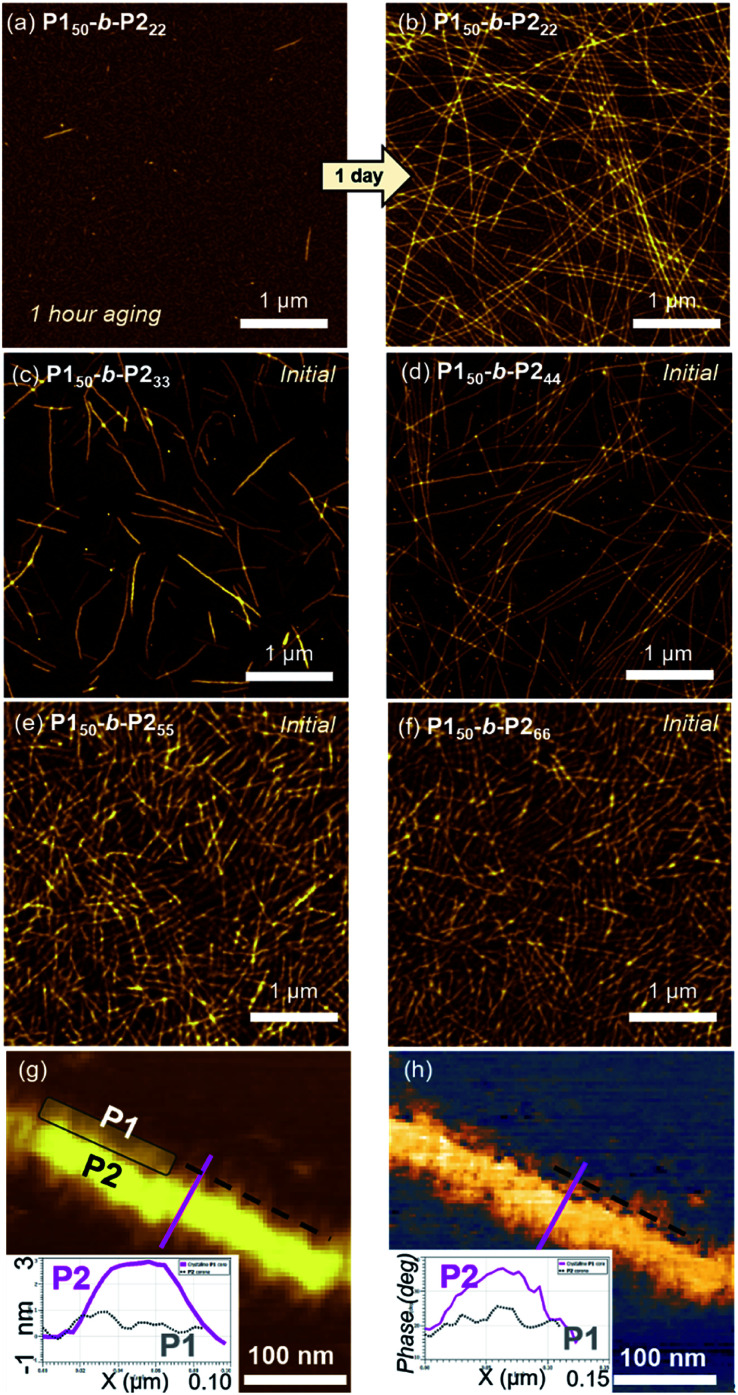
AFM images obtained from 1 g L^−1^ chloroform solutions of (a) **P150**-*b*-**P222** after 1 h, and (b) after 1 d aging at 25 °C, (c) **P150**-*b*-**P233**, (d) **P150**-*b*-**P244**, (e) **P150**-*b*-**P255**, and (f) **P150**-*b*-**P266** without aging. The higher magnification of (g) height, and (h) phase images of the 1D nanofibers from **P150**-*b*-**P255**.

Atomic force microscopy (AFM) imaging without aging was undertaken to visualize these structures. We observed spontaneous formation of 1D nanofibers of **BCPs***via* INCP, with the exception of **P150**-*b*-**P222** which required an aging time of 1 h or longer (Fig. 2a–f and S14†). After aging, the length of the 1D nanofibers from **P150**-*b*-**P222** grew to a maximum of greater than 20 μm, with no branching (Fig. 2b and S15†). Although their heights ranged from 3.6 to 5.5 nm without a certain trend independent of the DP of **P2**, their widths were observed to roughly increase in proportion to the DP of **P2** (Fig. S16†). Even with dilution from 1 to 0.05 g L^−1^, this width trend continued despite the reduction of their lengths to approximately 1 μm (Fig. S17†). With high magnification AFM images of height and phase modes, the core could be distinguished from the shell to show that the crystalline **P2** block was taller and denser than the outer **P1** block (Fig. 2g and h).

**Fig. 3:**
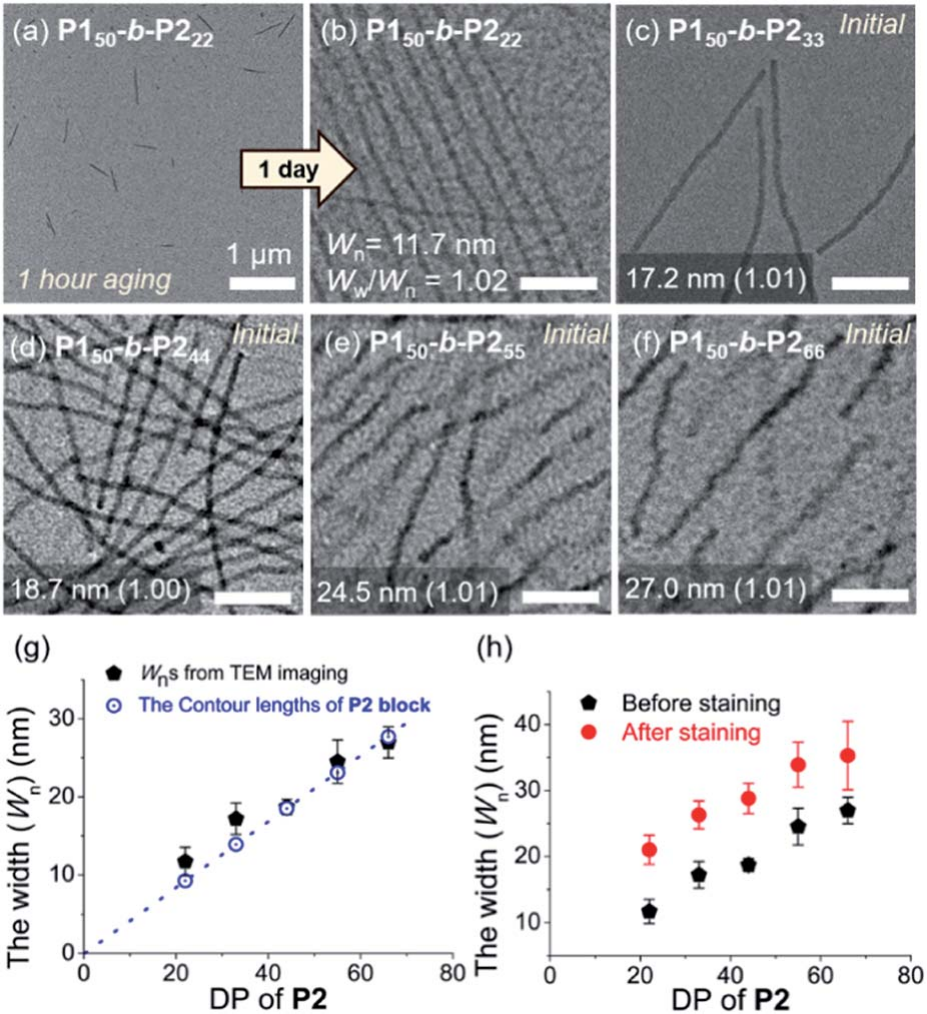
TEM images obtained from 1 g L^−1^ chloroform solutions of (a) **P150**-*b*-**P222** after 1 h, and (b) after 1 d aging at 25 °C, (c) **P150**-*b*-**P233**, (d) **P150**-*b*-**P244**, (e) **P150**-*b*-**P255**, and (f) **P150**-*b*-**P266** without aging (scale bar for (b)–(f), 200 nm). Plots of the DP of **P2***versus* (g) average width (*W*_n_) of the core of the 1D nanofibers compared to the theoretical length of the fully stretched **P2** block (a dotted line, Fig. S22†), and (h) *W*_n_ after staining with RuO_4_ vapour.

To attain more precise information on width, transmission electron microscopy (TEM) imaging was used. TEM images showed the long fibers with high rigidity without aging, with the exception of **P150**-*b*-**P222** (Fig. 3a–f and S18†). Without staining, vivid visualization of the electron dense crystalline **P2** core was possible, and their measured widths showed a linear increase from 12 to 27 nm, according to an increase of the DP of **P2** from 22 to 66 with width dispersities (*W*_w_/*W*_n_) less than 1.02 (Fig. S18c and S19–S21†). In contrast to a previous study on self-assembly of homopolymer of **P2**, where the DP of the polymer matched well with the height of 2D nanosheets (Scheme 1a), in this study, the width of the 1D nanofibers was well matched to the theoretically estimated contour length of the **P2** block (Fig. 3g, see S22 and Table S1† for calculation of the contour lengths by MM2 computational method). Staining using RuO_4_ vapour also enabled the detection of the flexible **P1** block, and measurement of the full width of the 1D nanofibers including the shell demonstrated the presence of thicker fibers from 21 to 35 nm (Fig. 3h and S23†).^49^

**Fig. 4:**
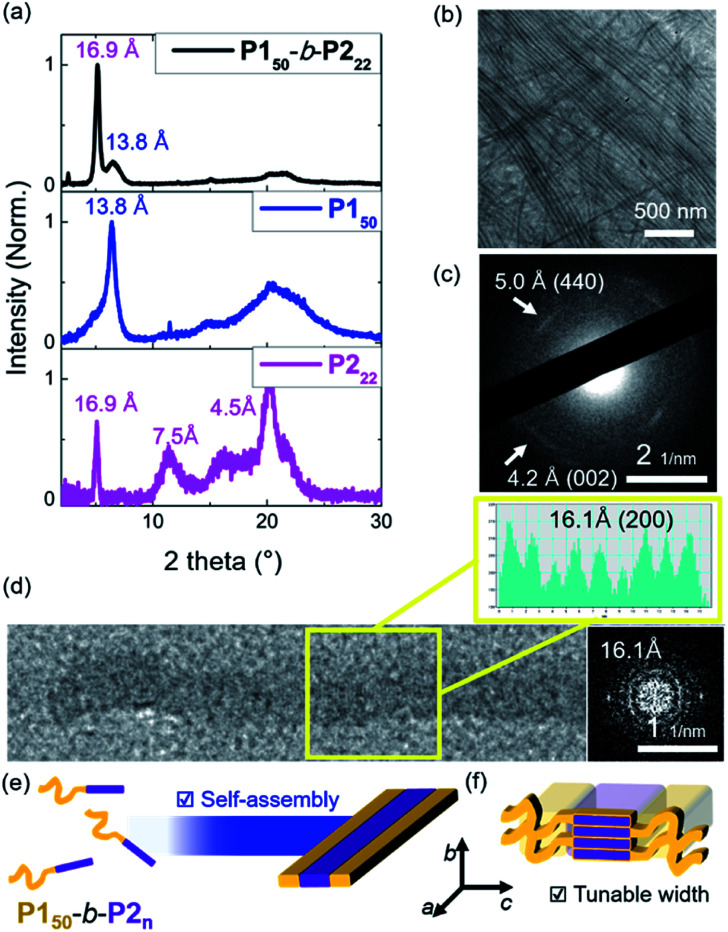
(a) Film XRD plots of the 1D nanofibers from the 10 g L^−1^ chloroform solution of the **P150**-*b*-**P222**, homopolymers of the **P150** and **P222**. (b) HR-TEM image of the nanofiber bundles and (c) its SAED image showing *d*-spacings at 5.0 Å and 4.2 Å. (d) A HR-TEM image of a single 1D nanofiber with an additional cross-sectional histogram, and its diffraction pattern showing *d*-spacing of 16.1 Å. Schematic showing (e) self-assembly of 1D nanofibers, and (f) the detailed orthorhombic crystal array of the core **P2** block.

To investigate the crystallinity of the **BCPs** and their 1D nanofibers in detail, film X-ray diffraction (FXRD) analysis was conducted on **P150**-*b*-**P222**. A sharp peak was observed at a *d*-spacing of 16.9 Å originating from **P2**, a much weaker peak at *d*-spacing of 13.8 Å was produced from **P1**, and broad peaks between 4 Å and 8 Å were observed (Fig. 4a and S24†). The **P1** signal disappeared in the aged sample of **P150**-*b*-**P222** and other samples of **BCPs** having longer **P2** block. This indicates that the crystallinity of the **P2** block dominated the formation of the 1D nanofibers, and the **P1** block formed mostly amorphous structure in the self-assembled nanofibers.^41^ We also directly observed the crystalline array of the 1D nanofibers from the electron diffraction patterns by selected area electron diffraction (SAED) analysis and fast Fourier transform (FFT) analysis of the high-resolution TEM (HR-TEM) images (Fig. 4b–d and S25†). The SAED showed one *d*-spacing at 5.0 Å along the longitudinal direction of the 1D nanofibers and another at 4.2 Å in the orthogonal direction. Additionally, spots at 16.1 Å along the longitudinal direction of the 1D nanofibers were obtained by FFT analysis of the HR-TEM. Based on these diffraction patterns and previous findings on the orthorhombic crystal lattice of the **P2** homopolymer, we assigned 16.1 Å as a *d*-spacing in the (200) plane and 4.2 Å in the (002) plane. The fact that the **P2** block forms the core by lying down on the *c* axis, is consistent with the average widths of the core matching the contour lengths of **P2** (Fig. 3g and 4e, f).^43^

As the width of the 1D nanofibers could be precisely controlled, we attempted to control their lengths by using the living CDSA; epitaxial growth from the uniform seed through the addition of unimers. Fortunately, after sonicating 0.1 g L^−1^ chloroform solution of long nanofibers from **P150**-*b*-**P222** for 30 s using an ultrasonicator (11.8 W cm^−2^) at 0 °C, we obtained a seed solution with an average length (*L*_n_) of 60.3 nm and a relatively narrow length distribution (*L*_w_/*L*_n_ = 1.18, characterized by TEM) (Fig. S26†). Then, 10 g L^−1^ chloroform solution of unimers (before aging), was added to the seed solution at unimer-to-seed (U/S) ratios from 1 to 10. After 1 h of aging at 25 °C, the *D*_h_ from DLS analysis increased gradually and more definitively, *L*_n_ from TEM imaging increased linearly according to the U/S ratio. However, the 1D nanofibers continued to elongate by doubling in length after 5 h (Fig. S27, S28a and b†). Even with U/S ratios of 1, or more definitively, even without added unimers, the nanofibers became longer than 10 μm after 1 d of aging (Fig. S28c and d†). These results indicated that fiber-to-fiber assembly occurred between their sticky ends even after the completion of the initial seed-to-unimer assembly (or seeded growth), thereby resulting in undesirable end-to-end coupling in chloroform.^50–52^ Presumably, the adhesive unimers could be generated by the dynamic exchange between the nanofiber and unimer to promote the end-to-end assembly.^53^ To date, end-to-end coupling was reported to be much slower than seeded growth,^50^ but in this case, it occurred readily, even at −20 °C (Fig. S28e and f†).

**Fig. 5:**
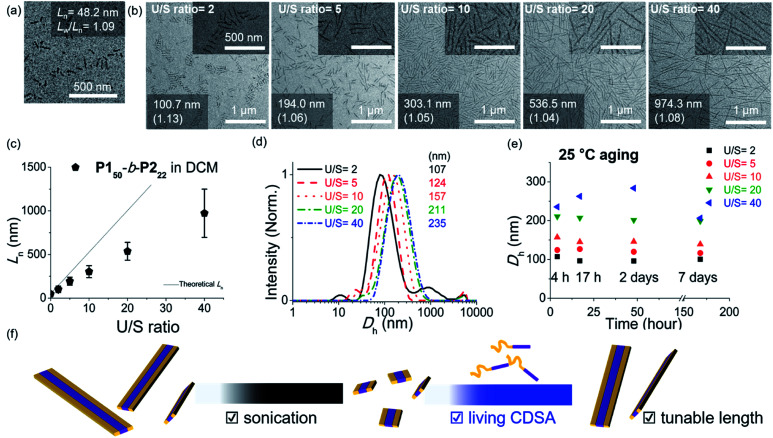
TEM images of (a) the seed-micelle of 1D nanofibers after optimized sonication and (b) length controlled 1D nanofibers showing an increase in their lengths with increasing U/S ratios (scale bar of inset images, 500 nm). The numbers in images indicated “the average *L*_n_ and its length dispersity”. (c) The plot of U/S ratios *versus* the *L*_n_s. (d) *D*_h_ values of the length-controlled 1D nanofibers. (e) The plot of time (h) *versus* the *D*_h_ values. (f) Schematic of the living CDSA of 1D nanofibers *via* the seeded growth mechanism.

Alternatively, we prepared a seed-solution of **P150**-*b*-**P222** in 0.1 g L^−1^ DCM by sonicating for 2 min (*L*_n_ of 48.2 nm, *D*_h_ of 62.1 nm, and *L*_w_/*L*_n_ = 1.13), to achieve lower solubility of the seeds in DCM in order to prevent the formation of sticky ends and thereby suppress end-to-end coupling (Fig. 5a). Subsequently, the same unimer solution of **P150**-*b*-**P222** at 10 g L^−1^ in chloroform was added at various temperatures with U/S ratios 5. This resulted in successful seeded growth at 10 °C to form 1D nanofibers (*L*_n_ = 188.0 nm, *L*_w_/*L*_n_ = 1.11), whose length remained uniform even after 13 h at 10 °C (Fig. S29†). We attempted this for the living CDSA under the same conditions, at various U/S ratios of 2, 5, 10, 20, and 40. Following 4 h of aging, their *L*_n_ values linearly increased from 100.7 to 972.3 nm according to the U/S ratios (Fig. 5b and c). The low-magnified TEM images show that the length dispersity was less than 1.15 indicating successful living CDSA. Notably, measured *L*_n_ seems to be substantially shorter than the theoretical lengths predicted based on the U/S ratio. This may be due to competitive homogeneous nucleation occurring simultaneously with the seeded-growth, because the solubility of the **P2** block became slightly lower in DCM.^54,55^ The living CDSA was also qualitatively supported by DLS analysis, where their *D*_h_ values gradually increased with higher U/S ratios (Fig. 5d). These *D*_h_ values also remained fairly constant during aging for a week at both 10 and 25 °C, showing high stability in solution without end-to end coupling (Fig. 5e and S30†).

**Fig. 6:**
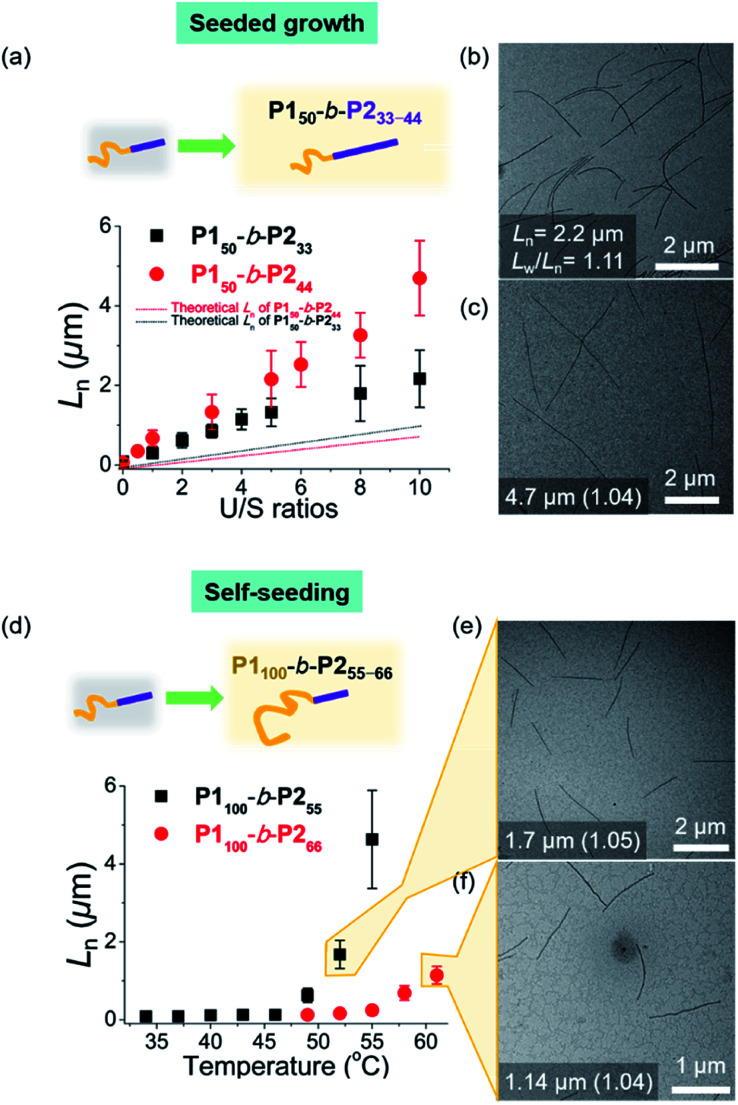
Living CDSA for larger **BCPs**. For **P150**-*b*-**P233–44**, (a) plots of U/S ratios *versus L*_n_ showing successful seeded growth, and the solid lines represented theoretical *L*_n_ values. TEM images of U/S ratios = 10 for (b) **P150**-*b*-**P233**, and (c) **P150**-*b*-**P244**. For **P1100**-*b*-**P255–66**, (d) plots of annealing temperature (°C) *versus L*_n_ showing CDSA through self-seeding. TEM images of the micron-sized 1D nanofibers from (e) **P1100**-*b*-**P255**, and (f) **P1100**-*b*-**P266***via* self-seeding. The number in images indicates “the average *L*_n_ and its length dispersity”.

To expand the scope of the living CDSA to wider nanofibers, we attempted the living CDSA from larger **BCPs**. This was challenging as the larger **BCPs** underwent INCP during synthesis due to lower solubility, particularly in DCM. The same sonication protocol successfully produced the seed micelle (**P150**-*b*-**P233**: *L*_n_ = 84.6 nm, *L*_w_/*L*_n_ = 1.13, and **P150**-*b*-**P244**: *L*_n_ = 66.1 nm, *L*_w_/*L*_n_ = 1.13) by switching back to 0.1 g L^−1^ chloroform instead of DCM. Furthermore, we were able to obtain the corresponding unimer solutions in 0.1 g L^−1^ chloroform by heating at 60 °C (Fig. S31†). These unimers were then added to the seeds with various U/S ratios from 1 to 10. After 1 h of aging at RT, their *L*_n_ increased linearly up to 2.2 μm for **P150**-*b*-**P233**, and 4.7 μm for **P150**-*b*-**P244** according to the U/S ratios while length dispersity (*L*_w_/*L*_n_) remained below 1.15, supporting the living CDSA by seeded growth (Fig. 6a–c, S32 and S33†). In both cases, a rise in temperature to prepare the unimer solutions might dissolve some seeds as a result of the improved solubility of the **BCPs**, resulting in the longer 1D nanofibers than the theoretically predicted length.^54^ Also, *L*_n_s in both cases remained after aging for 1 d suggesting that end-to-end coupling did not occur in chloroform (Fig. S34†).

To achieve seeded growth of even wider 1D nanofibers, we further heated **P150**-*b*-**P255–66** up to 80 °C but failed to obtain a unimer solution due to even lower solubility (Fig. S31†). To improve solubility, we prepared new **P1100**-*b*-**P255** and **P1100**-*b*-**P266** with a longer shell block (with [**M1**]/[**I**] = 100). Full characterizations using ^1^H NMR, AF4, DLS, TEM, and AFM analyses indicated similar behaviors to the previous **P150**-*b*-**P255–66**, including similar average core widths of the resulting 1D nanofibers (*i.e.*, before staining: 25.8 nm for **P1100**-*b*-**P255** and 31.6 nm for **P1100**-*b*-**P266** and after staining: 34.2 nm for **P1100**-*b*-**P255** and 37.3 nm for **P1100**-*b*-**P266** by TEM imaging) (Fig. S35 and S36†). Analogous sonication produced uniform seed solutions (**P1100**-*b*-**P255**: *L*_n_ = 68.7 nm, *L*_w_/*L*_n_ = 1.18 and **P1100**-*b*-**P266**: *L*_n_ = 73.8 nm, *L*_w_/*L*_n_ = 1.15). Then, instead of the seeded growth (due to their low solubility), we adopted a self-seeding strategy: thermally induced epitaxial growth.^56–58^ In this instance, modulating the temperature after sonication provided varying concentrations of the unimer solution *in situ*, thereby controlling the 1D nanofiber lengths. The seed solutions of **P1100**-*b*-**P255** and **P1100**-*b*-**P266** in chloroform were annealed at different temperatures ranging from 34 °C to 61 °C and cooled to room temperature (RT). After 3 h, long 1D nanofibers with uniform *L*_n_, ranging from 68.7 nm to 4.6 μm for **P1100**-*b*-**P255** and from 73.8 nm to 1.14 μm for **P1100**-*b*-**P266** based on the annealing temperature, were generated with narrow dispersities (*L*_w_/*L*_n_: 1.04–1.21) (Fig. 6d–f, S37 and S38†). Additional aging of these wider 1D nanofibers for 1 d did not alter their lengths or widths, showing structural stability and the absence of end-to-end coupling (Fig. S39 and S40†). We were able to control the length of the 1D nanofibers up to 4.7 μm using living CDSA (either by seeded growth or self-seeding), and their core widths ranged from 12 to 32 nm, proportional to the DP of **P2** (Fig. S41†).

**Fig. 7:**
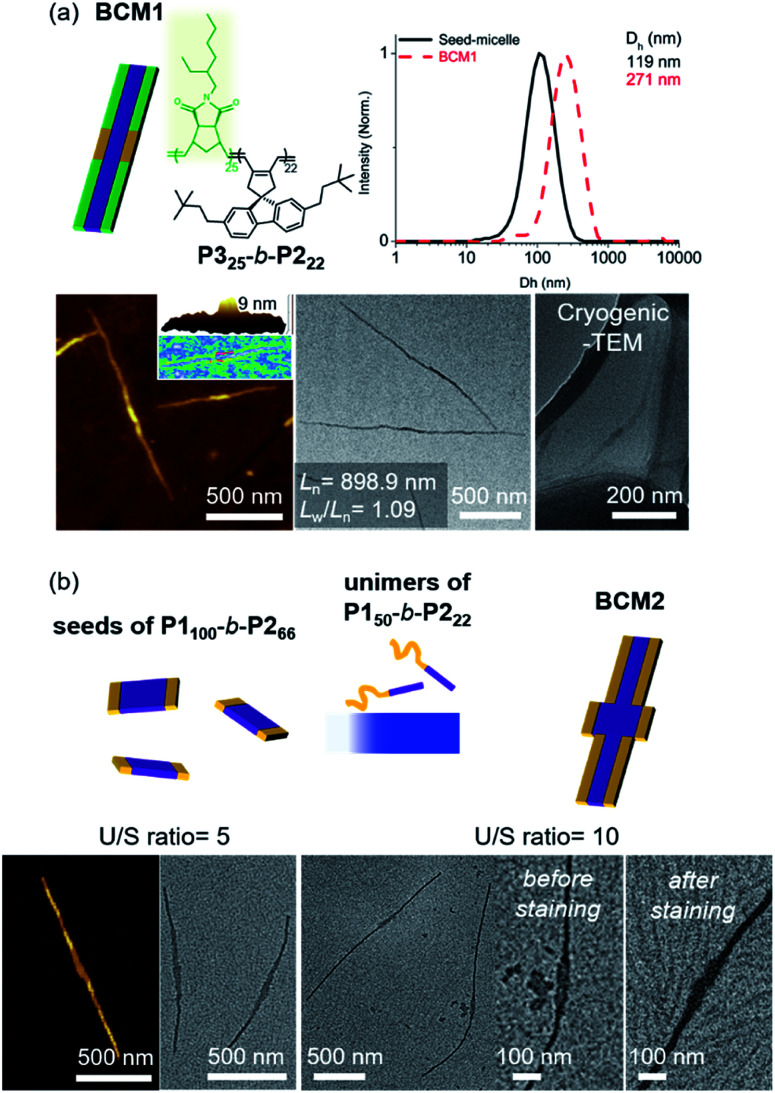
More complex **BCMs** were prepared by (a) adding a unimer **P325**-*b*-**P222** having a different block to **P150**-*b*-**P222** seed (**BCM1**) and (b) adding a unimer (**P150**-*b*-**P222**) to a wider seed (**P1100**-*b*-**P266**) to produce **BCM2** with different widths. The number in an image (a) indicates “the average *L*_n_ and its length dispersity”.

Another advantage of the living CDSA is the capability to produce more complex block comicelles (**BCMs**) by further epitaxial growth from the living crystalline ends.^58^ To prepare **BCM**, another **BCP2** (**P325**-*b*-**P222**, *M*_n_ = 17.6 kDa, *Đ* = 1.06) containing soluble polynorbornene derivatives (**P3**) was synthesized by ring-opening metathesis polymerization (Fig. S42†). This new **P325**-*b*-**P222** also underwent living CDSA to form precisely controlled 1D nanofibers with *L*_n_ ranging from 165 to 1178 nm with narrow length dispersity (<1.16) *via* the seeded growth method (Fig. S43†). Then, a 10 g L^−1^ chloroform solution of the **P325**-*b*-**P222** (U/S ratio = 5) was added to the seed-micelle solution of **P150**-*b*-**P222** in DCM (with *L*_n_ of 169 nm and *L*_w_/*L*_n_ = 1.10), and a ABA tri-**BCM** (**BCM1**) was obtained with uniform length and narrow dispersity (*L*_n_ = 899 nm and *L*_w_/*L*_n_ = 1.09). The blocky structure of **BCM1** was confirmed by AFM analysis, demonstrating a height difference (9 nm of **P150**-*b*-**P222***versus* 4 nm of **P325**-*b*-**P222**). A clear distinction in contrast was observed by both dry and cryogenic-TEM images as the middle **B** block of the fully conjugated **P150**-*b*-**P222** was darker due to their higher electron density. Moreover, the average core width of **BCM1** was consistent throughout all the nanofibers as the length of **P2** in both **P150**-*b*-**P222** and **P325**-*b*-**P222** was the same (Fig. 7a and S44†). Encouraged by the initial success, a more complex **BCM2** was prepared through addition of the unimer solution, **P150**-*b*-**P222**, to another seed solution of **P1100**-*b*-**P266** showing a wider 1D nanofiber (annealing temp. 52 °C, *L*_n_ = 213 nm). After 4 h of aging at 10 °C, another ABA tri-**BCM2** consisting of a wider middle block was identified from AFM and TEM imaging showing a difference in the width of each block depending on the length of **P2**. Note that only a single strand of the thinner **A** block grew from both ends of the **B** block despite large width differences (Fig. 7b and S45†).^22^

**Fig. 8:**
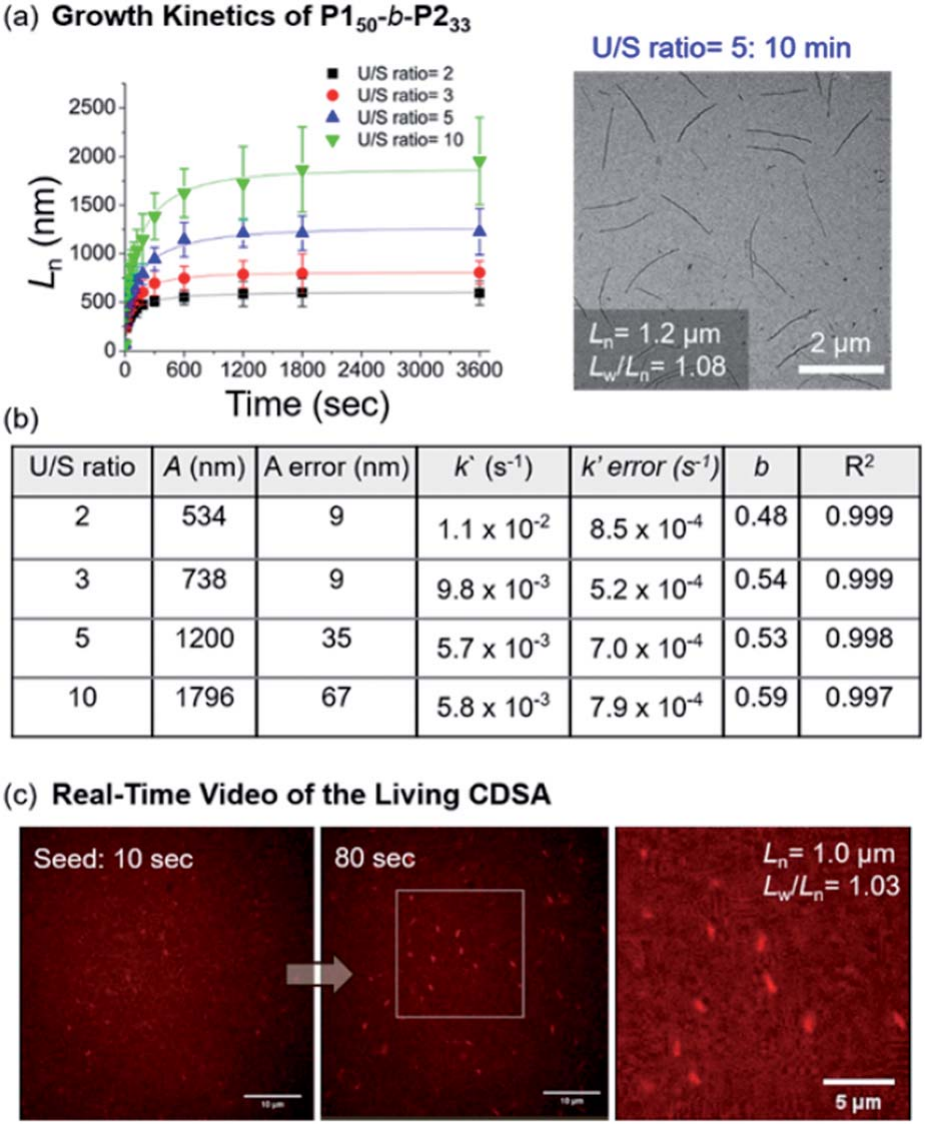
(a) Lengths (*L*_n_) of the 1D nanofibers from **P150**-b-**P233** over time (monitored over 13 h after adding the unimer solution to the seed micelles (*L*_n_ = 66.5 nm, *L*_w_/*L*_n_ = 1.16)). TEM image was obtained after 10 min with U/S ratio = 5. (b) Table of kinetic data for various U/S ratios. Standard errors for the values *A*, *k*′, and *b* were obtained from the fitting of eqn (S1).†*A* is the actual length growth obtained by *L*_n_ − *L*_seed_ (seed length). *k*′ is the rate constant. *b* is the fractional power of the exponential. (c) Representative LSCM images of Video S1† at time points of 10 and 80 s (scale bars = 10 μm). We calculated the *L*_n_ of the 1D nanofibers from those images.

Notably, this CDSA of the fully conjugated **BCP** series exemplifies excellent control of the length and width and rapid growth rate. As such, the growth kinetics of **P150**-*b*-**P233** was observed using real-time monitoring of the elongation of 1D nanofibers by TEM analysis. Upon the addition of unimers, 1D nanofibers elongated rapidly and reached constant lengths (*L*_n_ = 746 nm (U/S ratio = 3, *L*_w_/*L*_n_ = 1.03) in 10 min, and *L*_n_ = 1.2 μm (U/S ratio = 5, *L*_w_/*L*_n_ = 1.08) in just 20 min) (Fig. 8a and Table S2†). Furthermore, the kinetic data was fitted into a stretched exponential function that the Manners group previously used to describe the nanoparticle growth rates of PFS-*b*-(polydimethylsiloxane) (PFS-*b*-PDMS) (eqn (S1)†).^59^ In this study, this function also explained the growth kinetics of **P150**-*b*-**P233** well with *R*^2^ values greater than 0.997, providing *k*′ values of 11 × 10^−3^, 9.8 × 10^−3^, 5.7 × 10^−3^, and 5.8 × 10^−3^ s^−1^ for U/S ratios of 2, 3, 5, and 10, respectively. Notably, these rates are about 10 times faster than those of other typical living CDSA of 1D nanowires or comparable to the highest rates (16 × 10^−3^)^59^ under a specific condition.^52,59–62^ By taking the average values from various U/S ratios, we obtained the parameter *b* of 0.54. The deviation from the theoretical value of 1 for first-order kinetics was presumably due to the influence of the flexible chain conformation of the shell, disturbing ideal crystallization during seeded growth (Fig. 8b and S46–S50†).^59,63,64^ Regardless, we attributed the fast kinetics of the current CDSA to the intrinsically rigid conformation of the **P2** showing stretched conformation without chain folding.^41,42^

Since the 1D nanofibers contained fluorescent **P2** block and grew quickly to micron sizes, the entire CDSA could be visualized *via* a real-time video with a laser scanning confocal microscope (LSCM), even without additional dyes (Fig. S51 and S52†).^65–67^ By adding a unimer solution of **P150**-*b*-**P233** to the seed in 0.01 g L^−1^ chloroform with U/S ratio 30, the real-time motion of nanofibers elongation up to *L*_n_ = 1.0 μm (*L*_w_/*L*_n_ = 1.03) within 100 s was observed. This is the first video recording of the actual CDSA and this was only possible due to the fast seeded growth of conjugated **P150**-*b*-**P233** which also showed stable fluorescence in solution (Fig. 8c and S52†).

## Conclusions

In summary, we successfully prepared fully conjugated **BCPs** that underwent self-assembly into 1D nanofibers. Their lengths were controlled from 0.05 to 4.7 μm, utilizing the living CDSA technique *via* seeded growth or self-seeding. We were also able to tune their widths from 12 to 32 nm by modulating the DP of the core block. This excellent width control proportional to the DP of **P2** was due to the fully stretched conformation of the conjugated **P2** block without chain folding. As a result, the CDSA was rapid while maintaining excellent control of the dimensions of the nanofibers. Close monitoring of the growth kinetics revealed that the formation of micron-sized 1D nanofibers occurred in 20 min, much faster than the other CDSA cases. This rapid kinetics of CDSA producing fluorescent 1D nanofibers enabled real-time monitoring of their growth using confocal fluorescence microscopy. Lastly, this living CDSA technique enabled the preparation of more complex **BCMs**. The fast formation of fully conjugated and fluorescent nanostructures offers an efficient method for preparation of uniformly sized polymeric optoelectronic materials with controllable length and width in narrow dispersity.

## Conflicts of interest

There are no conflicts to declare.

## Supplementary Material

SC-011-D0SC02891F-s001

SC-011-D0SC02891F-s002

SC-011-D0SC02891F-s003

SC-011-D0SC02891F-s004
