# Supplementary material for: Rapid formation and real-time observation of micron-sized conjugated nanofibers with tunable lengths and widths in 20 minutes by living crystallization-driven self-assembly
Source: Chem Sci. 2020 Jul 29;11(32):8416–24. doi: 10.1039/d0sc02891f (PMC8161532; doi:10.1039/d0sc02891f)
Supplement: SC-011-D0SC02891F-s001 [file SC-011-D0SC02891F-s001.pdf]

Electronic Supplementary Material (ESI) for Chemical Science.
This journal is © The Royal Society of Chemistry 2020

SUPPORTING INFORMATION

Rapid Formation and Real-Time Observation of Micron-Sized Conjugated Nanofibers with Tunable Lengths and Widths in 20 Minutes by Living Crystallization-Driven Self-Assembly

Sanghee Yang[a], and Tae-Lim Choi*[a]

[a]*Department of Chemistry, Seoul National University, Seoul 08826, Republic of Korea*

**Email: tlc@snu.ac.kr*

Table of Contents

1. General Materials and Methods
   A. Materials....................S2
   B. General analytical methods....................S2–S3
2. Experimental Procedures
   A. Monomer preparation....................S3
   B. Polymerization procedure....................S4
   C. Self-assembly experiments in detail....................S5
   D. ^{1}H and ^{13}C NMR characterization of polymers....................S5
3. Supporting Figures and Tables....................S6–S46
4. Supporting Video Files....................S46
5. NMR Spectra for New Block Copolymers....................S47–S48
6. References.................... S49

1. General Materials and Methods

A. Materials

Without additional notes, all reagents which were commercially available from Sigma-Aldrich®, Tokyo Chemical Industry Co. Ltd., Acros Organics, Alfa Aesar® were used without further purification. Solvents for monomer synthesis were commercially obtained, but they for polymerization was distilled under Ar atmosphere. Most of the reactions were conducted under the Ar atmosphere, and monitored by thin-layer chromatography carried out on pre-coated plates (MERCK TLC silica gel 60, F_{254}). For purification, flash column chromatography was performed using MERCK silica gel 60 (0.040 ~ 0.063 mm). The Grubbs third-generation catalyst was prepared following the reported literature.[1]

B. General analytical methods

■ Characterization of substrates and polymers

NMR spectra were recorded by Varian/Oxford As-500 (500 MHz for ^{1}H and 125 MHz for ^{13}C) spectrometer and Agilent 400-MR (400 MHz for ^{1}H and 100 MHz for ^{13}C). Size exclusion chromatography (SEC) analyses were carried out with the Waters system (a 515 pump and a 2707 autosampler with a loop volume of 100 μL), Wyatt OptiLab T-rEx refractive index detector and Shodex SEC LF-804 column eluted with chloroform (SEC grade, Honeywell Burdick & Jackson). The flow rate was 1.0 mL/min and the temperature of the column was maintained at 35 °C. Samples were diluted in 0.001-0.005 wt% by chloroform and filtered through a 0.20 μm PTFE filter before using. High-resolution mass spectroscopy (HRMS) analyses were performed by JMS-700 MStation Mass Spectrometer (Japan) in the National Center for Inter-University Research Facility and by the ultra HR-ESI Q-TOF mass spectrometer (Bruker, Germany) in the Sogang Center for Research Facilities. Cyclic voltammetry (CV) measurement was carried out on a CHI 660 Electrochemical Analyzer (CH Instruments, Insc., Texas, US) at RT using a degassed ACN solution of tetrabutylammonium hexafluorophosphate (Bu_4NPF_6, 0.1 M). The CV was recorded using a glassy carbon working electrode, a reference electrode of Ag/Ag^+ (0.1 M $AgNO_3$ in acetonitrile) with a platinum wired counter electrode at a scan rate of 100 mV/s. Single crystal X-ray diffraction was performed by SuperNova Diffractometer in Research Institute of Pharmaceutical Sciences at Seoul National University (SNU). IR spectra were measured on Brucker TENSOR 27 in National Center for Inter-University Research Facility. Differential scanning calorimetry (DSC) was carried out under $N_{2(g)}$ at a scan rate of 30 °C/min for heating and 2 °C/min for cooling with a TA Instruments Q10.

■ Characterization of nanostructures

Dynamic light scattering (DLS) data were obtained with a polymer solution (1 g/L in general) in quartz glass cell (Hellma Analytics) by Malvern Zetasizer Nano-S. UV-vis spectra were obtained by Jasco Inc (UV-vis spectrometer V-650). Multimode 8 and Nanoscope V controller (Vesco Instrument) was used for atomic force microscopy (AFM) imaging. Transmission electron microscopy (TEM) imaging was performed by using JEM-2100 (JEOL) at 120 kV. The cryo-TEM analysis was carried out by using the same microscope. Film X-ray diffraction (Film-XRD) was performed by the National Instrumentation Center for Environmental Management (NICEM) at SNU using D8 Discover with GADDS (Bruker, Germany). Carl Zeiss LSM710 was used for laser scanning confocal microscopy (LSCM) with 488 and 543 nm excitation. Fluorescent image from 561 nm excitation was obtained with SP8 X STED laser from normal LSCM. More advanced super-resolution optical microscope images were obtained from ELYRA PS.1 in the National Center for Inter-University Research Facility and SIM-Nikon (N-SIM) in Biomedical Imaging Center of College of Medicine at SNU. Asymmetric flow field-flow fractionation (AF4) was used to obtain molecular weights of 1D nanoparticles with the Wyatt Eclipse® 3+, separation system DUALTEC, Wyatt OptiLab T-rEx refractive index detector, and AF4 channel units (sets of RT Membrane 10 kDa LC (Lot-No: COBA01711), and W350 (Wide) channel) eluted with chloroform (SEC grade, Honeywell Burdick & Jackson). The detector flow rate was fixed as 1.0 mL/min, and the crossflow rate could be variable from 3.0 mL/min to 0.0 mL/min.

*AFM: The atomic force microscopy experiments were performed with a thin film prepared by spin coating one drop of the polymer solution (1 mg/ml: spinning rate = 3000 rpm for 30 secs). The thin films were prepared on mica substrates.

All images were obtained on tapping mode using non-contact mode tips from Nanoworld (Pointprobe® tip, NCHR type) with a spring constant of 42 N m^{-1} and a tip radius of ≤ 8 nm.

For each sample, length, area, aspect ratio, and angle distributions of nanoparticles were calculated by measuring over 50 samples of randomly picked nanoparticles using Gatan Digital Micrograph software (TEM imaging). Values of the number-average (X_n), weight-average (X_w), and standard deviation (σ) of nanosheets were calculated as follows where N is the sample size.

$$L_n = \frac{\sum_{i=1}^{n} N_i L_i}{\sum_{i=1}^{n} N_i} \quad L_w = \frac{\sum_{i=1}^{n} N_i L_i^2}{\sum_{i=1}^{n} N_i L_i} \qquad \sigma = \sqrt{\frac{1}{N}\sum_{i=1}^{n}(x_i - \mu)^2}$$

$$A_n = \frac{\sum_{i=1}^{n} N_i A_i}{\sum_{i=1}^{n} N_i} \quad A_w = \frac{\sum_{i=1}^{n} N_i A_i^2}{\sum_{i=1}^{n} N_i A_i}$$

2. Experimental Procedures

A. Monomer preparation

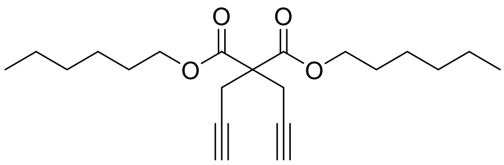

M1

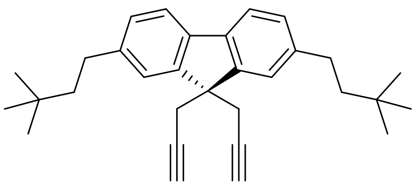

M2

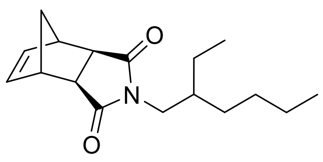

M3

These monomers (**M1**–**M3**) were prepared by the synthetic methods from previous literature .[1-3]

M1: ^{1}H NMR (500 MHz, $CDCl_3$): δ 0.89 (t, 6H), 1.30 (m, 12H), 1.61 (q, 4H), 3.00 (d, 4H), 4.16 (t, 4H); ^{13}C NMR (125 MHz, $CDCl_3$): δ 14.2, 22.7, 22.8, 25.6, 28.6, 31.5, 56.6, 66.4, 71.9, 78.7, 168.9; HRMS (CI+): calcd. for $C_{21}H_{33}O_4$, 349.2379; found, 349.2375.

M2: ^{1}H NMR (500 MHz, $CDCl_3$): δ 7.57 (d, J = 7.5 Hz, 2H), 7.55 (s, 2H), 7.19 (d, J = 7.6 Hz, 2H), 2.82 (d, J = 2.3 Hz, 4H), 2.69–2.60 (m, 4H), 2.04 (s, 2H), 1.55 (m, 4H), 0.98 (s, 18H); ^{13}C NMR (100 MHz, $CDCl_3$): δ 148.8, 142.5, 137.7, 128.2, 123.9, 119.5, 81.4, 70.7, 49.7, 46.6, 31.7, 30.8, 29.5, 27.7; IR: 3302, 3281, 2945, 2861, 2120, 1469, 1364, 1246, 821, 762 cm^{-1}; HR-MS (ESI) [Na+] calcd. for $C_{31}H_{38}Na$, 433.2871; found, 433.2866.

M3: ^{1}H NMR (400 MHz, $CDCl_3$): δ 0.86 (t, 6H), 1.25 (m, 9H), 1.50 (d, 1H), 1.68 (m, 1H), 2.65 (s, 2H) 3.25 (s, 2H), 3.34 (d, 2H), 6.26 (s, 2H); ^{13}C NMR (100 MHz, $CDCl_3$): δ 10.3, 14.0, 22.9, 23,9, 28.4, 30.5, 37.7, 42.7, 45.1, 47.7, 137.8, 178.4. HRMS (FAB+): calcd. for $C_{16}H_{23}NO_2$, 276.1964; found, 276.1960.

B. Polymerization procedure

A 4 mL sized screw-cap vial with septum was flame dried and charged with a monomer and a magnetic bar. The vial was purged with $Ar_{(g)}$ four times, and degassed anhydrous THF was added ([**M1**]$_0$ = 0.5 M, or [**M3**]$_0$ = 0.5 M). After the Ar-purged Grubbs third-generation catalyst in the other 4 mL vial was dissolved in THF, the solution was rapidly injected to the monomer solution at 0 °C under vigorous stirring. After the complete conversion of **M1** to **P1,** or **M3** to **P3**, the second monomer (**M2**) was added ([**M2**]$_0$ = 0.1 M) to the vial at 0 °C.

1) Quenching and purification: The reaction was quenched by excess ethyl vinyl ether (EVE) after the desired reaction time and precipitated in methanol at room temperature. The obtained purple solid was filtered and dried *in vacuo*. The conversion of monomer was calculated from the ^{1}H NMR spectra of the crude mixture.[1-3]

2) NMR analysis of crude mixture for calculating the conversion of monomers (Table 1): After quenched by excess amounts of EVE, one 10 μL aliquots of the crude mixture were dried *in vacuo* and diluted in $CDCl_3$ for calculating conversion (the remaining reaction solution was precipitated in MeOH to obtain the isolated polymer powders). The monomer conversion was determined by using the reference peak in ^{1}H NMR of the crude solution (before removing unreacted monomers), and the polymer peak overlapping with the same of monomer was taken as a theoretical value, and the other remaining monomer peak which was not overlapped with its polymer peak was calculated.

3) INCP experiments (Fig. S7 and S20): In the case of *in situ* TEM sampling during the polymerization process, the 20 μL aliquots (THF solution) were taken out from the solution at different times using microsyringes, and diluted with THF or chloroform to 1 g/L after quenching by EVE. DLS, UV-vis analyses and TEM samplings were conducted with the *in situ* 1 g/L solutions. All experiments from **Fig**. **S21** to **Fig S52** were conducted with precipitated **BCPs**.

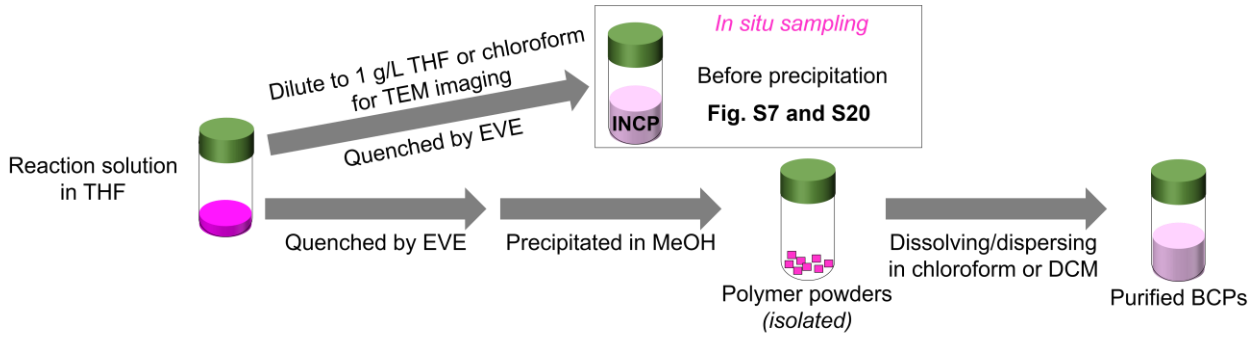

C. Self-assembly experiments in detail

■ Preparation of 1D nanofibers of **BCPs**

The solutions of the purified **BCPs** in chloroform or DCM (1 g/L, 1 mL in 4 mL vial) was sealed with Teflon lined cap. In some cases, they were aged in the fume hood at 25 °C for a day to form 1D nanofibers.

■ Seeded growth experiment

The unimer solutions, **BCPs** in chloroform (10 g/L) in general, were added to the seed solution (prepared by sonication) with various unimer-to-seed ratios (in chloroform or DCM, 0.1 g/L).

■ Self-seeding experiment

The seed solutions of **BCPs** in chloroform or DCM (0.1 g/L, 1 mL in 4 mL vial) was sealed with Teflon lined cap and was heated in vial heating block at various temperatures (40 °C - 61 °C) for 30 min. Then, the heated **BCPs** were aged in the fume hood at 25 °C.

D. ^{1}H and ^{13}C NMR characterization of polymers for each block

Each homopolymer has been characterized separately in three different reference.[1-3]

■ **P1** homopolymer

^{1}H NMR (500 MHz, $CDCl_3$): δ 6.67–6.30 (br m, 2H), 4.23–4.05 (br m, 4H), 3.42–3.17 (br m, 4H), 1.75–1.60 (br m, 4H), 1.30 (br m, 12H), 0.88 (br m, 6H); ^{13}C NMR (125 MHz, $CDCl_3$): δ 171.6, 137.1, 123.1, 77.5, 65.9, 65.8, 41.4, 31.4, 28.4, 25.4, 22.5, 13.8.[1]

■ **P2** homopolymer

^{1}H NMR (500 MHz, $CDCl_3$): δ 7.34 (br m, 2H), 7.15–6.90 (br m, 4H), 6.59 (br m, 2H), 3.04 (br m, 4H), 2.60–2.18 (br m, 4H), 1.36 (br m, 4H), 1.00–0.72 (br m, 18H); ^{13}C NMR (125 MHz, $CDCl_3$): δ 153.7, 142.6, 139.3, 136.9, 126.9, 123.4, 122.0, 119.0, 52.9, 46.8, 46.1, 31.3, 30.5, 29.3.[2]

■ **P3** homopolymer

^{1}H NMR (500 MHz, $CDCl_3$): δ 5.80–5.65 (br, 1H), 5.60–5.40 (br, 1H), 3.20–2.90 (br m, 4H), 2.85–2.55 (br m, 2H), 2.34–1.95 (br m, 1H), 2.60–2.18 (br m, 1H), 1.80–1.40 (br m, 2H), 1.40–1.00 (br m, 12H), 0.95–0.75 (br m, 6H); ^{13}C NMR (125 MHz, $CDCl_3$): δ 178.5, 133.5, 131.7, 52.9, 52.5, 50.9, 42.5, 42.3, 41.0, 37.3, 30.5, 28.4, 23.7, 23.0, 14.1, 10.3.[3]

3. Supporting Figures and Tables

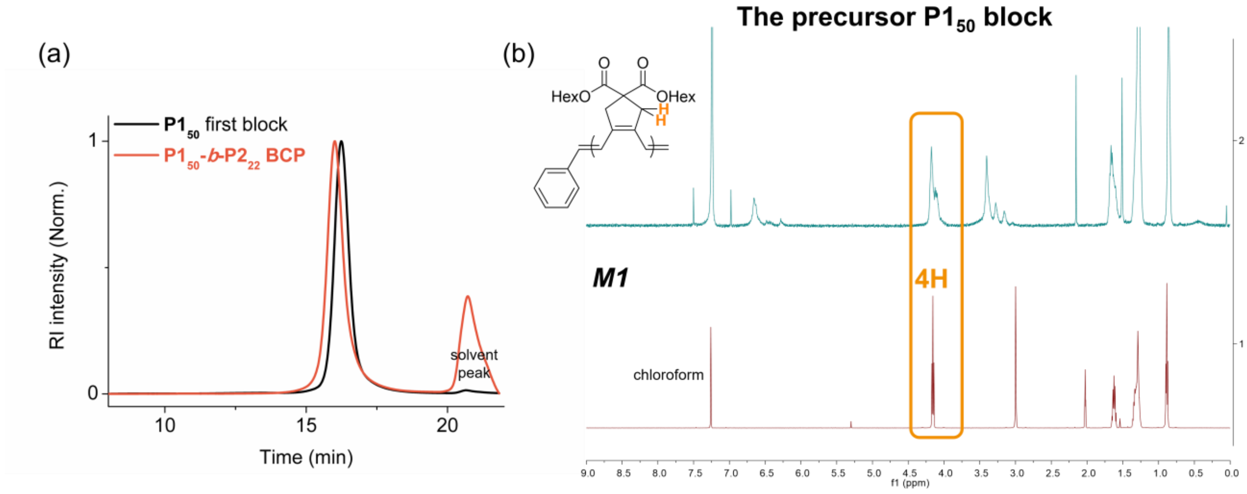

Fig. S1 (a) A normalized chloroform SEC traces of the precursor **P1$_{50}$** first block (M_n= 25.5 kDa ($Đ$= 1.08) and **P1$_{50}$**-*b*-**P2$_{22}$** (M_n= 36.3 kDa ($Đ$= 1.07). (b) ^{1}H NMR spectra of the **M1** and **P1$_{50}$** block in chloroform-*d* at 20 °C. Regardless of the conversion of **M1**, the position of 4H on both **M1** and **P1** can be set to four in the spectra, because their position does not change during polymerization. The protons can be used as a basis for calculating the block ratios of **P1$_{50}$**-*b*-**P2$_n$**.

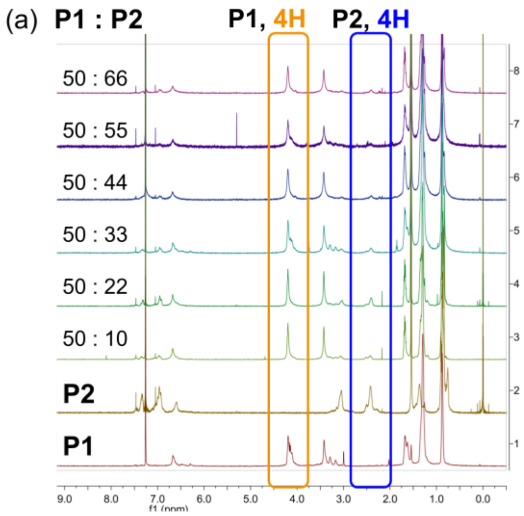

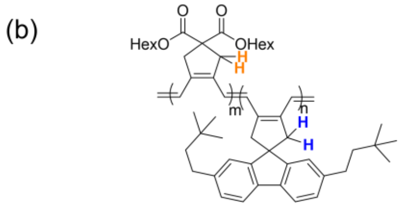

(c)

CDCl$_3$	**P2** block intensity (4H)
	Initial % of **P2** signals
**P1$_{50}$**-*b*-**P2$_{10}$**	100
**P1$_{50}$**-*b*-**P2$_{22}$**	100
**P1$_{50}$**-*b*-**P2$_{33}$**	28.4
**P1$_{50}$**-*b*-**P2$_{44}$**	16.8
**P1$_{50}$**-*b*-**P2$_{55}$**	10.9
**P1$_{50}$**-*b*-**P2$_{66}$**	11.4

Fig. S2 (a) ^{1}H NMR spectra of initial **P1$_{50}$**-*b*-**P2$_n$** (n = 10–66) (after purification) in chloroform-*d* at 20 °C. (b) Protons corresponding to the two boxes on the spectra were represented on the polymer structures. (c) Percentage is relative **P2**% compared with expected integration from the feed ratios. For the NMR analysis, 1 g/L deuterated solutions were prepared and analyzed with 2 sec relaxation time and 64 scan numbers.

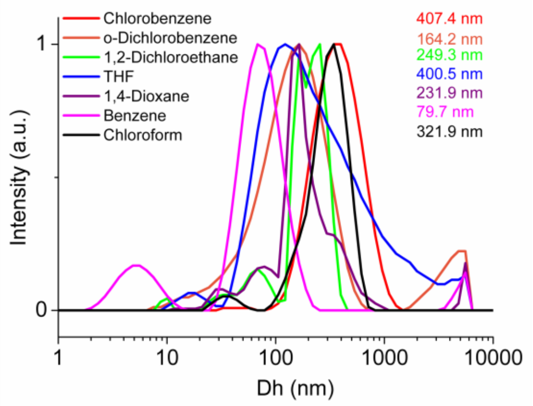

Fig. S3 (a) DLS profiles of **P1**$_{50}$-*b*-**P2**$_{44}$ at 0.1 g/L in various solvents before aging. D_h values indicating the formation of nanoparticles were observed in all initial solutions. For the analysis, the **BCPs** were prepared after purification.

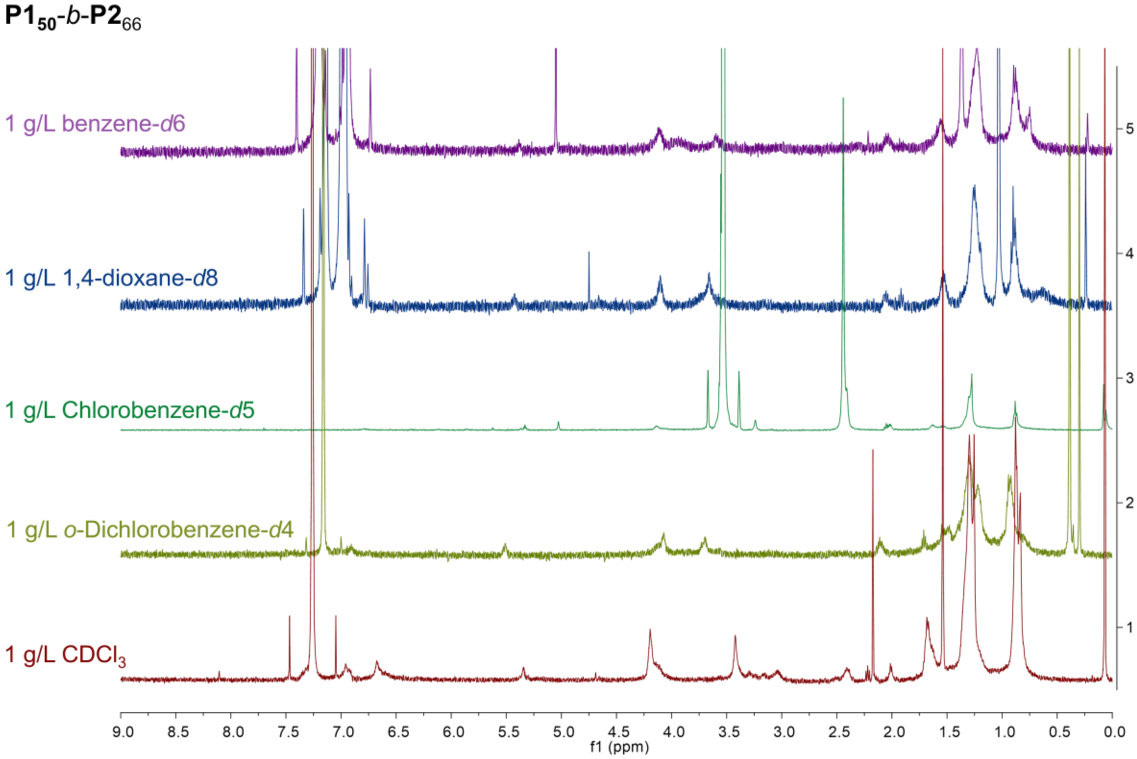

Fig. S4 ^{1}H NMR spectra of various **P1**$_{50}$-*b*-**P2**$_{66}$ in various deuterated solvents at 20 °C. For the NMR analysis, 1 g/L deuterated solutions were prepared and analyzed with 2 sec relaxation time and 128 scan numbers. Chloroform-*d* was still the best solvent for the **BCPs**. For the analysis, the **BCPs** were prepared after purification.

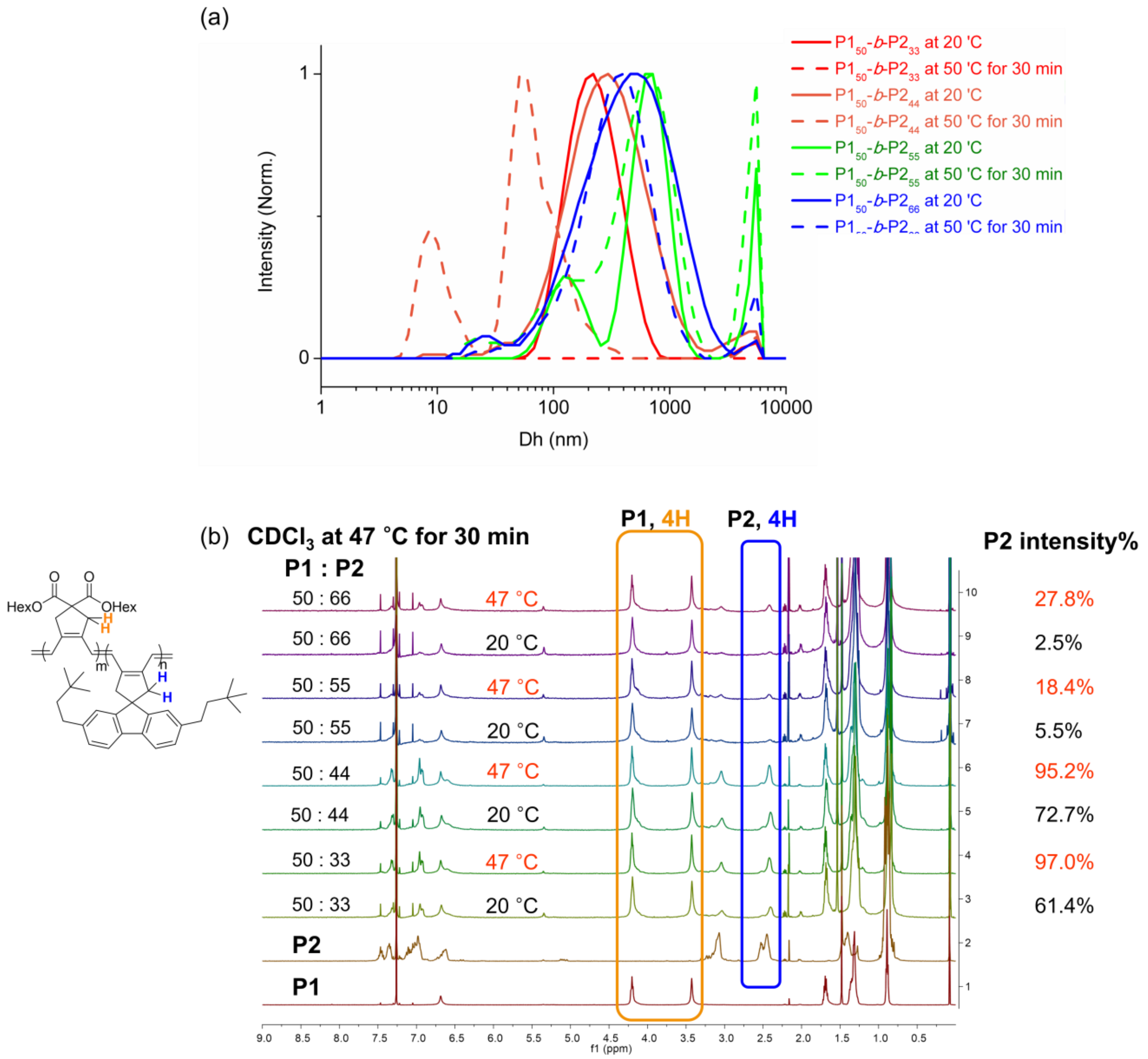

Fig. S5 (a) DLS profiles of **P1$_{50}$**-*b*-**P2$_{33}$** to **P1$_{50}$**-*b*-**P2$_{66}$** at 20 °C and 50 °C at 0.1 g/L in chloroform. At each temperature, the solutions were heated at 50 °C for 30 min, and the D_h of the **P1$_{50}$**-*b*-**P2$_{33}$** dissapeared and that of **P1$_{50}$**-*b*-**P2$_{44}$** decreased as less than 100 nm after heating; however, even after heating, the longer **BCPs** still showed large D_h values. (b) ^{1}H NMR spectra of **P1$_{50}$**-*b*-**P2$_n$** (n = 33–66) before and after heating up to 47 °C in chloroform-*d* for 30 min to compare the relative percentage of **P2**% at different temperatures. For the NMR analysis, 1 g/L deuterated solutions were prepared and analyzed with 2 sec relaxation time and 128 scan numbers. The relative **P2**% (from **Fig. S2**) was close to 100% in **P1$_{50}$**-*b*-**P2$_{33}$** and **P1$_{50}$**-*b*-**P2$_{44}$**, and longer **BCPs** showed only 27.8% and 18.4% integration then expected values. Fortunately, more quantitative analysis was possible for **P1$_{50}$**-*b*-**P2$_{33}$** and **P1$_{50}$**-*b*-**P2$_{44}$** due to better solubility of **P2** at the higher temperature. For the analysis, the **BCPs** were prepared after purification.

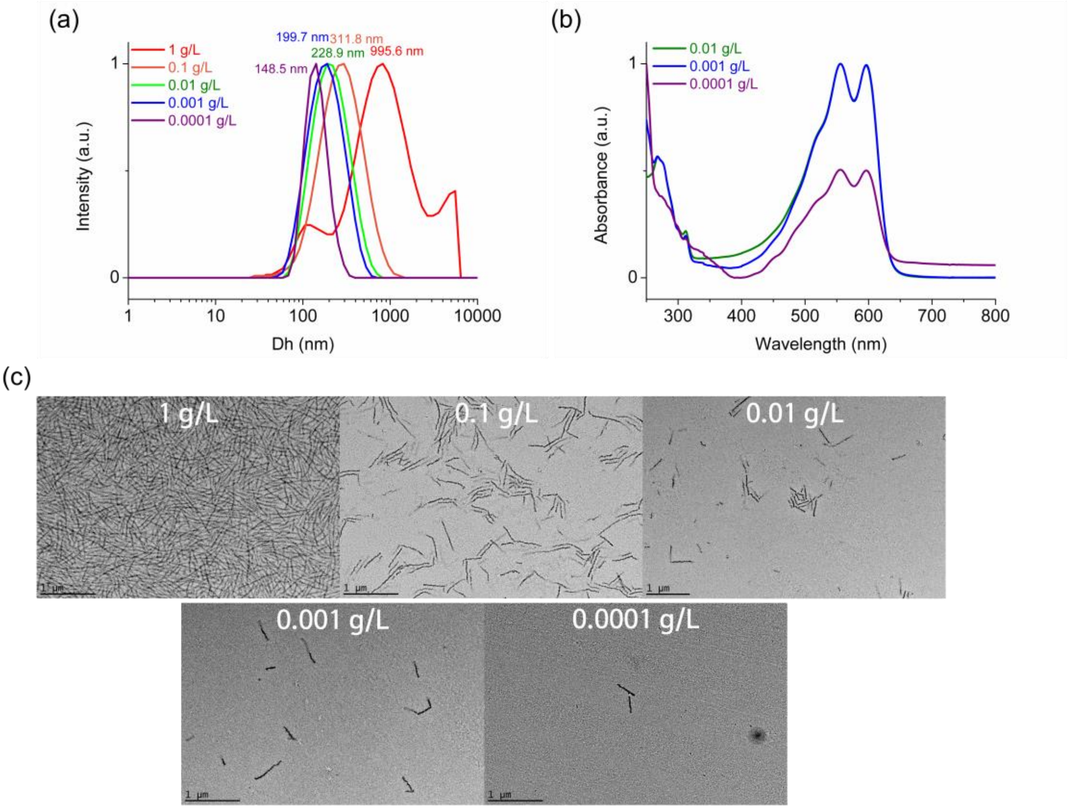

Fig. S6 (a) DLS profiles, (b) UV-vis absorbance spectra, and (c) TEM images of **P1**$_{50}$-*b*-**P2**$_{66}$ at various concentrations in chloroform. The 1D nanofibers were observed at 0.0001 g/L, indicating that the self-assembled structures were still stable at low concentrations. For the analysis, the **BCPs** were prepared after purification.

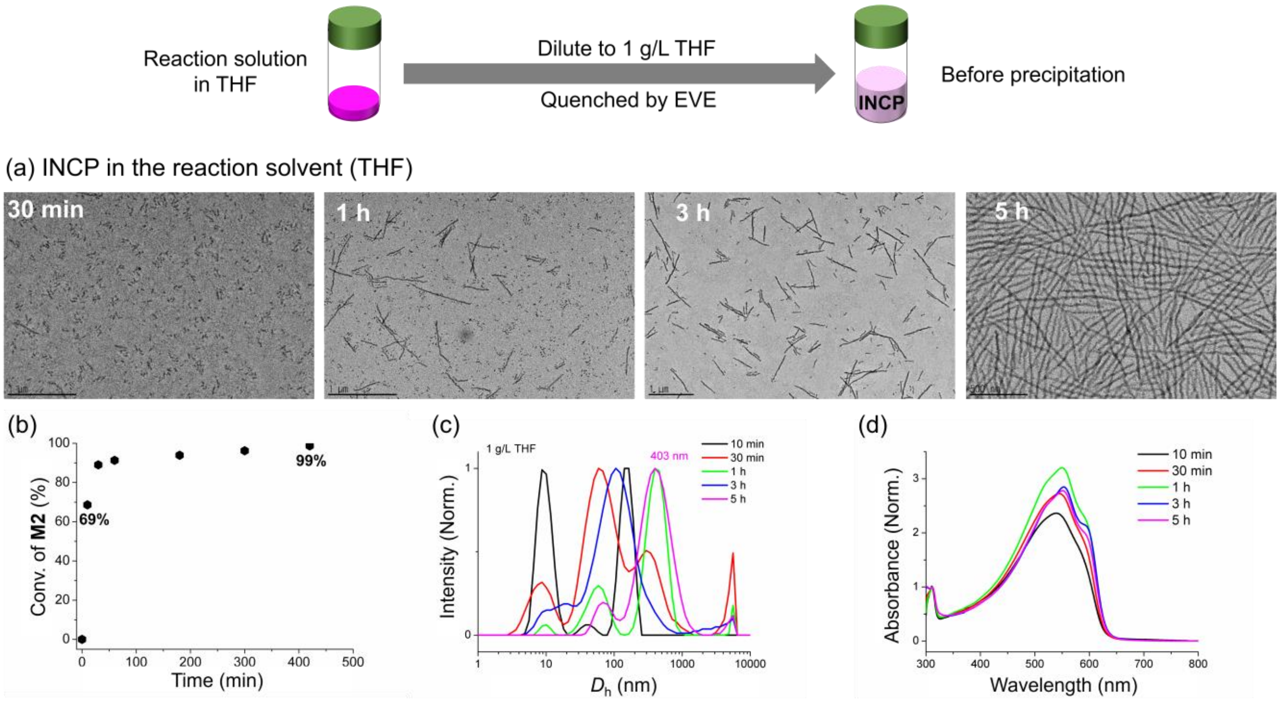

Fig. S7 (a) The *in situ* TEM images of **P1**$_{50}$-*b*-**P2**$_{66}$ at 1 g/L in THF (reaction solvent) by direct sampling during polymerization at 30 min (69% conv.), 1 hour, 3 hours, and 5 hours (99% conv.) (before precipitation). (b) A plot of the monomer (**M2**) conversion (%) vs. the polymerization time of **P1**$_{50}$-*b*-**P2**$_{66}$ from ^{1}H NMR analysis in $CDCl_3$ before purification. Changes of (c) DLS profiles, and (d) UV-vis absorbance spectra at the polymerization time points.

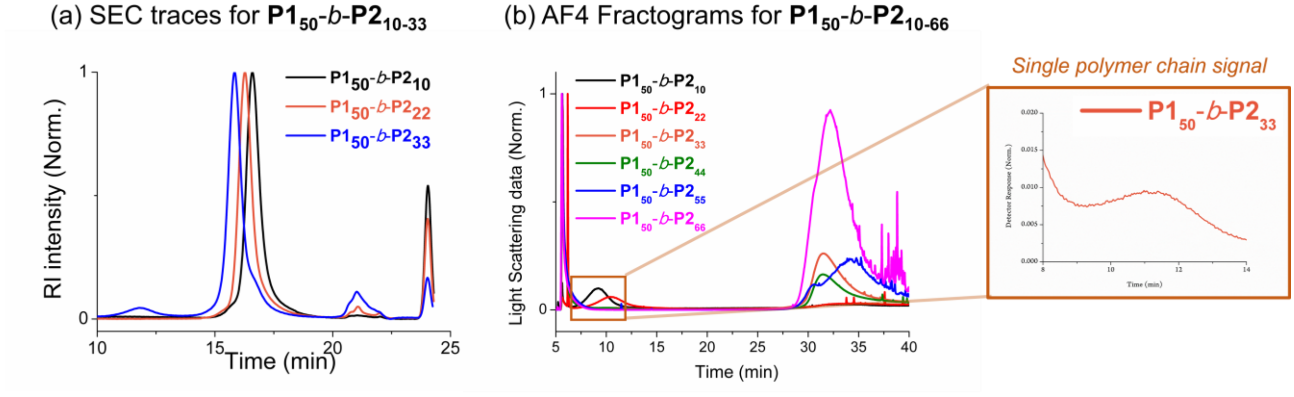

(c) Molecular characterization by chloroform-SEC and chloroform-FFF (AF4)

BCPs	$P1_{50}$-*b*-$P2_{10}$		$P1_{50}$-*b*-$P2_{22}$		$P1_{50}$-*b*-$P2_{33}$			$P1_{50}$-*b*-$P2_{44}$	$P1_{50}$-*b*-$P2_{55}$	$P1_{50}$-*b*-$P2_{66}$
Methods	SEC	AF4	SEC	AF4	SEC	AF4				
	Single polymer						Aggregation			
M_n (kDa)	23.3	26.9	38.6	42.2	47.4	53.5	15050	10440	70350	115800
M_w (kDa)	26.8	27.6	42.5	42.9	53.5	55.5	17320	16300	88850	130400
Đ	1.15	1.02	1.10	1.02	1.13	1.04	1.15	1.56	1.26	1.13
R_z (nm)		0		9.8		19.7	121.8	132.9	146.4	129.7

Fig. S8 (a) Normalized chloroform SEC traces of smaller **BCPs** (**P1**$_{50}$-*b*-**P2**$_{10}$–**P1**$_{50}$-*b*-**P2**$_{33}$) in **Table 1**, (b) AF4 traces of all **BCPs** including larger **P1**$_{50}$-*b*-**P2**$_{44}$–**P1**$_{50}$-*b*-**P2**$_{66}$, and (c) The M_n values of smaller **P1**$_{50}$-*b*-**P2**$_{10}$–**P1**$_{50}$-*b*-**P2**$_{33}$ obtained by either method. Each polymer solution was filtered through a 0.2 μm pTFE filter (1 g/L of polymer solution) for SEC analysis, and a 1.0 μm pTFE filter (10 g/L) for AF4 analysis. Due to the aggregation of larger **BCPs** in initial chloroform, larger filters were used. In AF4 traces, the huge differences in elution time of **BCPs** were caused by huge differences in M_n: around 10 min for **P1**$_{50}$-*b*-**P2**$_{10}$–**P1**$_{50}$-*b*-**P2**$_{33}$ and around 30 min for **P1**$_{50}$-*b*-**P2**$_{33}$–**P1**$_{50}$-*b*-**P2**$_{66}$. The M_n values for larger **BCPs** showed massive M_ns with MDa unit and directly stood for nanoparticlization, and those of smaller **BCPs** obtained by either method were similar; it implies that the M_n from AF4 analysis were reliable. For the analysis, the **BCPs** were prepared after purification.
(Detector flow: 1.0 mL/min and Crossflow: 3.0 mL/min for 25 min then 0 mL/min for 10 min with additional 5 min for injection).[4]

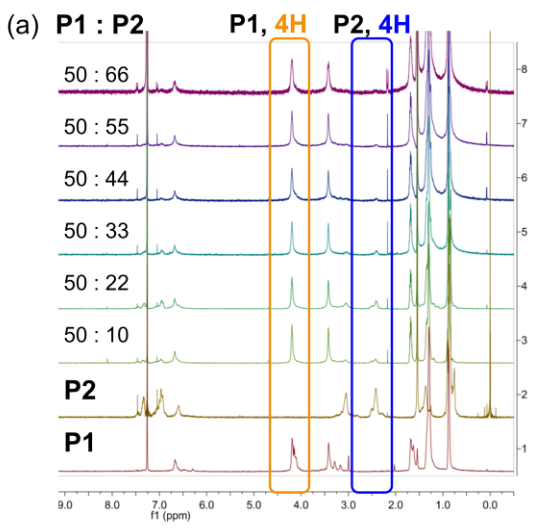

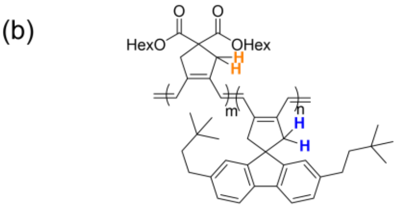

(c)

$CDCl_3$	P2 block intensity (4H)	
	Initial%	Aged%
$P1_{50}$-*b*-$P2_{10}$	100	90
$P1_{50}$-*b*-$P2_{22}$	100	73.9
$P1_{50}$-*b*-$P2_{33}$	28.4	20.1
$P1_{50}$-*b*-$P2_{44}$	16.8	0.3
$P1_{50}$-*b*-$P2_{55}$	10.9	1.4
$P1_{50}$-*b*-$P2_{66}$	11.4	0.6

Fig. S9 (a) ^{1}H NMR spectra of **P1**$_{50}$-*b*-**P2**$_n$ (n = 10–66) (after purification) in chloroform-*d* at 20 °C after 1 day aging. (b) Protons corresponding to the two boxes on the spectra were represented on the polymer structures. (c) The relative **P2**% (from **Fig. S2**) decreased after 1 day aging.

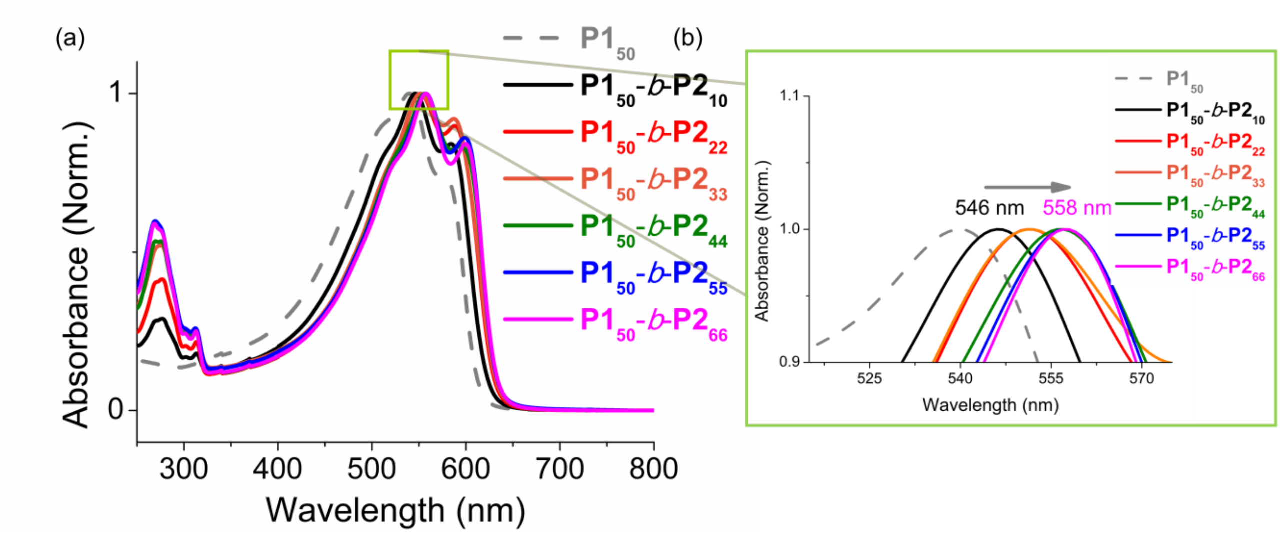

Fig. S10 (a) UV-vis absorbance spectra of **P1₅₀** and **P1₅₀**-*b*-**P2ₙ** (after purification) in 0.05 g/L chloroform, and (b) a magnified image showing their λ_{max} values. The λ_{max} increased according to the DP of **P2** block in **BCPs**.

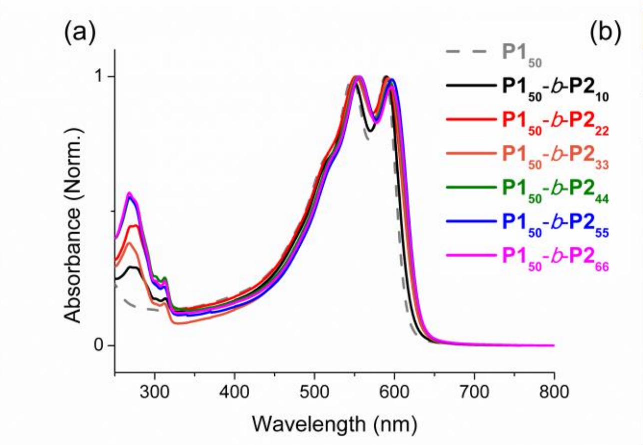

(b)

Entry	Onset (nm)	E_g (eV)
**P1$_{50}$**	620	1.99
**P2$_{22}$**	632	1.96
**P1$_{50}$-*b*-P2$_{10}$**	627	1.97
**P1$_{50}$-*b*-P2$_{22}$**	633	1.96
**P1$_{50}$-*b*-P2$_{33}$**	633	1.96
**P1$_{50}$-*b*-P2$_{44}$**	634	1.95
**P1$_{50}$-*b*-P2$_{55}$**	635	1.95
**P1$_{50}$-*b*-P2$_{66}$**	635	1.95

Fig. S11 (a) UV-vis absorbance spectra of **P1$_{50}$** and **P1$_{50}$**-*b*-**P2$_n$** (after purification) after 1 day aging in 0.05 g/L chloroform, and (b) their optical bandgaps (E_g) from the spectra in (a). All E_g values were in the semiconductor range from 0.25 to 2.5 eV. (We aged 1 g/L chloroform solutions for 1 day then diluted from 1 g/L to 0.05 g/L to analyze by UV-vis absorbance spectroscopy.)

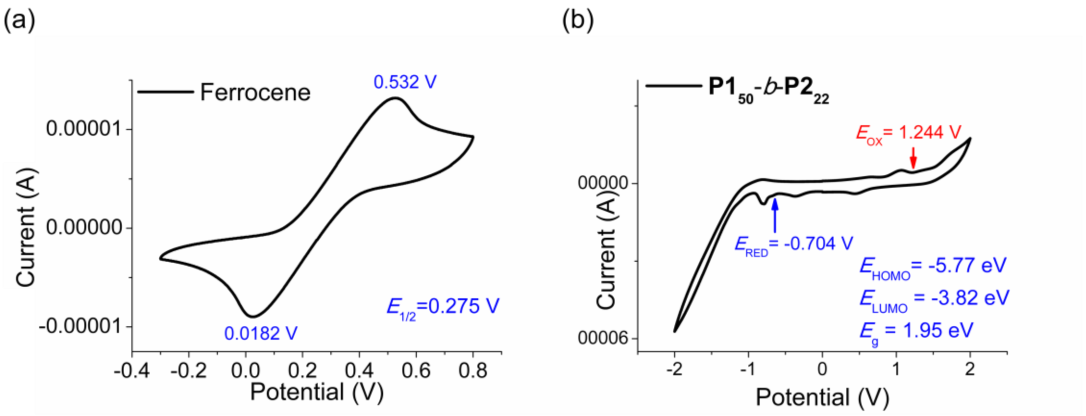

Fig. S12 (a) Cyclic voltammograms of ferrocene (a reference material, $E_{1/2}$ (Ferrocene) = 0.275 V) and (b) 1D nanofibers from **P1$_{50}$**-*b*-**P2$_{22}$** in 1 g/L chloroform solution with the Bu_4NPF_6 electrolyte after degassing to decrease the signals of O_2 gas. The E_g value was 1.95 eV being coincident with the calculated E_g = 1.96 eV from the UV-vis absorbance spectrum in **Fig. S11**. For the analysis, the **BCPs** were prepared after purification.

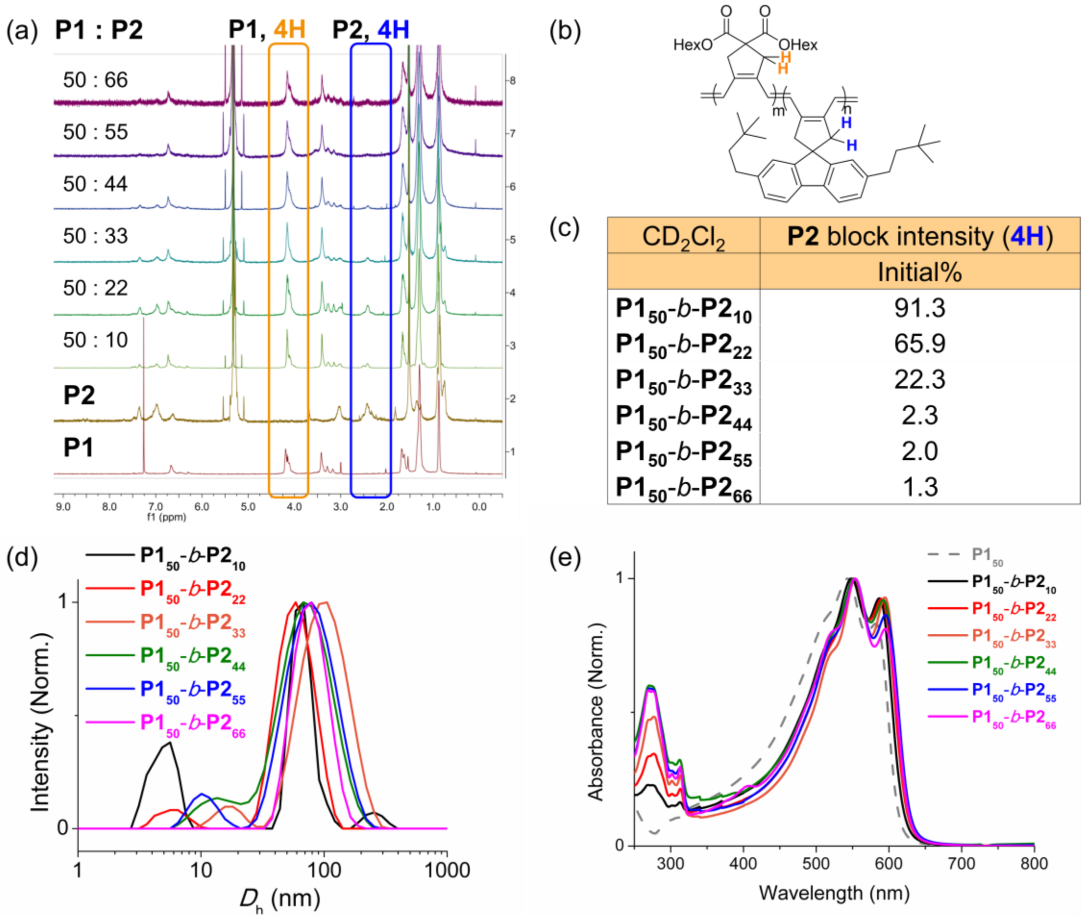

CD_2Cl_2	**P2** block intensity (**4H**)
	Initial%
**P1$_{50}$**-*b*-**P2$_{10}$**	91.3
**P1$_{50}$**-*b*-**P2$_{22}$**	65.9
**P1$_{50}$**-*b*-**P2$_{33}$**	22.3
**P1$_{50}$**-*b*-**P2$_{44}$**	2.3
**P1$_{50}$**-*b*-**P2$_{55}$**	2.0
**P1$_{50}$**-*b*-**P2$_{66}$**	1.3

Fig. S13 (a) ^{1}H NMR spectra of initial **P1$_{50}$**-*b*-**P2$_n$** (n = 10–66) (after purification) in DCM-d_2 at 20 °C. (b) Protons corresponding to the two boxes on the spectra were represented on the polymer structures. (c) The relative **P2**% (from **Fig. S2**) decreased in DCM-d_2 than chloroform-*d*. (d) DLS profiles, and (e) UV-vis absorbance spectra of **P1$_{50}$** and **P1$_{50}$**-*b*-**P2$_n$** in initial 0.05 g/L DCM.

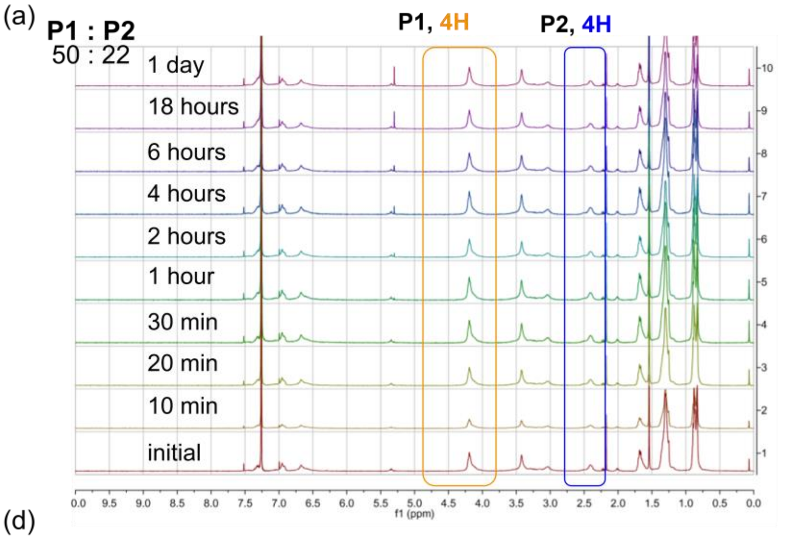

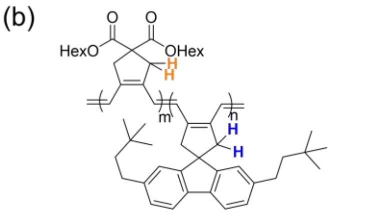

(c)

P2 block intensity (4H)			
Initial % of P2 signals			
**0 h**	100	**2 hours**	89.8
**10 min**	98.3	**4 hours**	84.7
**20 min**	96.6	**6 hours**	84.1
**30 min**	92.0	**18 hours**	78.4
**1 hour**	89.8	**1 day**	76.7

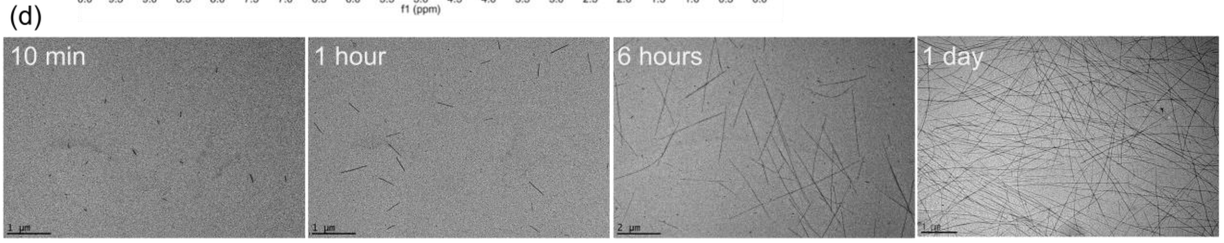

Fig. S14 (a) Time-dependent change of ^{1}H NMR spectra of purified **P1**$_{50}$-*b*-**P2**$_{22}$ in 1 g/L chloroform-*d* at 20 °C. (b) Protons corresponding to the two boxes on the spectra were represented on the polymer structures. (c) The relative **P2**% (from **Fig. S2**) decreased continuously with aging, because the **P2** block was hidden as the nanostructure was formed. d) TEM images after aging 10 min, 1 hour, 6 hours, and 1 day, which supported the formation of nanostructures.

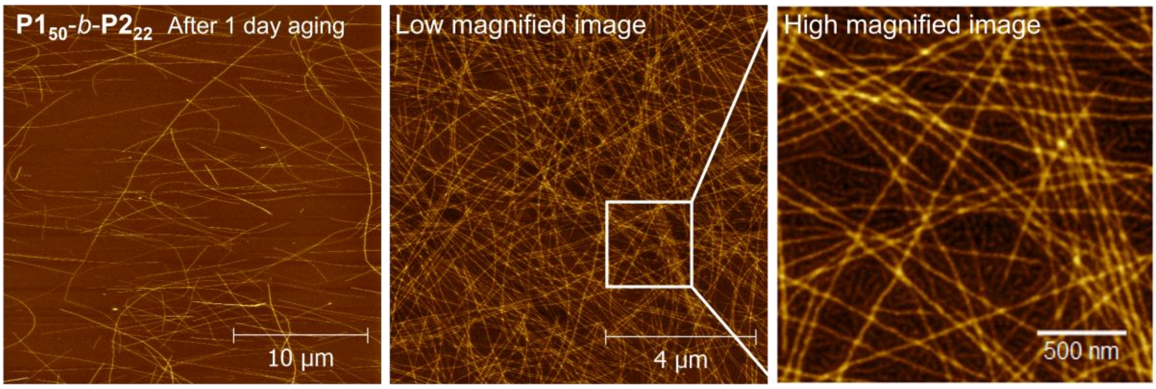

Fig. S15 AFM height images (spin-coated on mica) of 1D nanofibers from purified **P1**$_{50}$-*b*-**P2**$_{22}$ in 1 g/L chloroform after 1 day aging at 25 °C. A maximum length of the 1D nanofibers was longer than 20 μm without any branching (see **Fig. 2b**), and the 1D nanofibers was not deformed by a AFM probing tip, even if the same area was imaged several times with various magnifications.

(a)

Entry	H_n (nm)	H_w/H_n	σ/H_n	σ (nm)
**P1**$_{50}$-*b*-**P2**$_{22}$	4.82	1.03	0.16	0.79
**P1**$_{50}$-*b*-**P2**$_{33}$	3.87	1.10	0.31	1.21
**P1**$_{50}$-*b*-**P2**$_{44}$	3.64	1.08	0.27	1.00
**P1**$_{50}$-*b*-**P2**$_{55}$	5.47	1.03	0.18	0.98
**P1**$_{50}$-*b*-**P2**$_{66}$	4.29	1.07	0.27	1.15

(b)

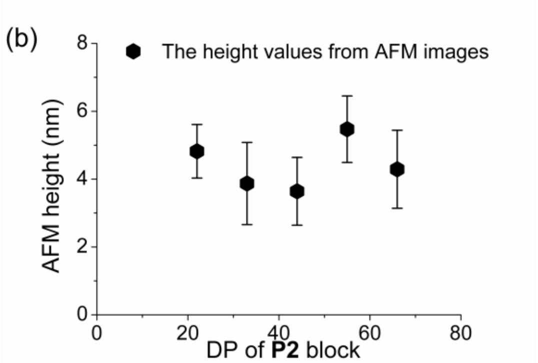

(c)

Entry	W_n (nm)	W_w/W_n	σ/W_n	σ (nm)
**P1**$_{50}$-*b*-**P2**$_{22}$	26.0	1.02	0.14	3.74
**P1**$_{50}$-*b*-**P2**$_{33}$	27.2	1.01	0.12	3.12
**P1**$_{50}$-*b*-**P2**$_{44}$	32.8	1.02	0.15	4.96
**P1**$_{50}$-*b*-**P2**$_{55}$	53.6	1.01	0.10	5.31
**P1**$_{50}$-*b*-**P2**$_{66}$	64.7	1.03	0.16	10.3

(d)

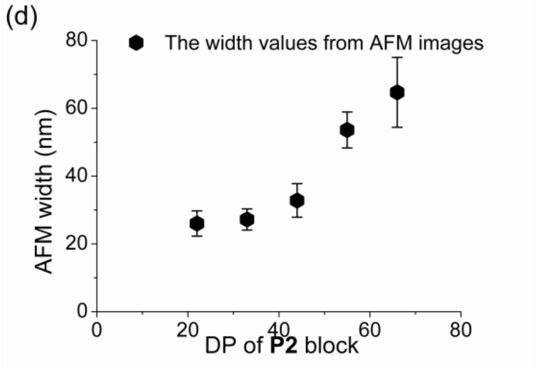

Fig. S16 (a) Table of average height (H_n) of the 1D nanofibers from AFM images, and (b) a plot of the average height (H_n) vs. DP of **P2** block in **P1**$_{50}$-*b*-**P2**$_n$. (c) Table of average width (W_n) of the 1D nanofibers from AFM images, and (d) a plot of the average width (W_n) vs. the DP of **P2** block. Error bars indicate standard deviations (σ).

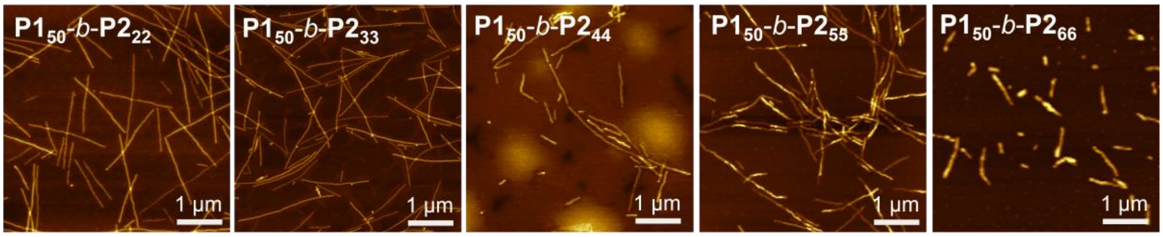

Fig. S17 AFM height images (spin-coated on mica) of 1D nanofibers from purified **P1**$_{50}$-*b*-**P2**$_n$ (n = 22–66) in 0.05 g/L chloroform without aging (only 1 day aging for **P1**$_{50}$-*b*-**P2**$_{22}$). The rigid micron-sized 1D nanofibers were observed in the diluted solution as well.

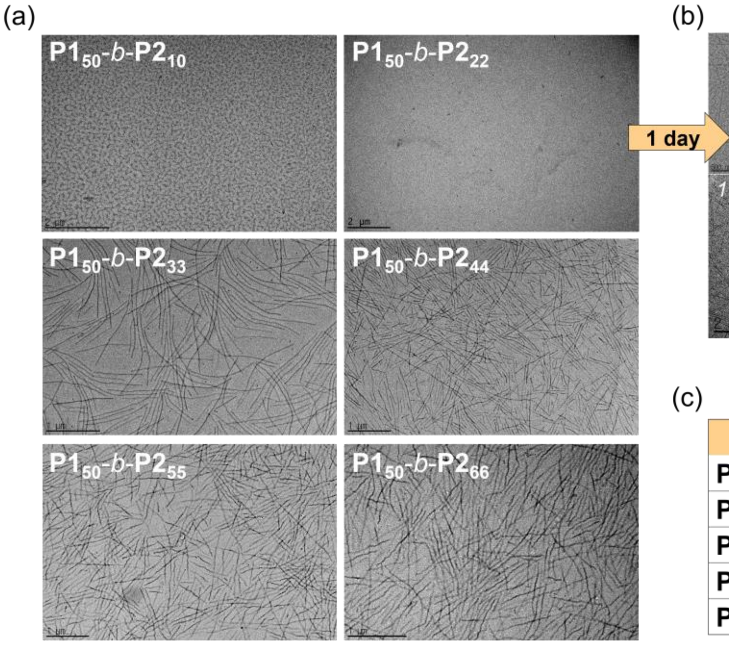

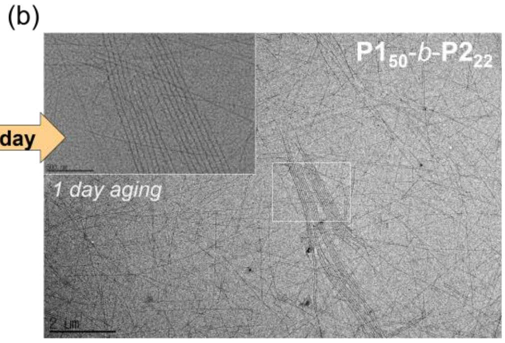

(c)

Entry	W_n (nm)	W_w/W_n	σ/W_n	σ (nm)
**P1**$_{50}$-*b*-**P2**$_{22}$	11.7	1.02	0.16	1.83
**P1**$_{50}$-*b*-**P2**$_{33}$	17.2	1.01	0.12	2.02
**P1**$_{50}$-*b*-**P2**$_{44}$	18.7	1.00	0.05	0.98
**P1**$_{50}$-*b*-**P2**$_{55}$	24.5	1.01	0.11	2.78
**P1**$_{50}$-*b*-**P2**$_{66}$	27.0	1.01	0.07	1.99

Fig. S18 (a) Low-magnified TEM images of the 1D nanofibers from **P1**$_{50}$-*b*-**P2**$_n$ in 1 g/L chloroform without aging, and (b) from **P1**$_{50}$-*b*-**P2**$_{22}$ after 1 day aging at 25 °C. (c) Table of average width (W_n) of the 1D nanofibers from TEM images. For the analysis, **BCPs** were prepared after purification.

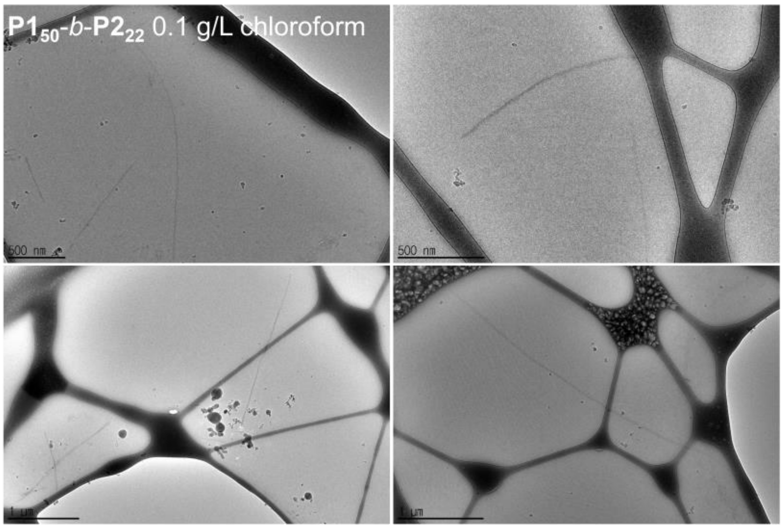

Fig. S19 Cryogenic TEM images of the 1D nanofibers from **P1**$_{50}$-*b*-**P2**$_{22}$ in 0.1 g/L chloroform, proving that the highly rigid 1D nanofibers already existed in the solution state.

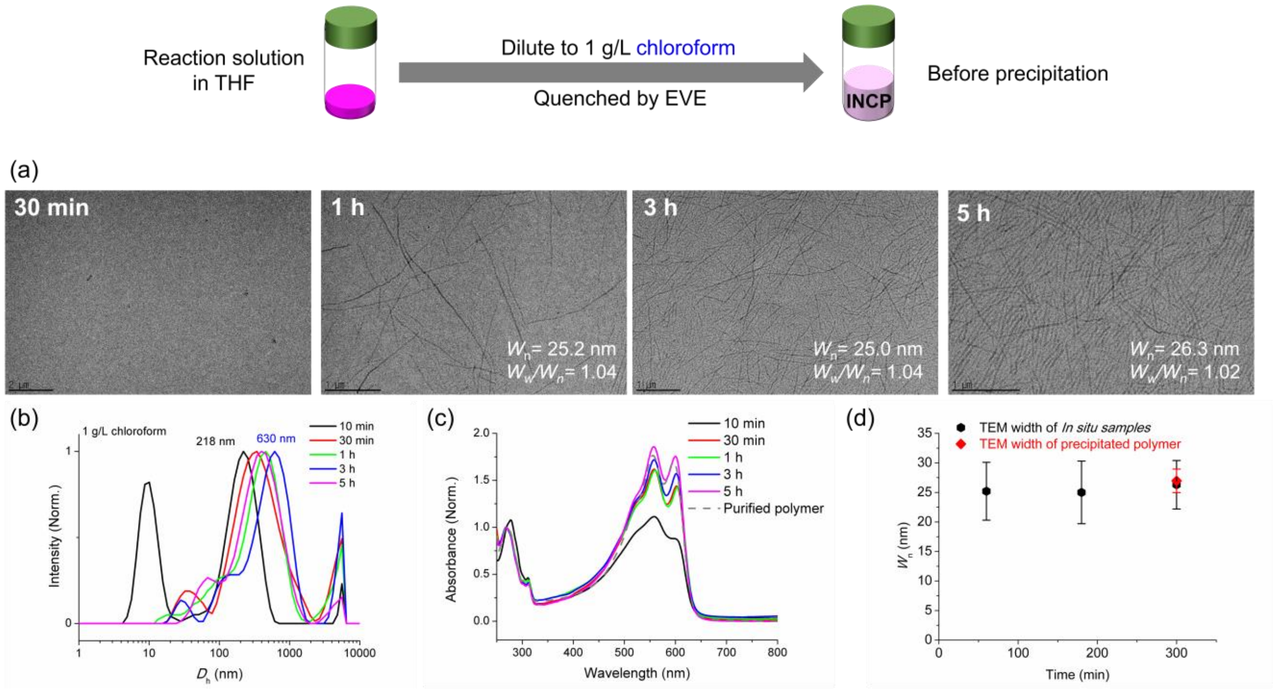

Fig. S20 The *in situ* TEM images of **P1$_{50}$**-*b*-**P2$_{66}$** at 1 g/L in chloroform by direct sampling during polymerization at 30 min (69% conv.), 1 hour, 3 hours, and 5 hours (99% conv.) (before precipitation, see **Fig. S7b**). The number in parentheses is the average width (W_n) and its width dispersity. These TEM images showed the same 1D nanofibers in chloroform regardless of precipitation process (Before precipitation: **Fig. S20** vs After precipitation: **Fig. 3f**). Changes of (b) DLS profiles, and (c) UV-vis absorbance spectra at the polymerization time points. (d) The plot of W_n of the 1D nanofibers from (a) TEM images**,** being compared with the W_n of precipitated **P1$_{50}$**-*b*-**P2$_{66}$** in **Fig. 3f** and **S18c**. Error bars in (d) indicate the standard deviation (σ).

(a)

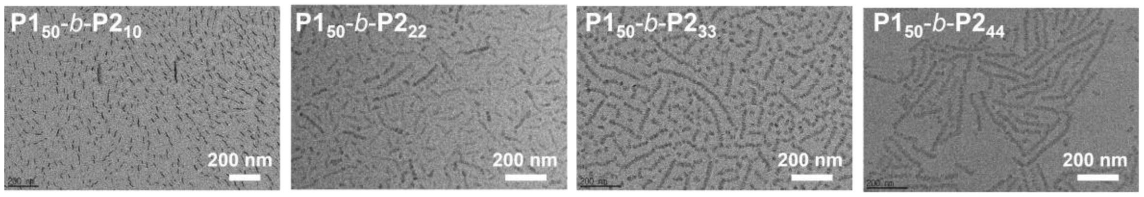

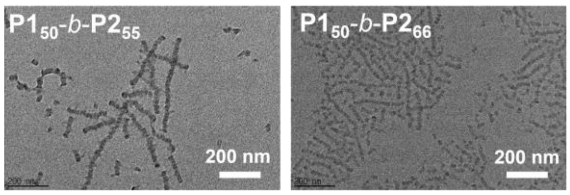

(b)

Entry	W_n (nm)	W_w/W_n	σ/W_n	σ (nm)
**P1$_{50}$**-*b*-**P2$_{22}$**	13.4	1.13	0.084	1.13
**P1$_{50}$**-*b*-**P2$_{33}$**	15.7	1.01	0.071	1.21
**P1$_{50}$**-*b*-**P2$_{44}$**	20.2	1.01	0.110	2.22
**P1$_{50}$**-*b*-**P2$_{55}$**	23.9	1.00	0.055	1.31
**P1$_{50}$**-*b*-**P2$_{66}$**	25.1	1.01	0.111	2.79

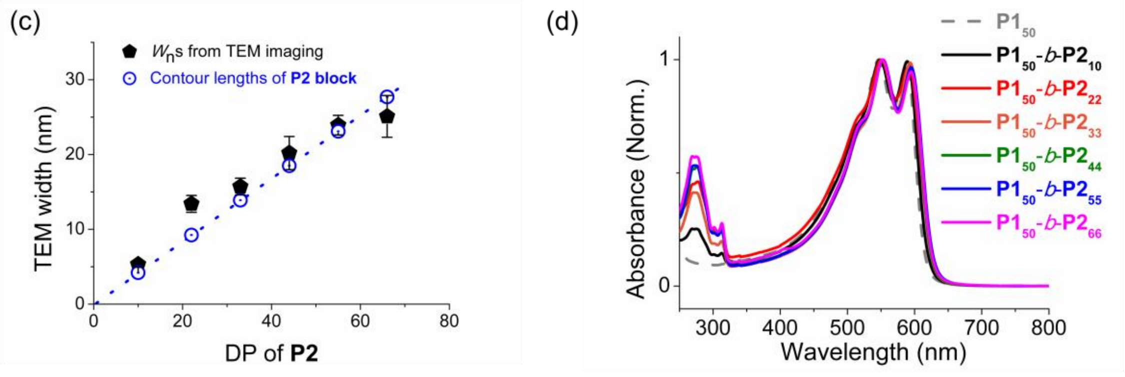

Fig. S21 (a) TEM images of **P1**$_{50}$-*b*-**P2**$_{n}$ (after purification) without aging in 1 g/L DCM. Lower solubility of the **BCPs** in DCM caused fast self-assembly, resulting in less-controlled length of the 1D nanofibers and large dispersities of D_h values (**Fig. S13d**). (b) Table of average width (W_n) of the 1D nanofibers from the TEM images, and (c) plots of the W_n from TEM images and contour lengths of the crystalline **P2** core (a dotted line) vs. the DP of **P2** in **BCPs**. (d) UV-vis absorbance spectra of **P1**$_{50}$ and **P1**$_{50}$-*b*-**P2**$_{n}$ after 1 day aging 0.05 g/L DCM. (Their initial UV-vis absorbance spectra were reported in **Fig. S13e**.)

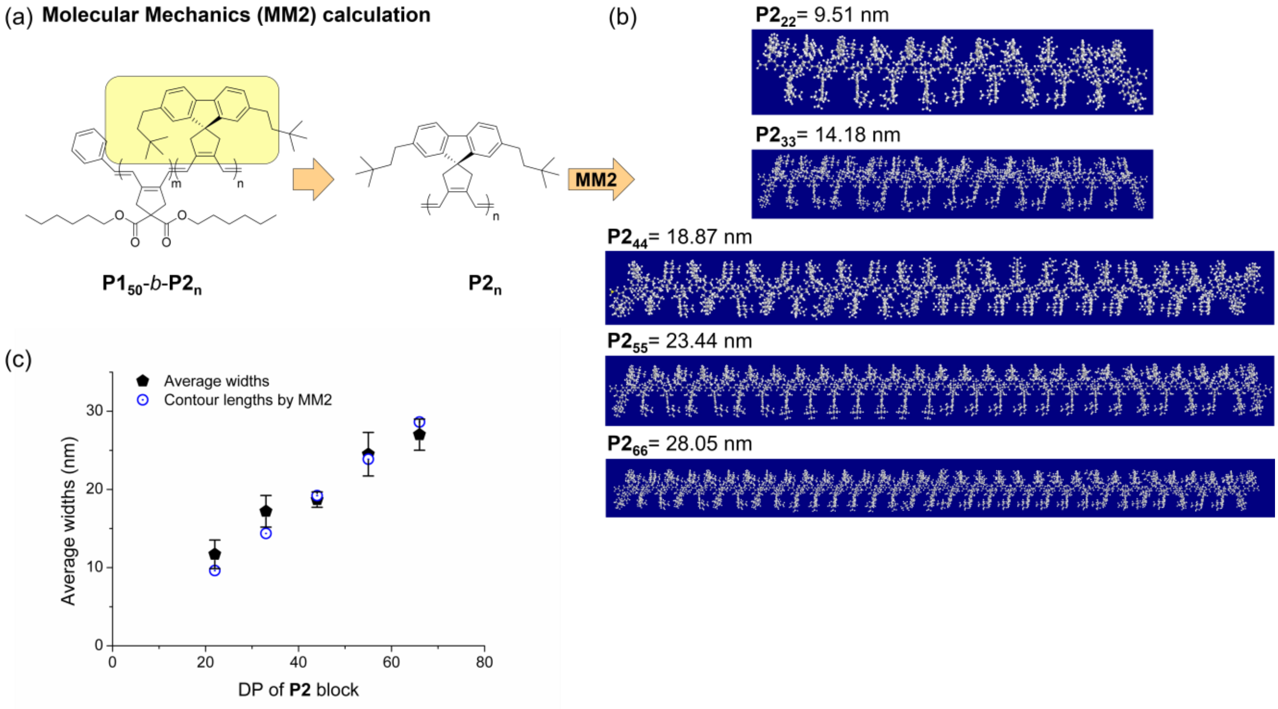

Fig. S22 Calculation of contour lengths of the **P2** core block of **P1**$_{50}$-*b*-**P2**$_{n}$ (**P2**$_{n}$). (a) The **P1**$_{50}$-*b*-**P2**$_{n}$ has been simplified to **P2**$_{n}$ (n = 22 ~ 66) for calculation. (b) Their rigid conformations with the contour lengths calculated by MM2 method. (c) Plots of the average width (W_n) of the 1D nanofibers from TEM images in chloroform and the calculated contour lengths vs. DP of **P2** block. Details in **Table S1**.

**P2 core block**	Chemical Formula	Calculated contour length (nm) by MM2 Calculation	Average widths from TEM images (nm)
**P2**$_{22}$	$C_{684}H_{840}$	9.51	11.7 (1.83)
**P2**$_{33}$	$C_{1025}H_{1258}$	14.18	17.2 (2.02)
**P2**$_{44}$	$C_{1366}H_{1676}$	18.87	18.7 (0.98)
**P2**$_{55}$	$C_{1707}H_{2094}$	23.44	24.5 (2.78)
**P2**$_{66}$	$C_{2048}H_{2512}$	28.05	27.0 (1.99)

Table S1 Table of chemical formula, contour lengths of **P2**$_{n}$ block calculated by MM2 method, and the W_n of **P1**$_{50}$-*b*-**P2**$_{n}$ (in **Fig. S18c**). The contour lengths of **P2**$_{n}$ from the calculation and average widths were well matched as we expected.[2,5]

(a)

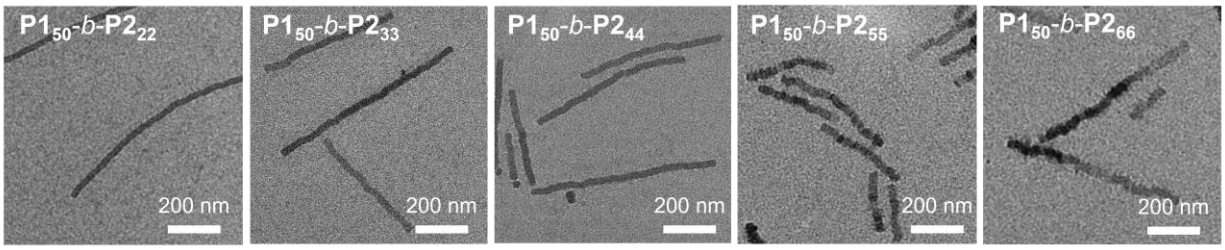

(b)

Entry	***Before staining***		***After staining***	
	W_n (nm)	σ	W_n (nm)	σ
**P1**$_{50}$-*b*-**P2**$_{22}$	11.7	1.8	21.0	2.2
**P1**$_{50}$-*b*-**P2**$_{33}$	17.2	2.0	26.3	2.1
**P1**$_{50}$-*b*-**P2**$_{44}$	18.7	1.0	28.8	2.3
**P1**$_{50}$-*b*-**P2**$_{55}$	24.5	2.8	33.9	3.4
**P1**$_{50}$-*b*-**P2**$_{66}$	27.0	2.0	35.3	5.2

Fig. S23 (a) High-magnified TEM images of the 1D nanofibers from **P1**$_{50}$-*b*-**P2**$_n$ in 0.05 g/L chloroform after RuO_4 staining. (b) Table of average width (W_n) of the 1D nanofibers from the stained TEM images, compared with initial W_n of the 1D nanofibers. Further staining with RuO_4 vapor made the **P1** corona block dense and observable by TEM, and the width difference before and after staining was constant regardless of the DP of the **P2** block because all **BCPs** have the same DP of the **P1** shell block.

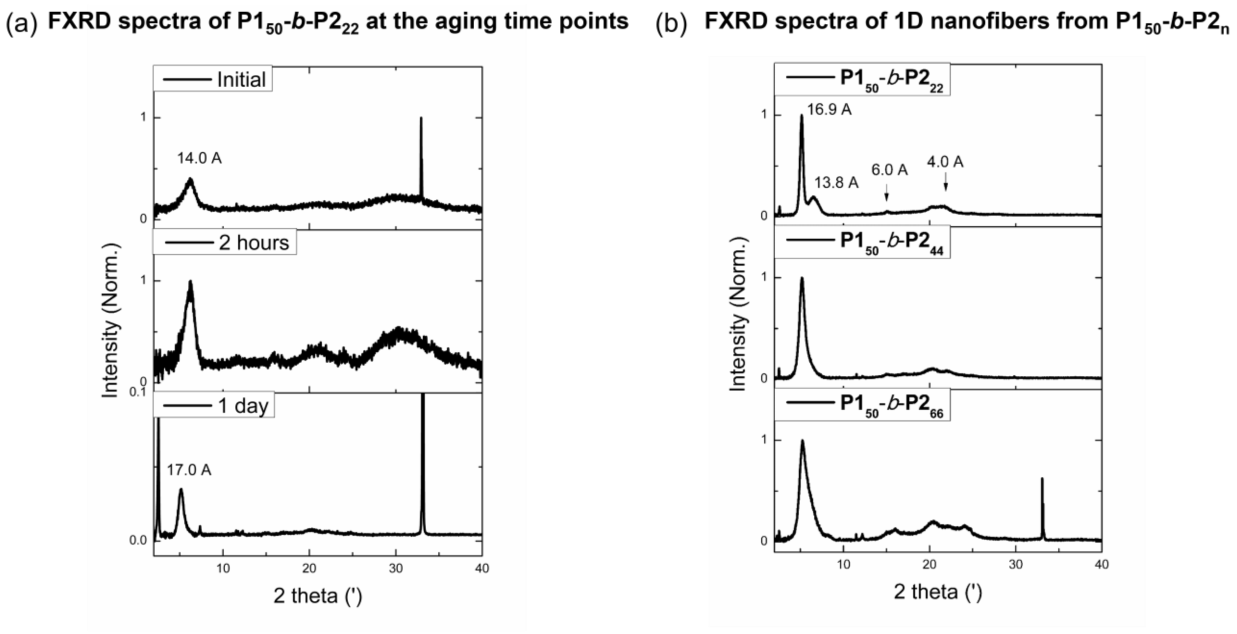

Fig. S24 (a) Film-XRD spectra of the 1D nanofibers from **P1**$_{50}$-*b*-**P2**$_{n}$ at the aging time points, and (b) the spectra of **P1**$_{50}$-*b*-**P2**$_{22}$, **P1**$_{50}$-*b*-**P2**$_{44}$, and **P1**$_{50}$-*b*-**P2**$_{66}$. The signal of **P1** shell block decreased in the results of longer **BCPs** and longer 1D nanofibers, because the **P1** block only formed amorphous structure. All samples were prepared by drop-casting of polymer solutions in 10 g/L chloroform on SiO_2 surface.

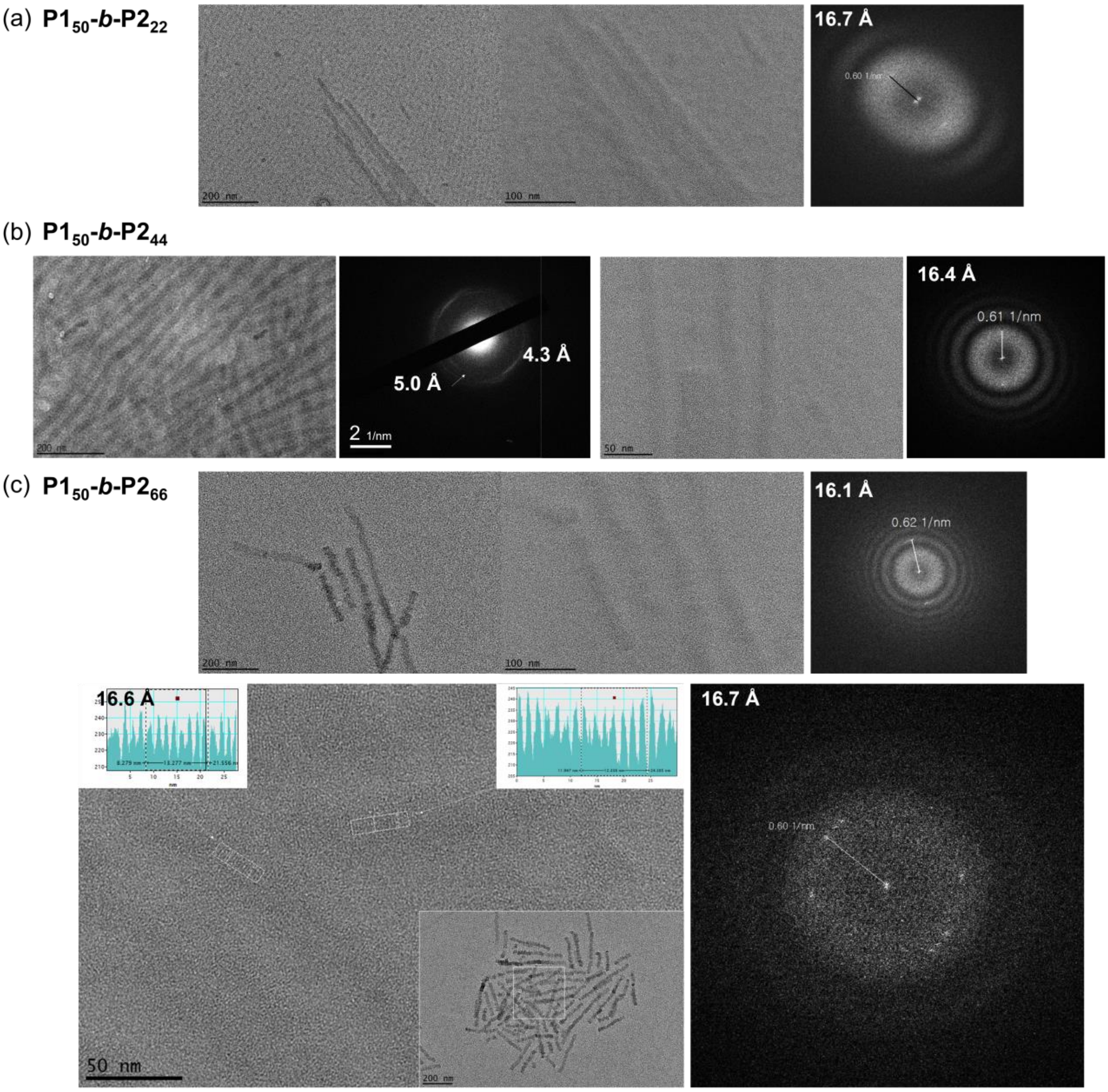

Fig. S25 HR-TEM images of the 1D nanofibers from (a) **P1**$_{50}$-*b*-**P2**$_{22}$, (b) **P1**$_{50}$-*b*-**P2**$_{44}$, and (c) **P1**$_{50}$-*b*-**P2**$_{66}$ with their FFT patterns showing *d*-spacing of 16.1–16.7 Å. An SAED image from **P1**$_{50}$-*b*-**P2**$_{44}$ was identical with that from **P1**$_{50}$-*b*-**P2**$_{22}$ in **Fig. 4c**.

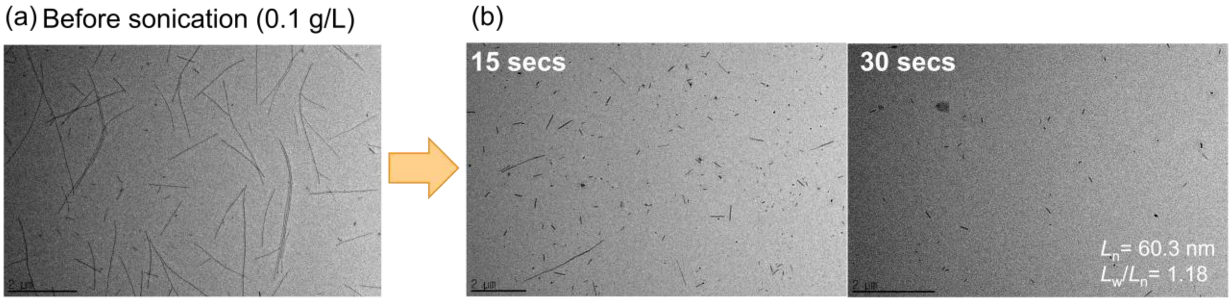

Fig. S26 TEM images of (a) **P1**$_{50}$-*b*-**P2**$_{22}$ in aged 0.1 g/L chloroform, and (b) sonicated solution for total on-time 15 secs and 30 secs using the 20 kHz VCX-500 series sonication probe with an extender tip (1.25 cm, tip diameter). The on-time 15 sec sonication was not enough to form the uniform 1D seed structures, and on-time 30 secs was required to get uniform 1D seed structures with L_n= 60.3 nm (L_w/L_n= 1.18).

* The sonication condition is 11.8 W/cm^2 (20% amplitude setting on the sonicator) with a pulse sequence of 2 secs on, 8 secs off at 0 °C. The numbers in parentheses were the L_n and its dispersity. We calibrated the sonicator according to literature procedures. [6]

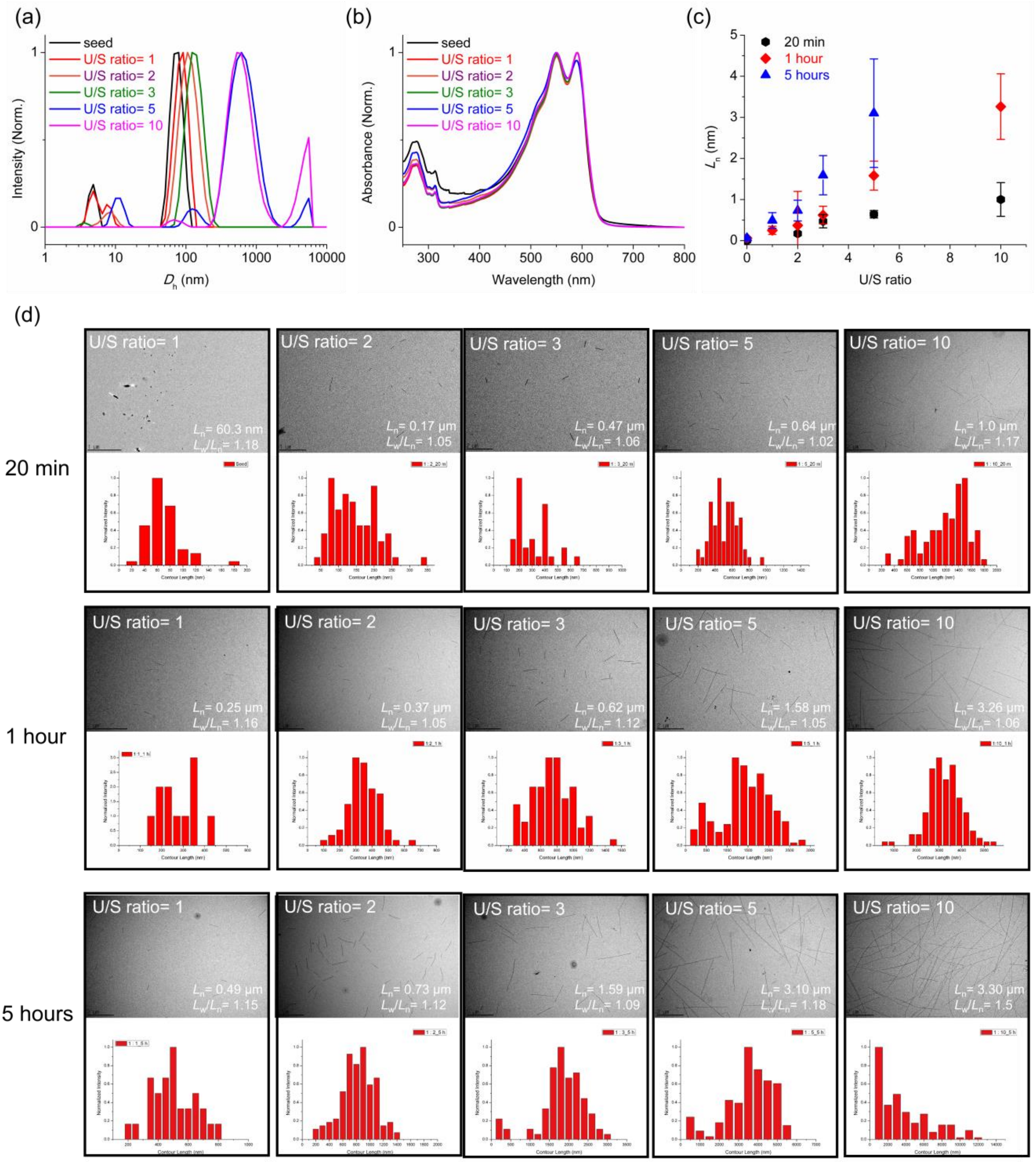

Fig. S27 The CDSA of **P1**$_{50}$-*b*-**P2**$_{22}$ in chloroform. (a) DLS profiles, (b) UV-vis absorbance spectra, and (c) plots of the average length (L_n) vs. U/S ratios after 20 min, 1 hour, and 5 hours aging at 25 °C. Error bars indicate the standard deviation (σ). (d) TEM images and contour length histograms of 1D nanofibers prepared by CDSA process using unimer-to-seed ratio (U/S ratio) from 1 to 2, 3, 5, and 10 with seed micelles from **Fig. S26** at 25 °C. The number in parentheses is "the average L_n and its length dispersity."

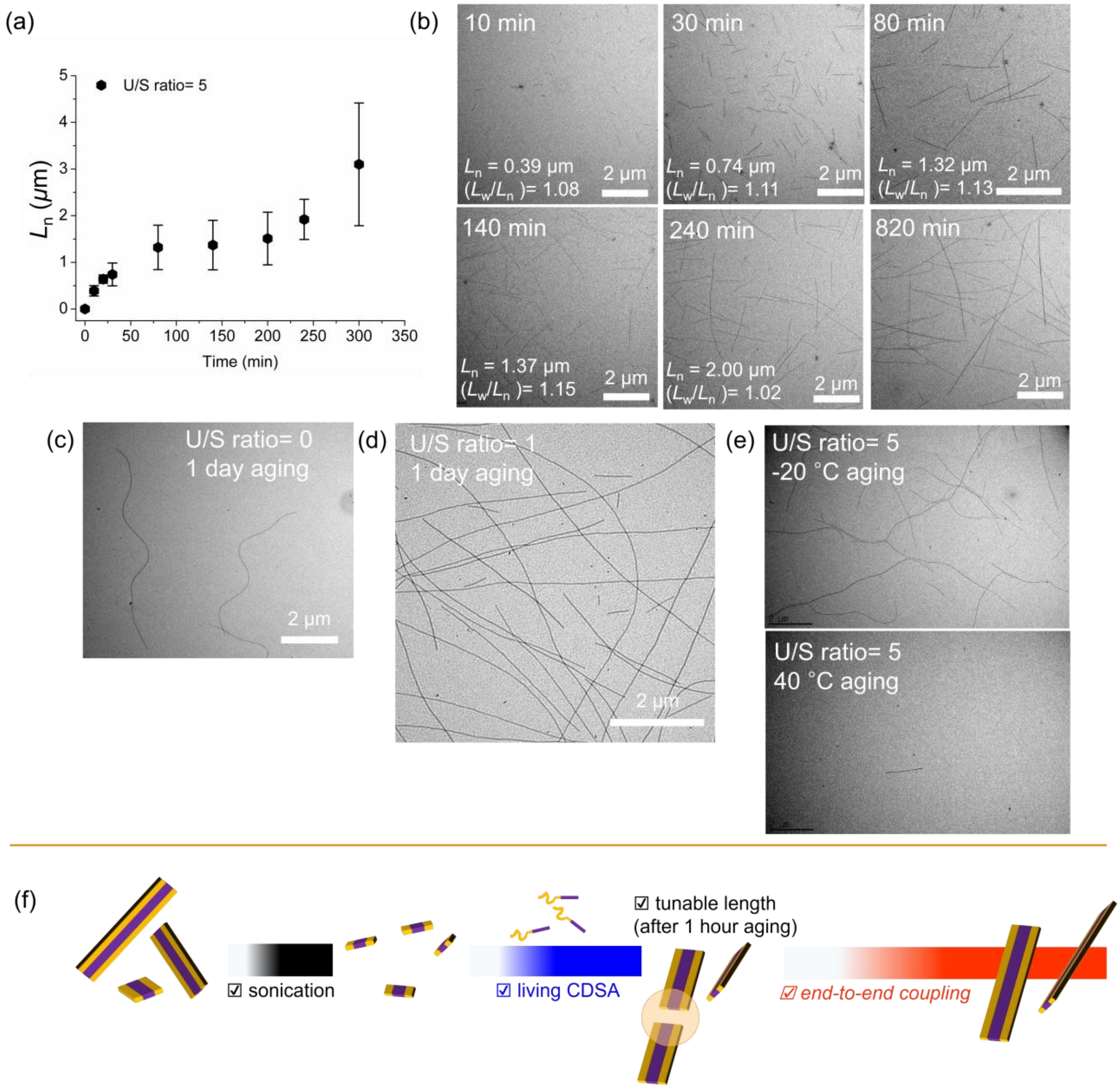

Fig. S28 (a) A plot of L_n values vs. time for assembly monitored for 1 day with U/S ratio = 5) and (b) TEM images in the growth kinetic studies. In the initial chloroform solution, a $\mathbf{P1}_{50}$-*b*-$\mathbf{P2}_{22}$ followed the typical seeded growth where only seed-to-unimer assembly occurred. In 100 min–200 min time range, the growth seemed to be stagnated, as if seeded growth was complete; however, the length increased again, indicating that end-to-end coupling (as the second growth) occurred. Presumably, the dynamic exchange from the end of the 1D nanofibers to unimers promotes the end-to-end coupling process. (c) TEM images of very long 1D nanofibers without added unimers and (d) with U/S ratio = 1 to seed-micelles. (e) TEM images of the 1D nanofibers by seeded growth using U/S ratio 5 at -20 °C and 40 °C. Lowering the aging temperature (temp.) further promoted all assemblies (view of thermodynamic perspective), resulting in longer 1D nanofibers after 1 hour (a living CDSA failed). Contrary to this, at higher temp (40 °C), unimers cannot assemble to the seed due to higher kinetic energy. (f) Schematic illustration of the growing process of 1D nanofibers from $\mathbf{P1}_{50}$-*b*-$\mathbf{P2}_{22}$ in chloroform.

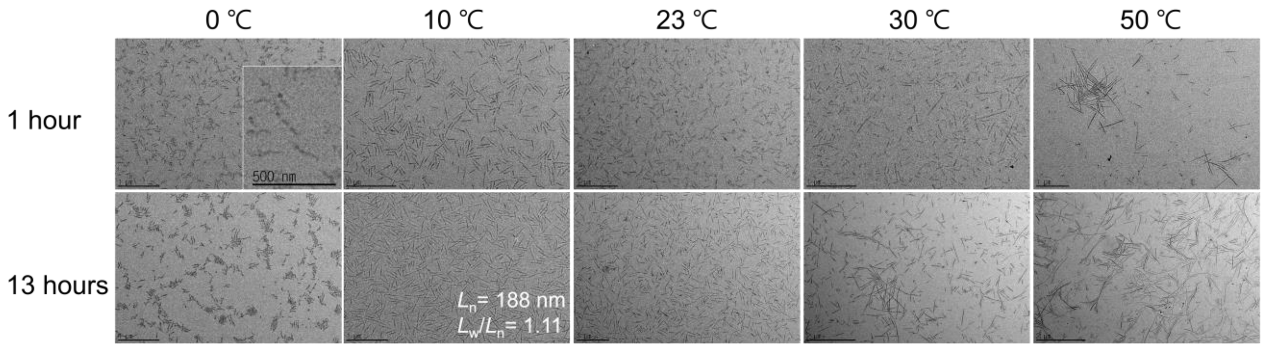

Fig. S29 Low-magnified TEM images of 1D nanofibers from **P1**$_{50}$-*b*-**P2**$_{22}$ by seeded growth of seed-micelles (L_n = 62.1 nm, L_w/L_n = 1.13) in 0.1 g/L DCM by adding unimers (in 10 g/L chloroform) with U/S ratio of 5 at various aging temperatures from 0 to 50 °C.

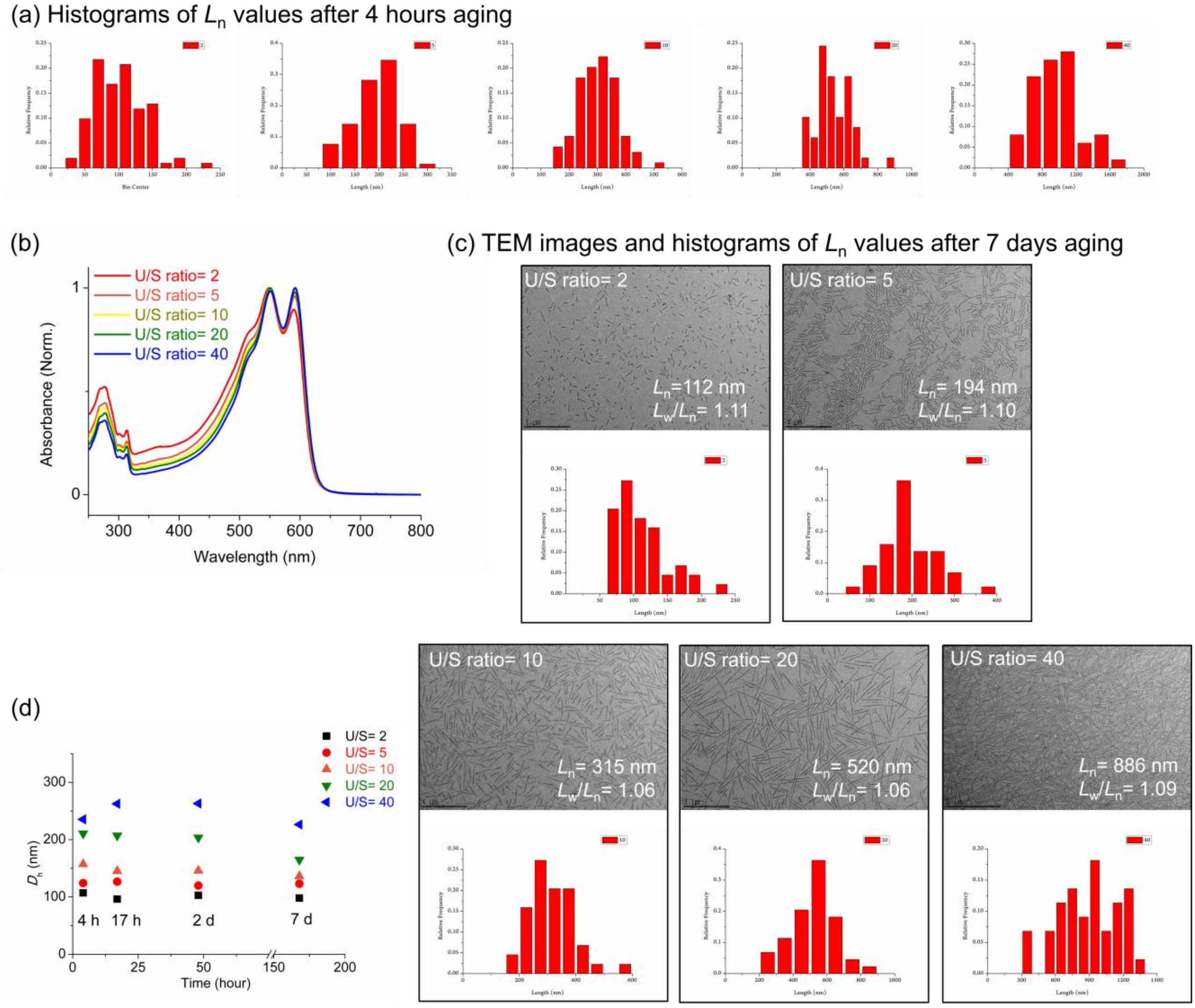

Fig. S30 The CDSA of P1$_{50}$-*b*-P2$_{22}$ in DCM. (a) Contour length histograms, and (b) UV-vis absorbance spectra of 1D nanofibers from **P1$_{50}$**-*b*-**P2$_{22}$** by the same seeded growth condition in **Fig. 5** with various U/S ratios from 2 to 40 after 4 hours aging at 10 °C. (c) TEM images and histograms of L_n after 7 days at 10 °C aging showing the long-term stabilities of those 1D nanofibers. (d) Plots of D_h with various U/S ratios vs. aging time. Constant D_h values for 7 days also supported high stability.

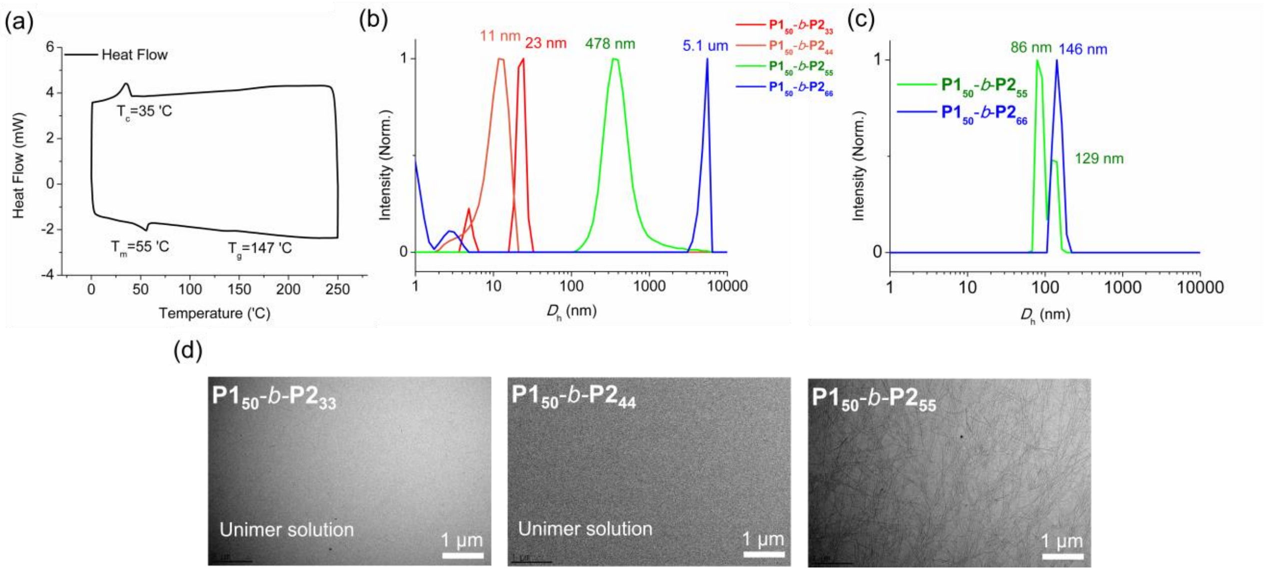

Fig. S31 (a) A DSC curve of 1D nanofibers from **P1**$_{50}$-*b*-**P2**$_{22}$ prepared by seeded growth method, representing the melting temperature (T_m) at 55 °C with 10.0 °C/min heating rate. A proper heating temperature for preparing unimer solutions was set as 60 °C. (b) DLS profiles of longer **BCPs** (**P1**$_{50}$-*b*-**P2**$_{33\text{-}66}$) in 1 g/L chloroform after heating at 60 °C for 30 min. D_h values of unimers were obtained in solutions of **P1**$_{50}$-*b*-**P2**$_{33}$ and **P1**$_{50}$-*b*-**P2**$_{44}$. Those of nanoparticles were obtained in solutions of **P1**$_{50}$-*b*-**P2**$_{55}$ and **P1**$_{50}$-*b*-**P2**$_{66}$. (c) DLS profiles after 30 min heating at a higher temp. (80 °C). Due to the high crystallinity of longer **BCPs**, 1D nanofibers were shown even after heating in DLS profiles and (d) TEM images of **P1**$_{50}$-*b*-**P2**$_{55}$.

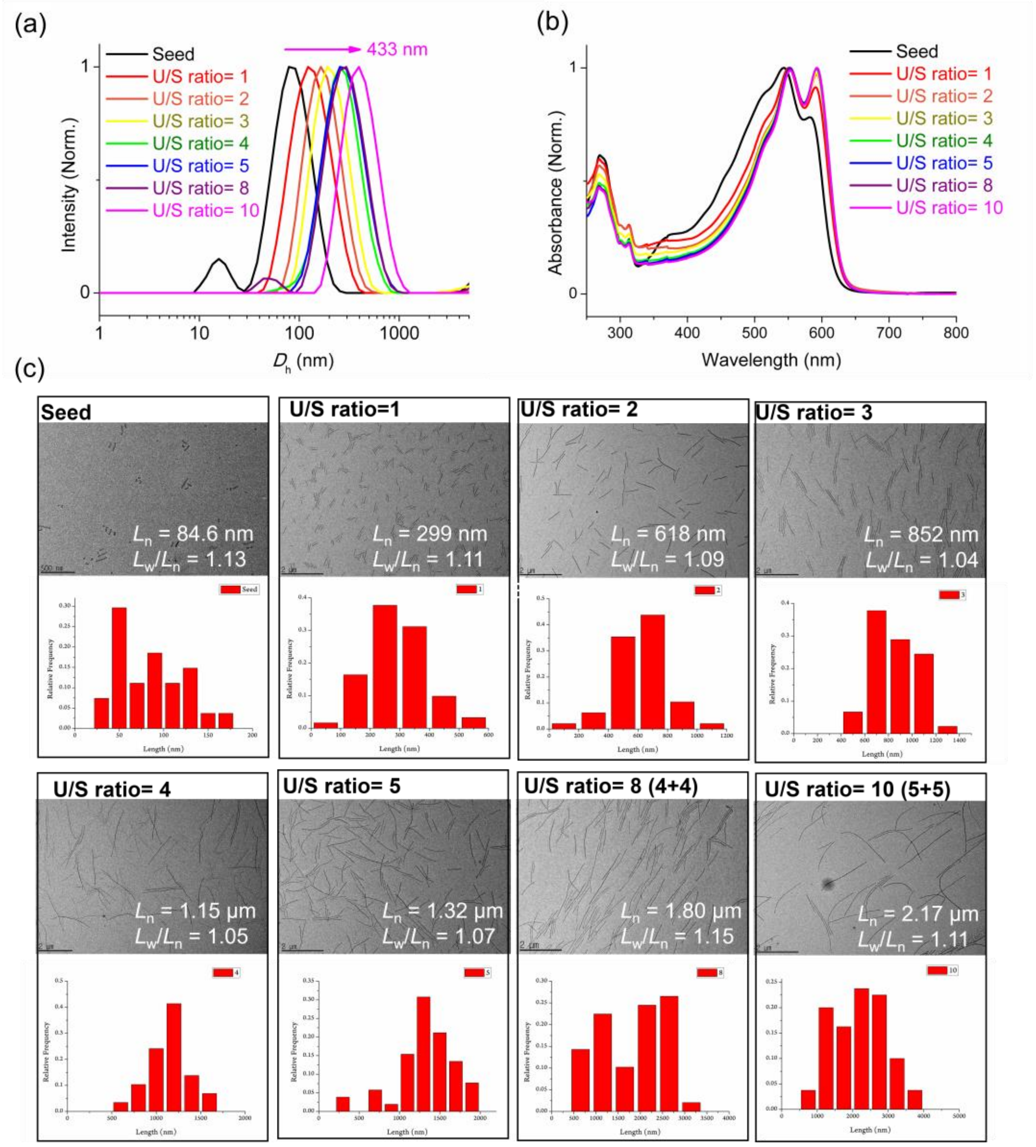

Fig. S32 The CDSA of $\mathbf{P1_{50}}$-*b*-$\mathbf{P2_{33}}$ in chloroform. (a) DLS profiles, (b) UV-vis absorbance spectra, (c) TEM images, and contour length histograms of 1D nanofibers from $\mathbf{P1_{50}}$-*b*-$\mathbf{P2_{33}}$ prepared by CDSA process from seed-micelles (L_n = 84.6 nm, L_w/L_n = 1.13) in 0.1 g/L chloroform after addition of unimers (in 1 g/L chloroform) with U/S ratios of 1, 2, 3, 4, 5, 8, and 10 (1 hour aging).

In detail, we first failed to get the narrow L_w/L_n when the unimer was added at once (U/S ratio > 5) because the growth process was quite fast due to the high crystallinity. To solve this limitation, we injected the unimers in two portions with 30 min interval and succeeded in living seeded growth up to L_n = 2.2 μm with L_w/L_n = 1.11 (5 + 5 eq). D_h values of the1D nanofibers gradually increased, and L_ns of them linearly increased according to the U/S ratios. For the longer L_n than the theoretically predicted one, we speculate that when adding a heated unimer solution to seed solutions, an increase in temperature and a decrease in concentration can cause the partial dissolution of the seeds and formation of longer 1D nanofibers.

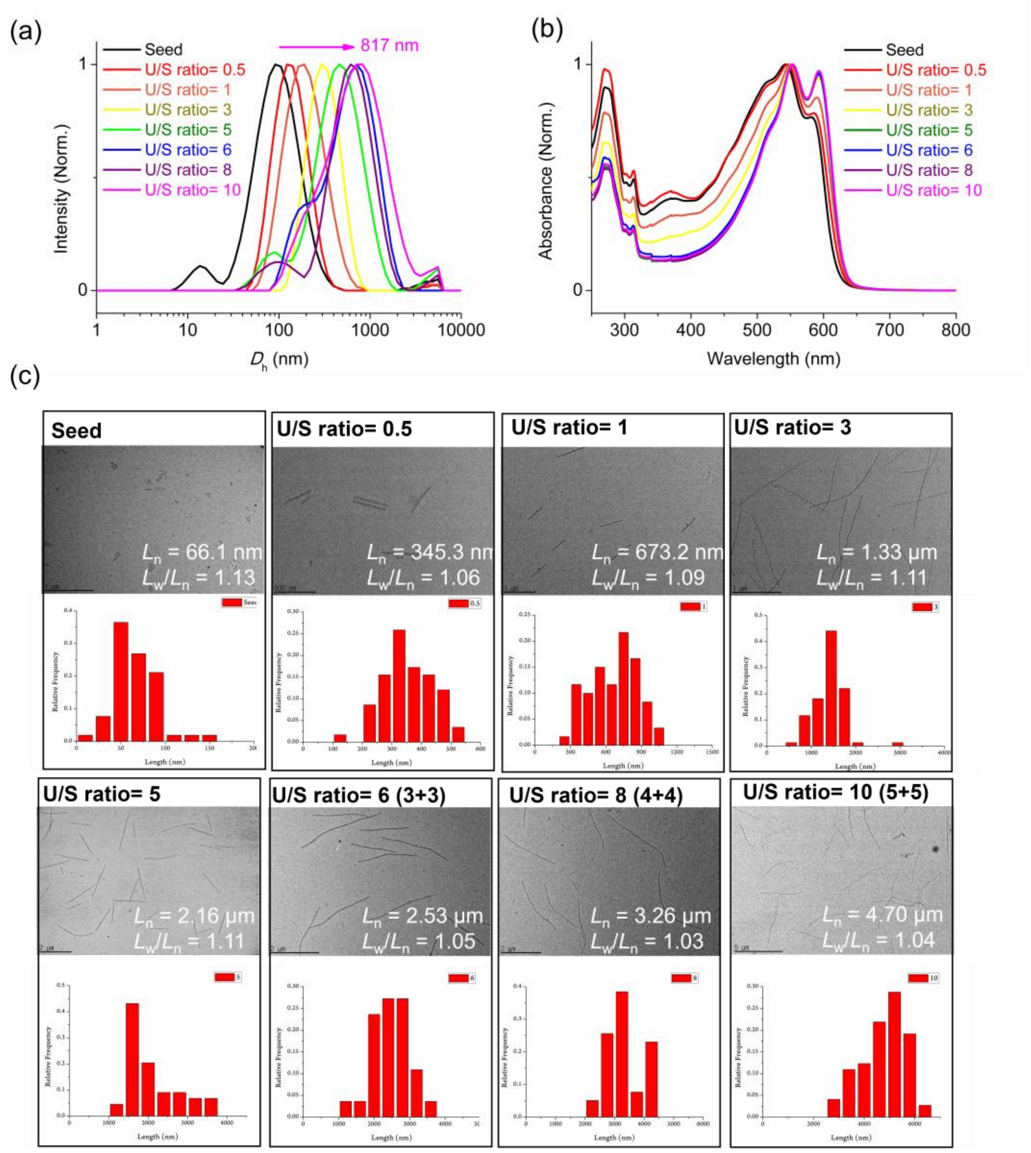

Fig. S33 The CDSA of P1$_{50}$-*b*-P2$_{44}$ in chloroform. (a) DLS profiles, (b) UV-vis absorbance spectra, (c) TEM images, and contour length histograms of 1D nanofibers from **P1$_{50}$**-*b*-**P2$_{44}$** prepared by CDSA process from seed-micelles (L_n = 66.1 nm, L_w/L_n = 1.13) in 0.1 g/L chloroform after addition of unimers (in 1 g/L chloroform) with U/S ratios of 0.5, 1, 3, 5, 6, 8, and 10 (1 hour aging).

In detail, we first failed to get the narrow L_w/L_n when the unimer was added at once (U/S ratio > 5) because the growth process was quite fast due to the high crystallinity. To solve this limitation, we injected the unimers in two portions with 30 min interval and succeeded in living seeded growth up to L_n = 4.7 μm with L_w/L_n = 1.04 (5 + 5 eq). D_h values of the1D nanofibers gradually increased, and L_ns of them linearly increased according to the U/S ratios. For the longer L_n than the theoretically predicted one, we speculate that when adding a heated unimer solution to seed solutions, an increase in temperature and a decrease in concentration can cause the partial dissolution of the seeds and formation of longer 1D nanofibers.

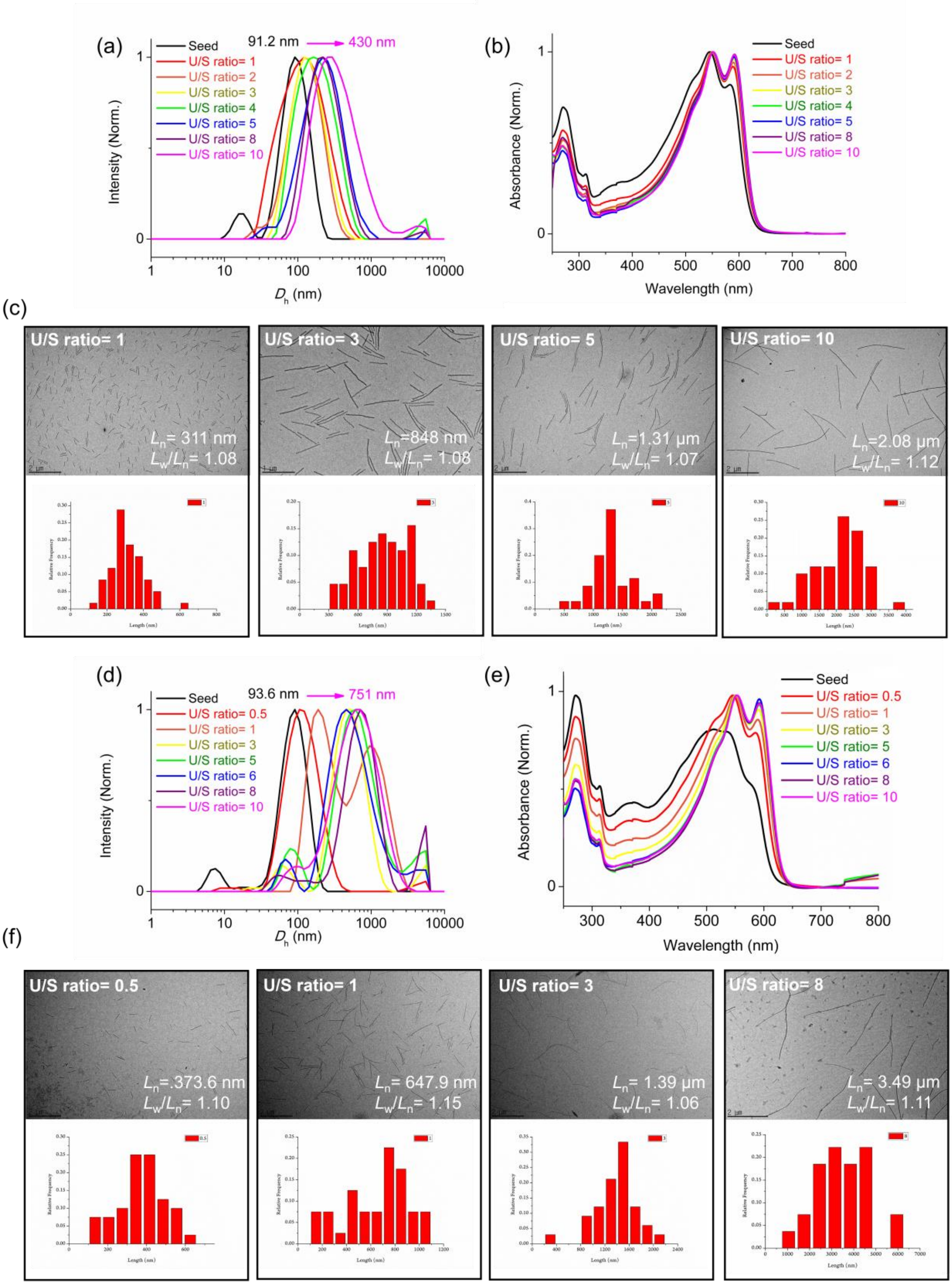

Fig. S34 (a) DLS profiles, (b) UV-vis absorbance spectra, (c) TEM images, and their contour length histograms of the 1D nanofibers from **P1**$_{50}$-*b*-**P2**$_{33}$ after 1 day aging. (d) DLS profiles, (e) UV-vis absorbance spectra, (f) TEM images, and their contour length histograms of the 1D nanofibers from **P1**$_{50}$-*b*-**P2**$_{44}$ after 1 day aging. All results indicate that the end-to-end coupling between 1D nanofibers did not happen.

(a)

Entry	[**M1**]:[**M2**]:[cat]	Time for **M2**	Conv. (%) [a]	Yield (%)	M_n(kDa)[b]	*Đ*[b]
7	100:55:1	10 h	> 99%, > 99%	91.3	2600	1.31
8	100:66:1	12 h	> 99%, > 99%	87.6	3800	1.17

(b)

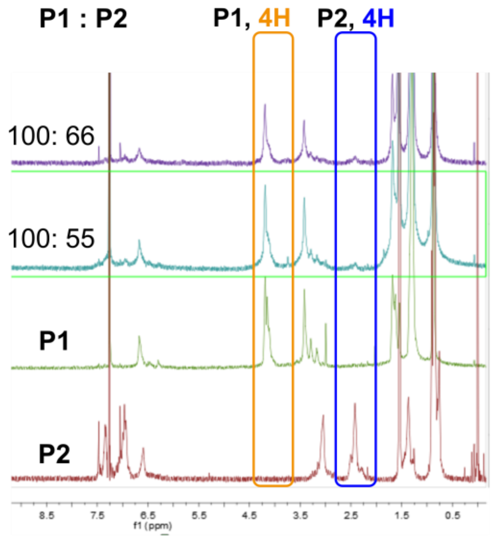

(c)

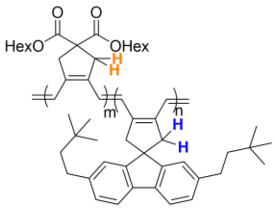

(d)

$CDCl_3$	**P2** block intensity (**4H**)
	Initial%
**P1**$_{100}$-*b*-**P2**$_{55}$	6
**P1**$_{100}$-*b*-**P2**$_{66}$	17

Fig. S35 (a) A table of the living cyclopolymerization of **P1**$_{100}$-*b*-**P2**$_{n}$. (b) ^{1}H NMR spectra of **P1**$_{100}$-*b*-**P2**$_{n}$ in chloroform-*d* at 20 °C. (c) Protons corresponding to the two boxes on the spectra were represented on the polymer structures. (d) The relative **P2**% (see **Fig. S2**). ([a]Calculated by ^{1}H NMR analysis. [b]Determinted by AF4 fractograms in chloroform using 0.205 as a *dn/dc* value).

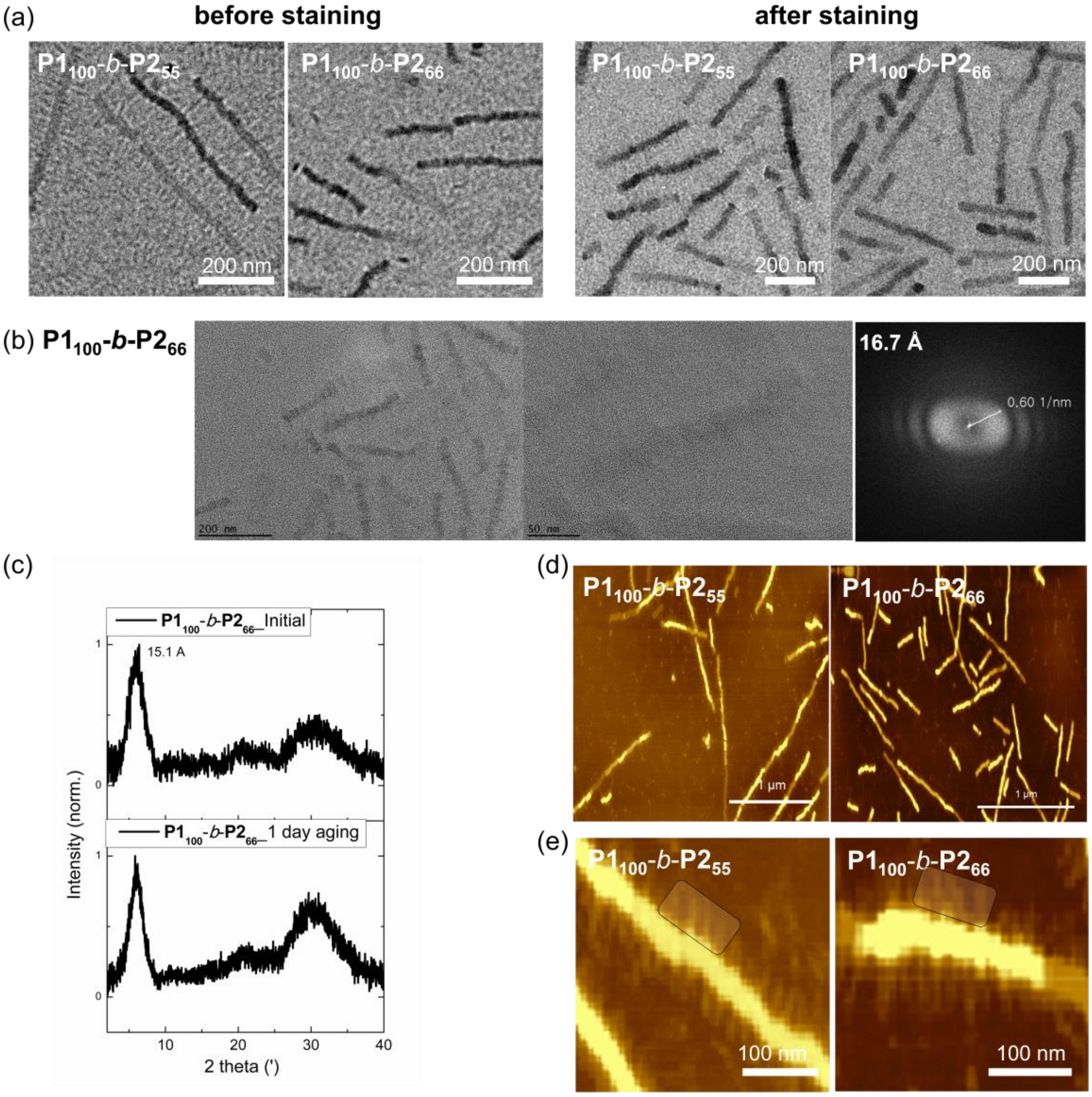

(f)

Entry	*Before staining*		*After staining*	
	W_n (nm)	σ (nm)	W_n (nm)	σ (nm)
**P1**$_{100}$-*b*-**P2**$_{55}$	25.8	3.6	34.2	3.4
**P1**$_{100}$-*b*-**P2**$_{66}$	31.6	4.5	37.3	5.3

(g)

Entry	H_n (nm)	H_w/H_n	$σ/H_n$	σ (nm)
**P1**$_{100}$-*b*-**P2**$_{55}$	9.5	1.04	0.2	1.9
**P1**$_{100}$-*b*-**P2**$_{66}$	7.4	1.03	0.17	1.3

Fig. S36 (a) High-magnified TEM images of 1D nanofibers from purified **P1**$_{100}$-*b*-**P2**$_n$ (n= 55, 66) in 0.05 g/L chloroform before and after RuO_4 staining. (b) An HR-TEM image of **P1**$_{100}$-*b*-**P2**$_{66}$ and its FFT pattern showing *d*-spacing of 16.7 Å. This FFT pattern is identical with that of **P1**$_{50}$-*b*-**P2**$_{66}$. (c) FXRD spectra of 1D nanofibers from the **P1**$_{100}$-*b*-**P2**$_{66}$ before and after aging, showing a similar diffraction with the main *d*-spacing of the crystalline **P2** block. We assumed that broad peaks in the 20−40° range result from the longer corona **P1**$_{100}$ block. (d) AFM height images of rigid 1D nanofibers from the new **BCPs** and (e) High-magnified AFM height images of them representing longer outer **P1** block than **P1**$_{50}$-*b*-**P2**$_n$ in **Fig. 2g** and **2h**. Tables of (f) average width (W_n) before and after staining and (g) average height (H_n) of the 1D nanofibers. The W_n values were well-matched with the trend of widths of **P1**$_{50}$-*b*-**P2**$_n$ correlating with DP of **P2**.

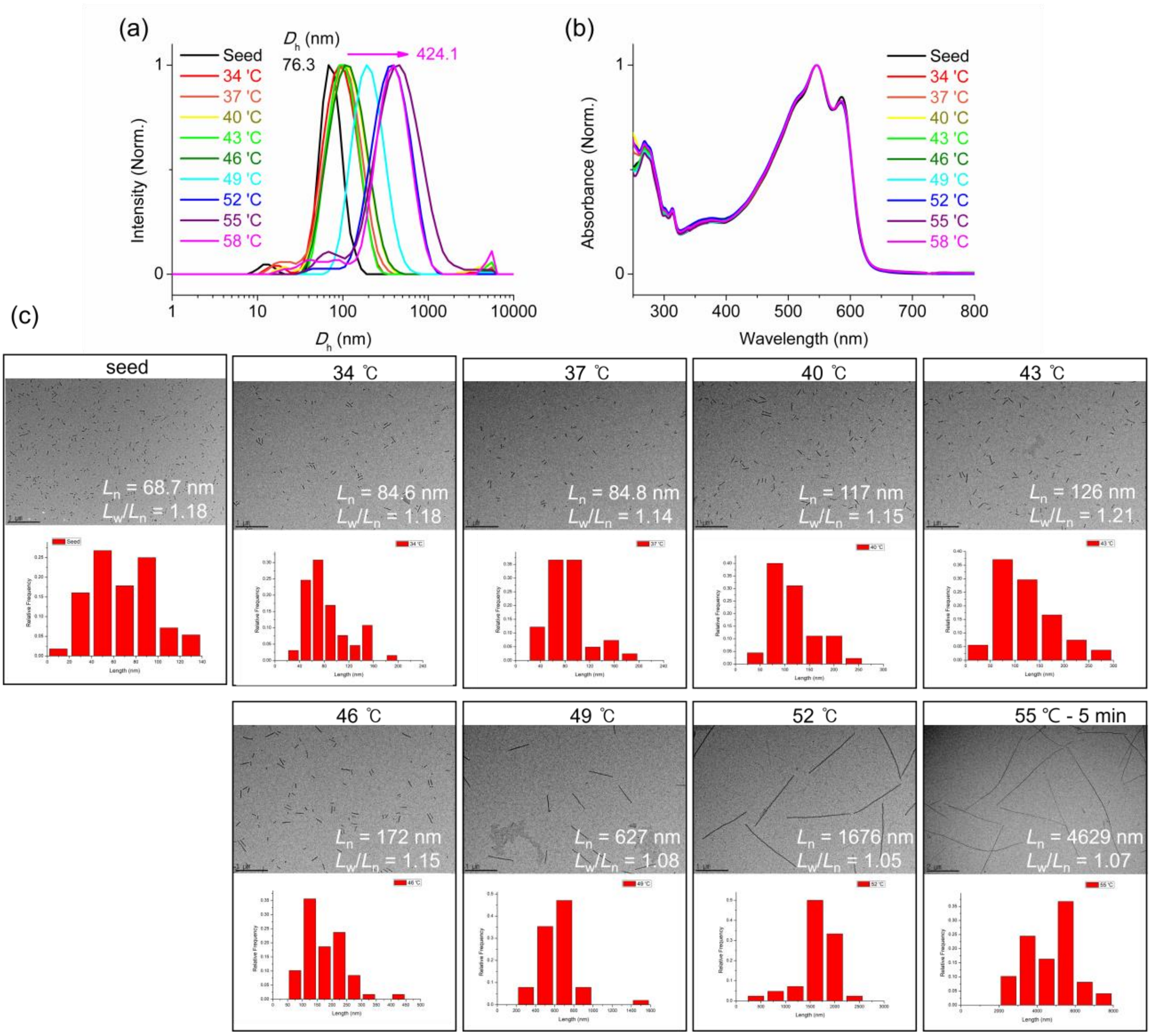

Fig. S37 The CDSA of P1$_{100}$-*b*-P2$_{55}$ in chloroform via the self-seeding method. (a) DLS profiles, (b) UV-vis absorbance spectra, (c) TEM images, and contour length histograms of 1D nanofibers from **P1$_{100}$**-*b*-**P2$_{55}$** prepared by CDSA process from seed-micelles (L_n = 68.7 nm, L_w/L_n = 1.18, sonication on time 30 secs) in 0.1 g/L chloroform. We applied the self-seeding method to control the length of their 1D nanofibers because we failed to prepare the unimer solutions of **P1$_{50}$**-*b*-**P2$_n$** and **P1$_{100}$**-*b*-**P2$_n$** due to their high crystallinity. Also, as **Fig. S36a, b** showed, the low-height part of 1D nanofibers of **P1$_{100}$**-*b*-**P2$_n$** formed low-height seeds after sonication. This height deviation can be solved by self-seeding method, where seeds with low-height were preferentially melted due to low-crystallinity.

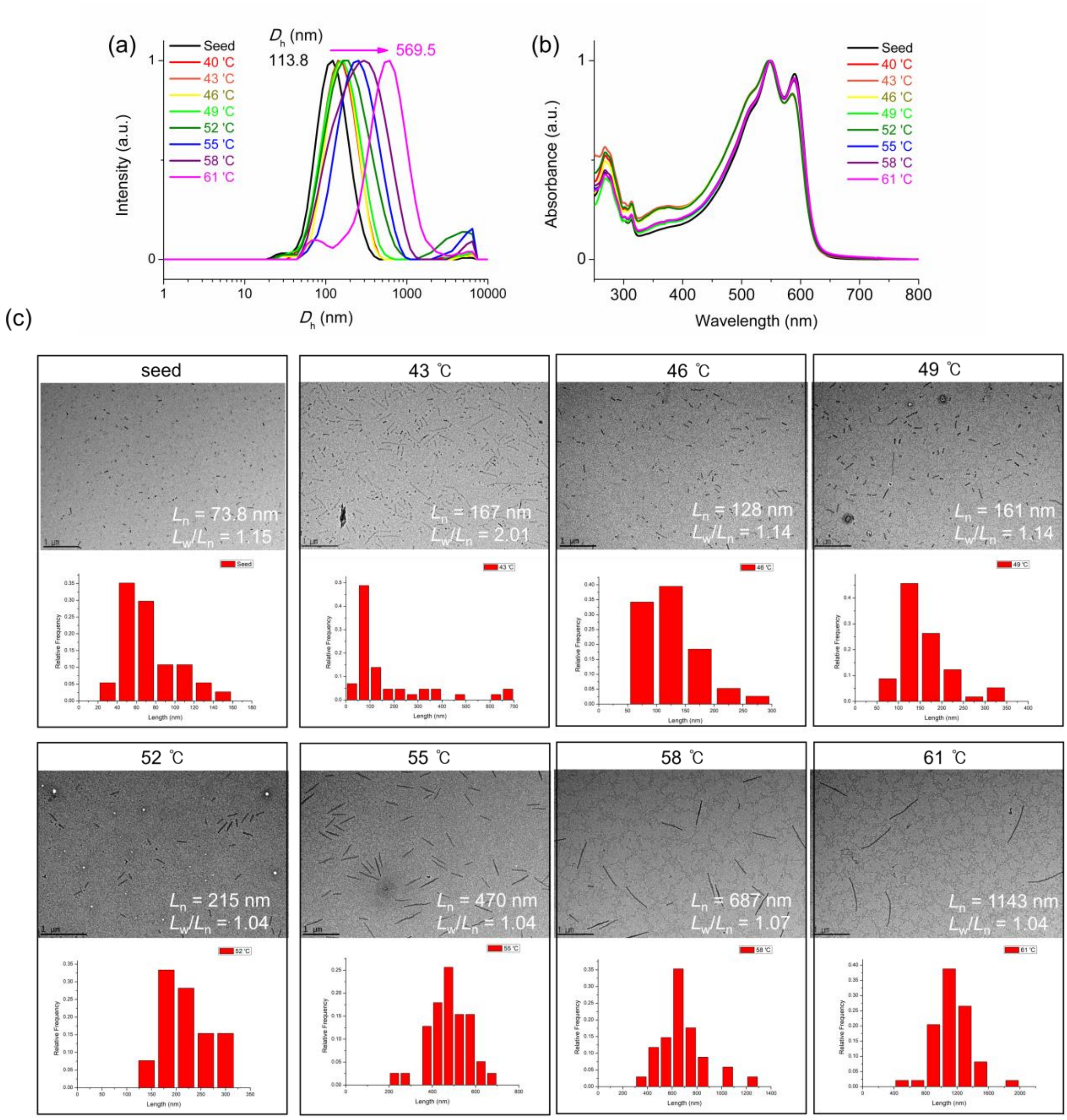

Fig. S38 The CDSA of P1$_{100}$-*b*-P2$_{66}$ in chloroform via self-seeding method (a) DLS profiles, (b) UV-vis absorbance spectra, (c) TEM images, and contour length histograms of 1D nanofibers from **P1$_{100}$**-*b*-**P2$_{66}$** prepared by CDSA process from seed-micelles (L_n = 73.8 nm, L_w/L_n = 1.15, sonication on time 30 secs) in 0.1 g/L chloroform.

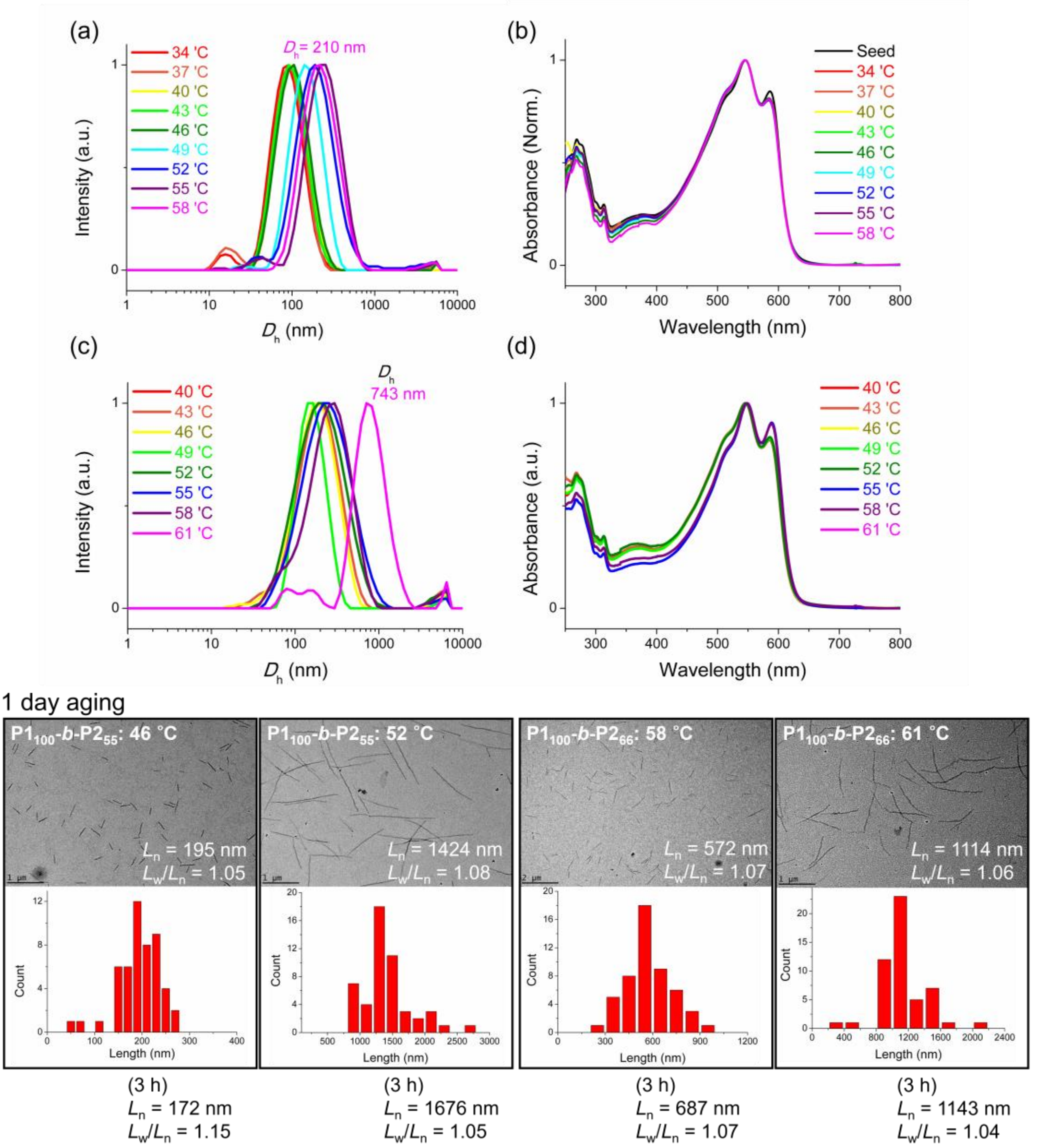

Fig. S39 (a) DLS profiles, (b) UV-vis absorbance spectra of the 1D nanofibers from **$P1_{100}$-*b*-$P2_{55}$**, and (c) DLS profiles, (d) UV-vis absorbance spectra of the 1D nanofibers from **$P1_{100}$-*b*-$P2_{66}$** after 12 hours aging. (e) TEM images, and contour length histograms of both 1D nanofibers after 1 day aging.

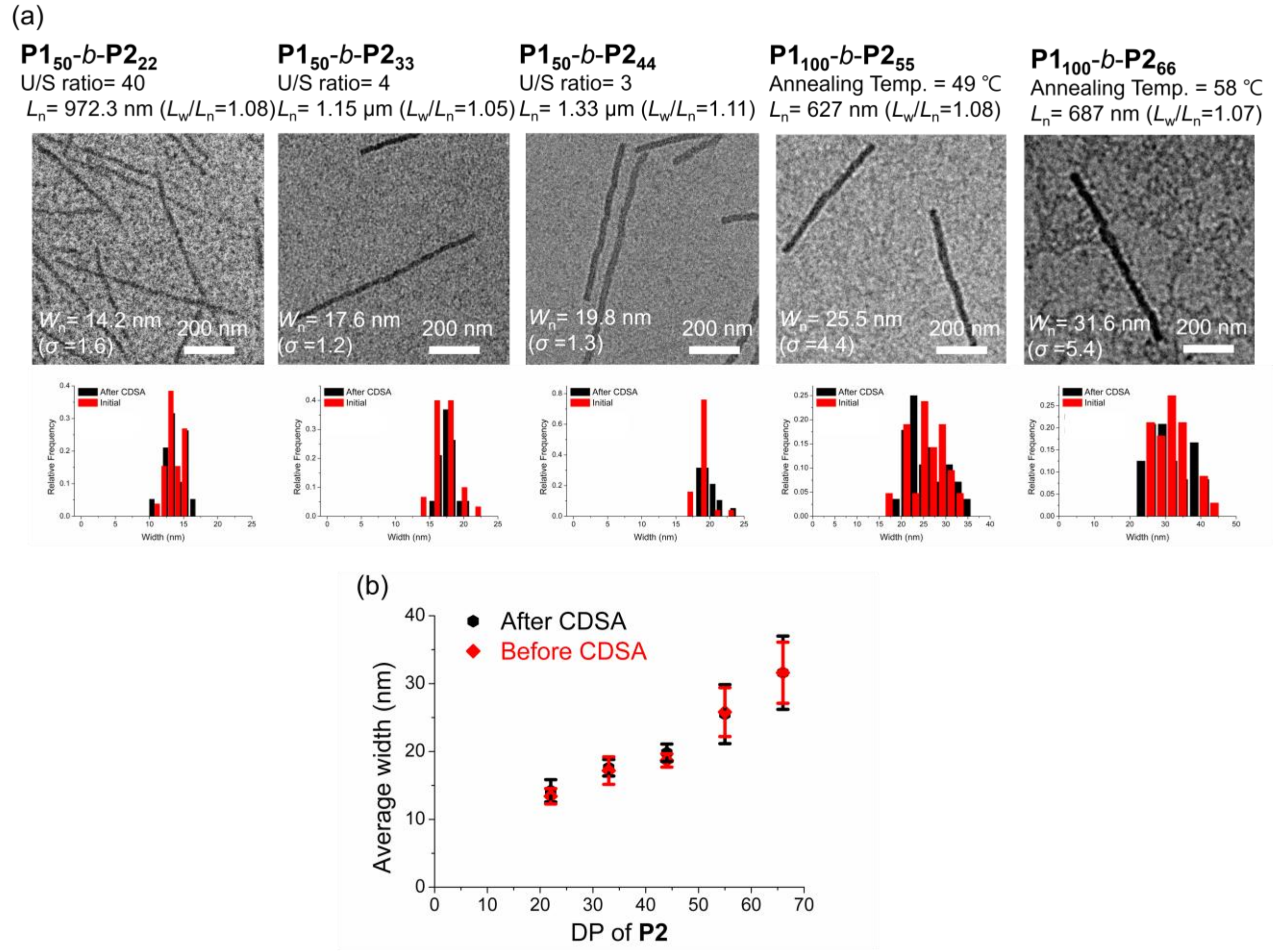

Fig. S40 (a) High-magnified TEM images, and contour width histograms of 1D nanofibers from **P1**$_{50}$-*b*-**P2**$_{n}$ and **P1**$_{100}$-*b*-**P2**$_{n}$ prepared by living CDSA. (b) Plots of their W_n before and after CDSA vs. DP of **P2** block. Error bars indicate standard deviations (σ). The trend of widths correlating with DP of **P2** was preserved after CDSA.

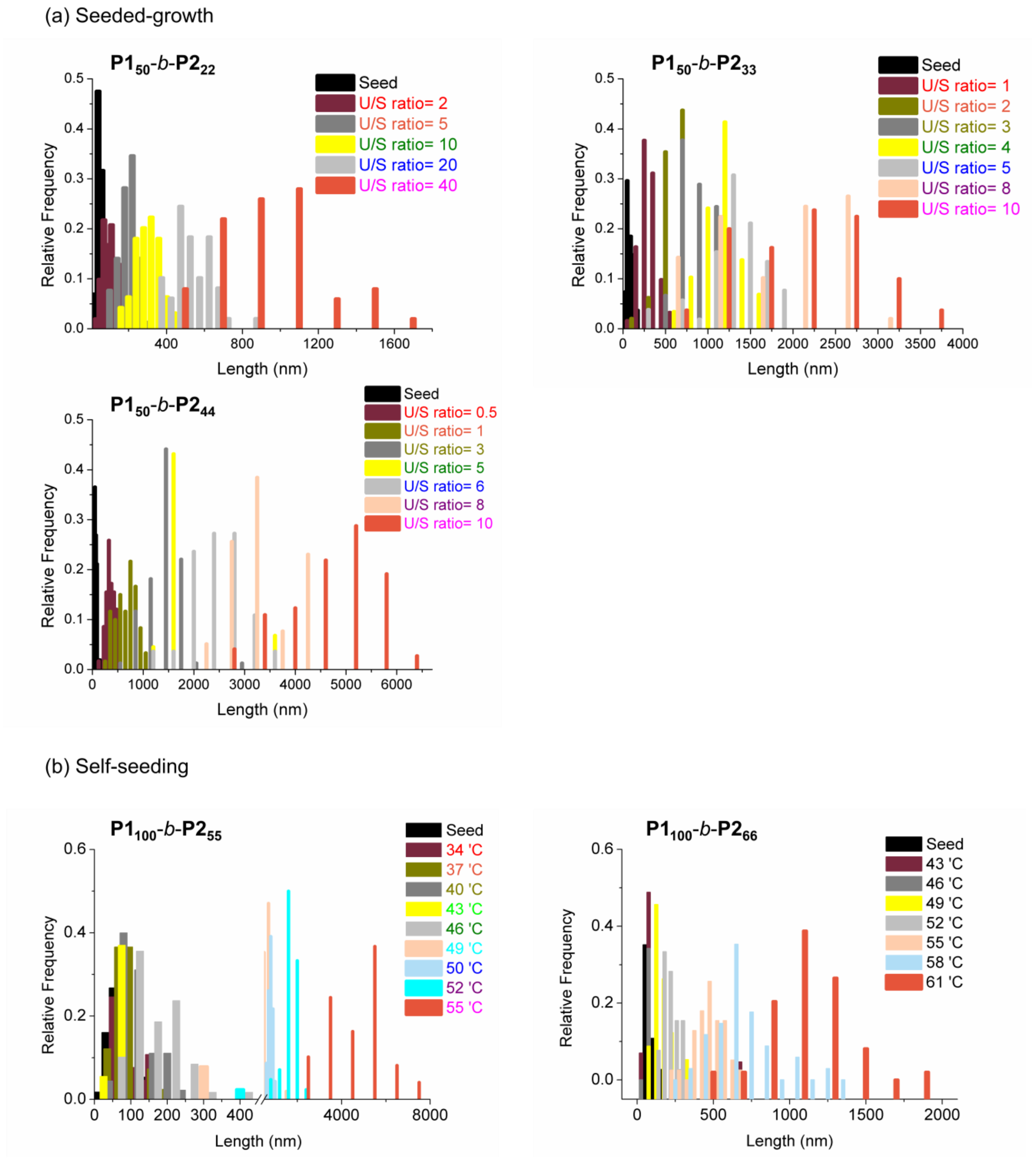

Fig. S41 Contour length histograms of all 1D nanofibers of (a) **P1$_{50}$**-*b*-**P2$_{22}$**, **P1$_{50}$**-*b*-**P2$_{33}$**, and **P1$_{50}$**-*b*-**P2$_{44}$** via the seeded growth mechanism, and (b) **P1$_{100}$**-*b*-**P2$_{55}$**, and **P1$_{100}$**-*b*-**P2$_{66}$** via the self-seeding mechanism with various U/S ratios. This figure showed the increase in lengths from seed length to the maximum length with histograms.

(a)

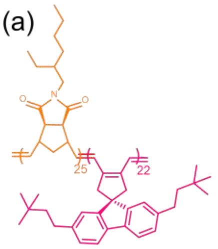

(b)

Entry	**[M3]**:**[M2]**:[cat]	Time for **M2**	Conv. (%)[a]	Yield (%)	M_n(KDa)[b]	$Đ$[b]
9	25:22:1	3 h	> 99%, > 99%	89.3	17.6	1.06

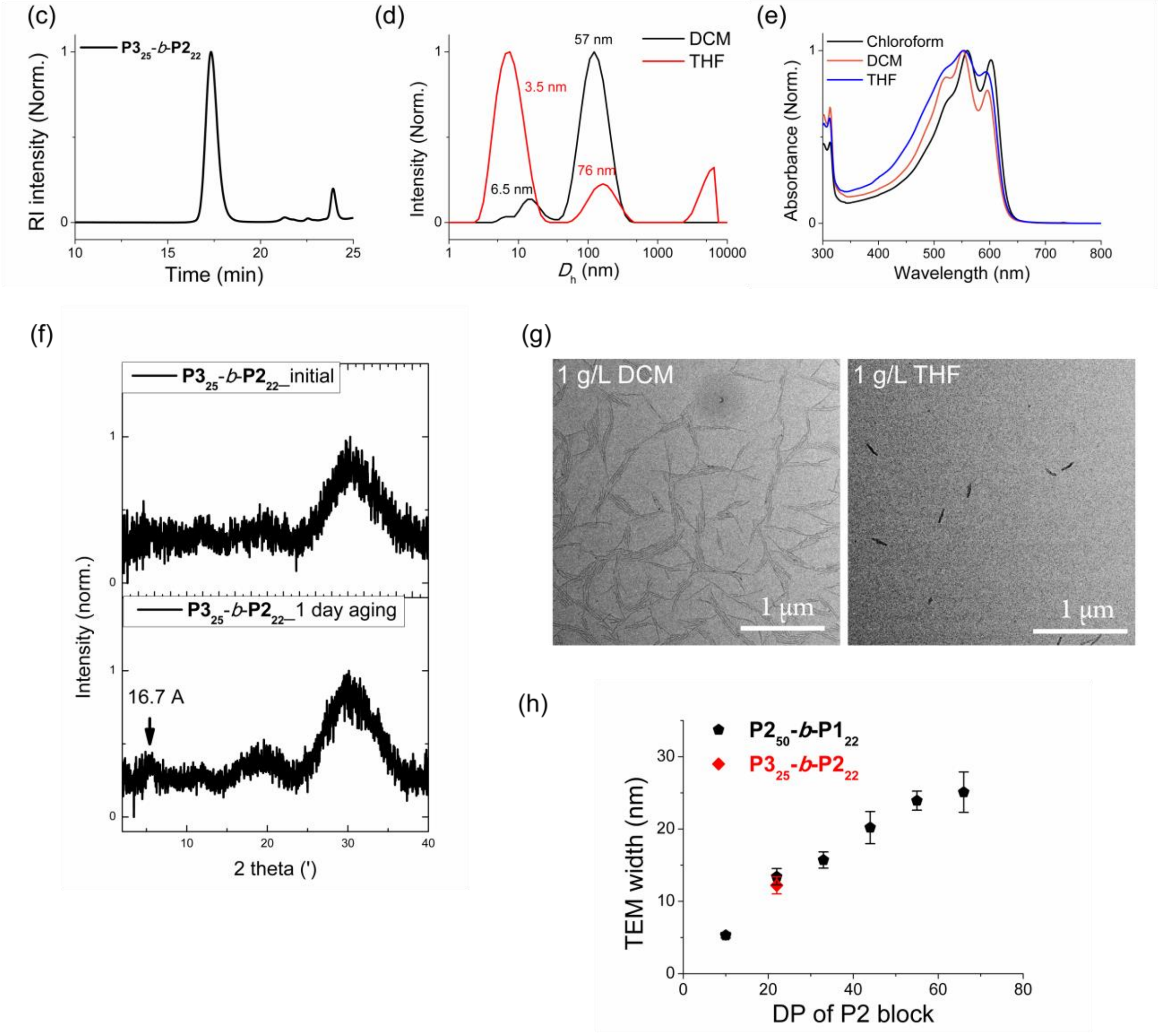

Fig. S42 (a) The chemical structure of **P3**$_{25}$-*b*-**P2**$_{22}$. (b) A table of the living cyclopolymerization of the **P3**$_{25}$-*b*-**P2**$_{22}$. (c) A normalized chloroform SEC trace, (d) DLS profiles, and (e) UV-Vis spectra of the BCP in initial 1 g/L chloroform, DCM, and THF. (f) FXRD spectra of the **P3**$_{25}$-*b*-**P2**$_{22}$ before and after aging by drop-casting of 10 g/L chloroform on SiO_2 surface. The peak intensity at 16.7 Å from **P2** block increased after 1 day aging due to assembly. More amorphous **P3** corona block made the higher intensity of broad peaks in the 20−40° range than that of **P1** corona block. (g) TEM images of the 1 g/L solutions. In chloroform, the polymer did not self-assembled at the initial, and neither D_h values nor nanoparticles were observed in DLS analysis or TEM imaging. (h) A plot of its average width (W_n) with those of **P1**$_{50}$-*b*-**P2**$_n$ vs. DP of **P2**. The W_n of **P3**$_{25}$-*b*-**P2**$_{22}$ (the red dot) was well-matched with the trend of widths of **P1**$_m$-*b*-**P2**$_n$ correlating with DP of **P2**. ([a]Calculated by ^{1}H NMR analysis. [b]Determinted by chloroform SEC using 0.205 as a *dn/dc* value).

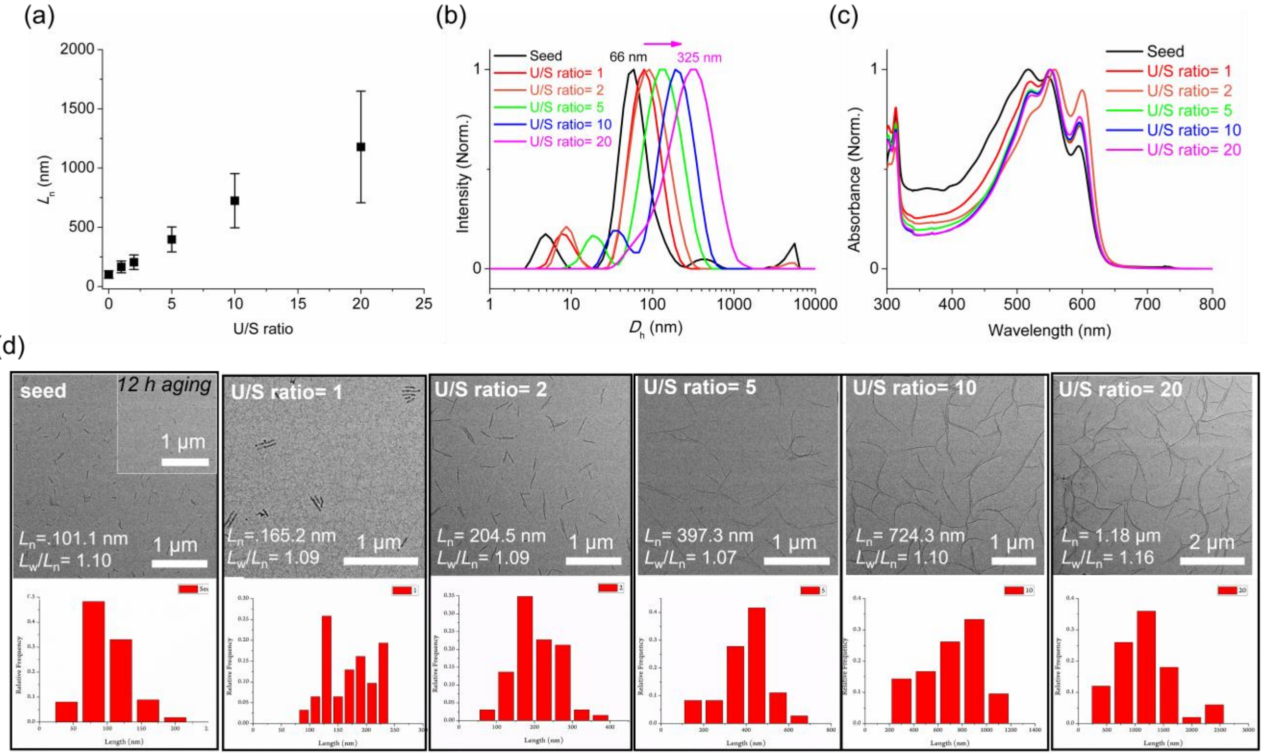

(e)

Initial DCM	W_n (nm)	W_w/W_n	σ/W_n	σ (nm)
	12.2	1.01	0.10	1.17
After CDSA	W_n (nm)	W_w/W_n	σ/W_n	σ (nm)
	13.1	1.02	0.14	1.90

Fig. S43 Living CDSA of $\mathbf{P3_{25}}$-*b*-$\mathbf{P2_{22}}$ in 0.1 g/L DCM. (a) A plot of the L_n vs. U/S ratio (addition of unimers in 10 g/L chloroform. (b) DLS profiles, (c) UV-vis absorbance spectra, (d) TEM images, and contour length histograms of the 1D nanofibers from $\mathbf{P3_{25}}$-*b*-$\mathbf{P2_{22}}$ prepared by living CDSA. (e) A table of average width (W_n) of the 1D nanofibers from TEM images.

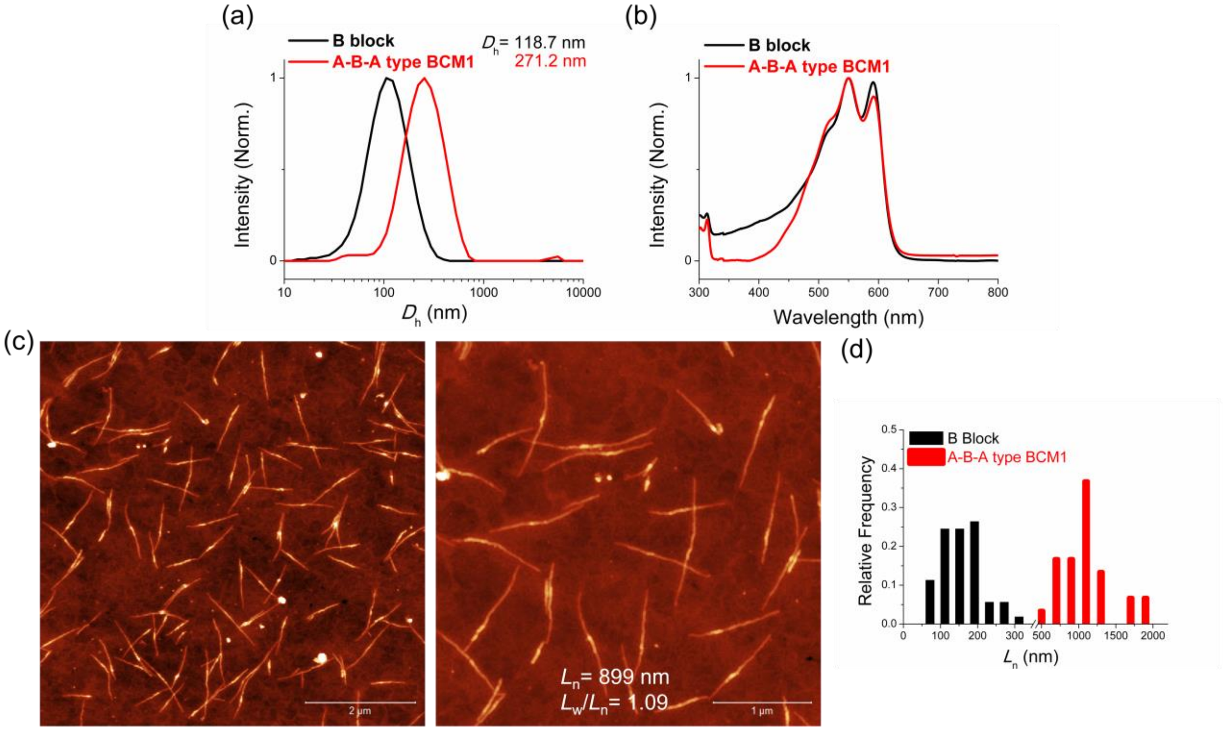

Fig. S44 (a) DLS profiles, (b) UV-vis spectra, and (c) AFM height images of ABA triblock comicelles 1 (**BCM1**) prepared by seeded growth of **P3**$_{25}$-*b*-**P2**$_{22}$ (**A**) from **P1**$_{50}$-*b*-**P2**$_{22}$ (**B**) seed micelles. First, the seed micelles (**B** block, with L_n of 169 nm (L_w/L_n = 1.10)) were prepared by seeded growth from the initial seeds using U/S ratio of 5 at 10 °C, then the second unimer with U/S ratio of 5 was added. (An optimal condition for **B** block changed to 0.02 g/L due to reducing the self-nucleation of the second unimer (**P3**$_{25}$-*b*-**P2**$_{22}$)). (d) Contour length histograms of the **B** block of **BCM1**, and the **BCM1**.

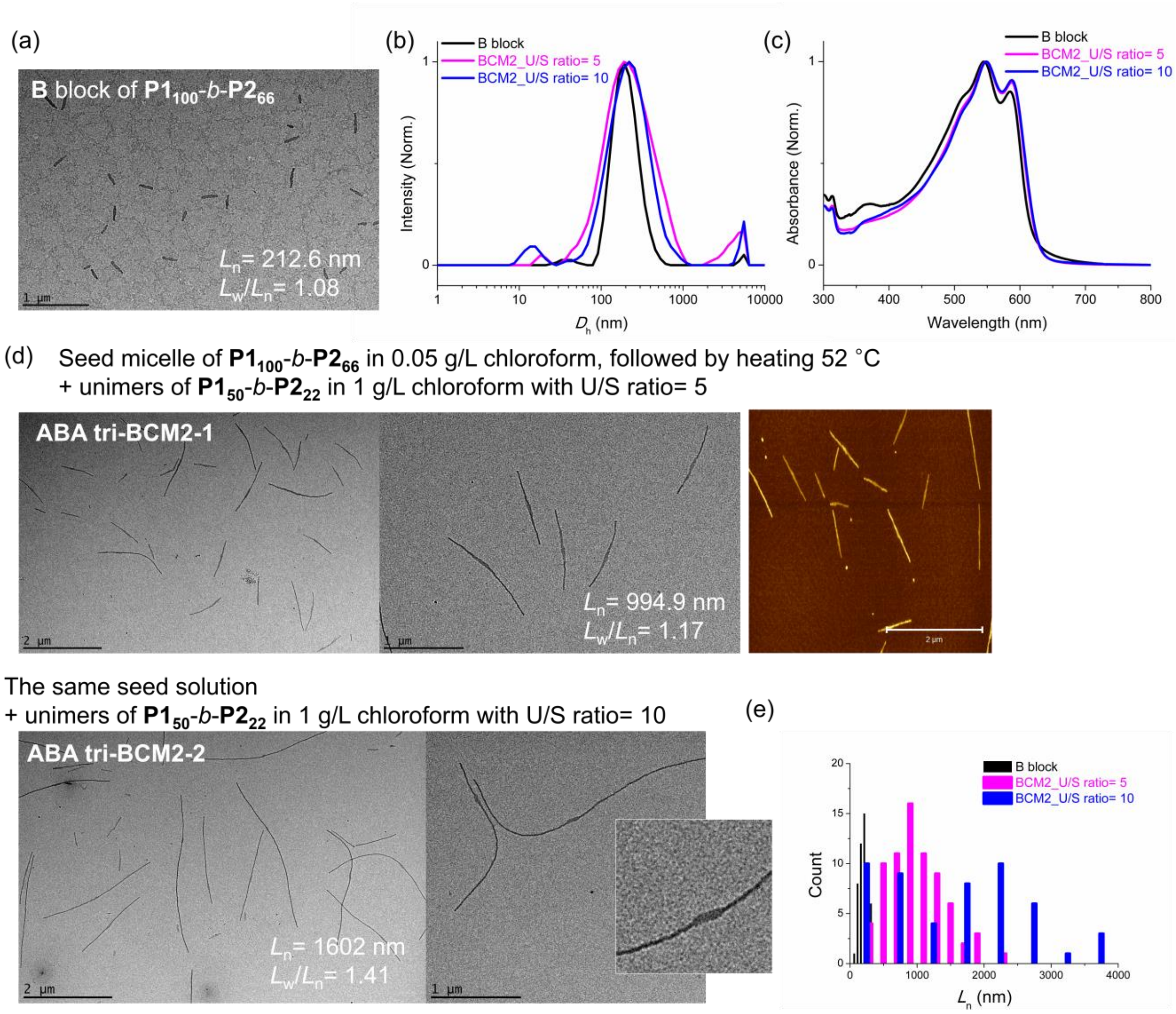

Fig. S45 Formation of another block comicelle (**BCM2**) from two different **P1**$_m$-*b*-**P2**$_n$ **BCPs**. (a) Seed micelles (**B**) prepared by self-seeding of **P1**$_{100}$-*b*-**P2**$_{66}$ at 52 °C in 0.05 g/L (seed statistical again, L_n = 213 nm (L_w/L_n = 1.08)). (b) DLS profiles, (c) UV-vis spectra, and (d) TEM and AFM height images of ABA triblock comicelles 2 (**BCM2**) prepared by seeded growth of **P1**$_{50}$-*b*-**P2**$_{22}$ (**A**) from the **B** block, showing two different widths dependent on DP of **P2** block. (e) Contour length histograms of the **B** block of **BCM2**, and the **BCM2s**.

☞ First order kinetic function: $$L(\mathrm{t}) = \frac{1}{N\frac{agg}{L}}\frac{[U]_0}{[S]_0}\left(1 - e^{-2k[S]_0 t}\right) + L_{seed}$$

☞ Stretched exponential function: $$L(\mathrm{t}) = A\left(1 - e^{-(k\prime t)^b}\right) + L_{seed}$$

Equation S1 These two functions were used to interpret the kinetic data for the seeded growth of **P1**$_{50}$-b-**P2**$_n$. [7]

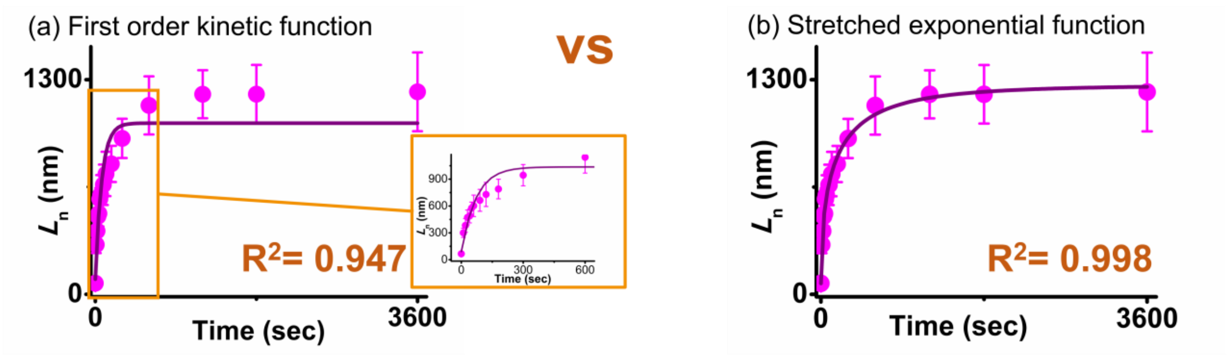

(c) Example data set

First order kinetic function

NL Fit (ExpDec1) (2019-11-21 13:50:01)

Notes

Description	NL Fit
User Name	°fë
Operation Time	2019-11-21 13:50:01
Model	ExpDec1
Equation	y = A1*exp(-x/t1) + y0
Report Status	New Analysis Report

Input Data

	Dep/Indep	Data	Range	Weight Type	Weight Data
x	Indep	[Book4]Sheet1!Time	[1*:18*]		
y	Dep	[Book4]Sheet1!Ln	[1*:18*]	Instrumental	[Book4]Sheet1!sigma

Parameters

		Value	Standard Error
Ln	y0	1036.58251	69.04626
	A1	-945.39459	71.47019
	t1	71.28903	12.4947

Iterations Performed = 9
Total Iterations in Session = 33
Fit converged - tolerance criterion satisfied.
Some input data points are missing.

Statistics

	Ln
Number of Points	15
Degrees of Freedom	12
Reduced Chi-Sqr	1.07333
Residual Sum of Squares	12.8799
Adj. R-Square	0.94743
Fit Status	Succeeded(100)

Fit Status Code :
100 : Fit converged

Stretched exponential function

NL Fit (NewFunction6 (User)) (2019-11-15 17:04:49)

Notes

Description	NL Fit
User Name	sangh
Operation Time	2019-11-15 17:04:49
Model	NewFunction6 (User)
Equation	y = L0 + A*(1-exp(-(k*x)^b))
Report Status	New Analysis Report

Input Data

	Dep/Indep	Data	Range	Weight Type	Weight Data
x	Indep	[Book4]Sheet1!Time	[1*:18*]		
y	Dep	[Book4]Sheet1!Ln	[1*:18*]	Instrumental	[Book4]Sheet1!sigma

Parameters

		Value	Standard Error
Ln	L0	66.5	0
	A	1795.74471	66.72987
	k	0.00583	7.91066E-4
	b	0.5867	0.02379

Iterations Performed = 13
Total Iterations in Session = 13
Fit converged - tolerance criterion satisfied.
Some parameter values were fixed.
Some input data points are missing.

Statistics

	Ln
Number of Points	15
Degrees of Freedom	12
Reduced Chi-Sqr	0.05537
Residual Sum of Squares	0.66444
Adj. R-Square	0.9969
Fit Status	Succeeded(100)

Fit Status Code :
100 : Fit converged

(d) Fitting results

First order kinetic function

U/S ratio= 5

Equation	y = A1*exp(-x/t1) + y0		
Adj. R-Squar	0.94743		
		Value	Standard Erro
Ln	y0	1036.5825	69.04626
Ln	A1	-945.39459	71.47019
Ln	t1	71.28903	12.4947

Stretched exponential function

U/S ratio= 2

Equation	y = L0 + A*(1-exp(-(k*x)^b))		
Adj. R-Square	0.99886		
		Value	Standard Error
Ln	L0	66.5	0
Ln	A	533.68625	9.44808
Ln	k	0.01112	8.5324E-4
Ln	b	0.47711	0.01453

U/S ratio= 3

Equation	y = L0 + A*(1-exp(-(k*x)^b))		
Adj. R-Square	0.99915		
		Value	Standard Error
Ln	L0	66.5	0
Ln	A	737.62545	9.12306
Ln	k	0.00986	5.15478E-4
Ln	b	0.53918	0.01421

U/S ratio= 5

Equation	y = L0 + A*(1-exp(-(k*x)^b))		
Adj. R-Square	0.99761		
		Value	Standard Error
Ln	L0	66.5	0
Ln	A	1199.55319	34.7774
Ln	k	0.00572	7.00593E-4
Ln	b	0.52843	0.02178

U/S ratio= 10

Equation	y = L0 + A*(1-exp(-(k*x)^b))		
Adj. R-Square	0.9969		
		Value	Standard Error
Ln	L0	66.5	0
Ln	A	1795.74471	66.72987
Ln	k	0.00583	7.91066E-4
Ln	b	0.5867	0.02379

Fig. S46 Fittings of the kinetic data were performed using Origin (OriginLab, Northampton, MA) software. Size of seed was held at the value from TEM images for all data sets. During a CDSA process of **P1$_{50}$**-*b*-**P2$_{33}$**, the micelle length at each time point was used to weight the fits (with an instrumental error). The kinetic values were obtained when the fits from **Equation S1** converged. We applied two different equations to the kinetic data (of U/S ratio = 5 here); (a) The first-order kinetic function and (b) the stretched exponential function in **Equation S1**. The stretched exponential function gave higher R^2 values. An average '*b*' value, which related to the "self-assembly of polymer chains" was obtained as 0.54. (c) Example data sets for the two fitting methods. Note: *k'* in the stretched exponential function model was reported in sec^{-1}. (d) The raw data of these fitting results which were reported in **Fig. 8b**.[7]

U/S ratio	2		3		5		10	
Time	nm							
sec	*L*(t)	σ	*L*(t)	σ	*L*(t)	σ	*L*(t)	σ
0	66.5	26.1	66.5	26.1	66.5	26.1	66.5	26.1
10	234.2	43.8	248.3	57.4	300.7	51.7	355.0	53.6
20	263.4	52.5	318.4	59.8	383.4	75.9	515.6	93.8
30	297.0	45.7	362.1	70.4	472.5	81.9	663.8	114.5
40	336.2	65.	394.6	80.8	488.9	77.7	703.6	119.2
50	349.2	57.4	434.5	59.7	576.7	67.1	747.1	186.7
60	365.8	49.1	475.3	72.8	602.1	119.6	867.0	174.7
90	403.3	56.3	501.8	90.3	663.2	122.3	959.8	159.8
120	430.1	83.3	553.5	85.5	729.0	134.5	1038.5	210.5
180	472.7	46.0	602.3	128.0	789.5	109.4	1150.9	258.8
300	509.9	51.8	694.8	128.3	944.2	118.8	1387.0	240.2
600	550.1	74.8	745.9	124.5	1144.0	176.2	1626.6	250.5
1200	585.0	130.3	786.7	141.3	1211.8	145.5	1728.0	378.5
1800	598.4	144.8	796.0	201.9	1212.0	177.4	1869.4	439.1
3600	593.8	124.3	805.6	119.1	1226.0	238.6	1957.6	449.9
14400	596.1	119.5	801.7	111.0	1239.5	326.9	2060.8	715.7
46800	592.2	116.6			1244.4	304.5	1988.6	700.7

Table S2 Data summary of kinetic studies on seeded growth of 1D nanofibers from $\mathbf{P1}_{50}$-*b*-$\mathbf{P2}_{33}$ in chloroform over 13 hours with various U/S ratios 2, 3, 5, and 10.

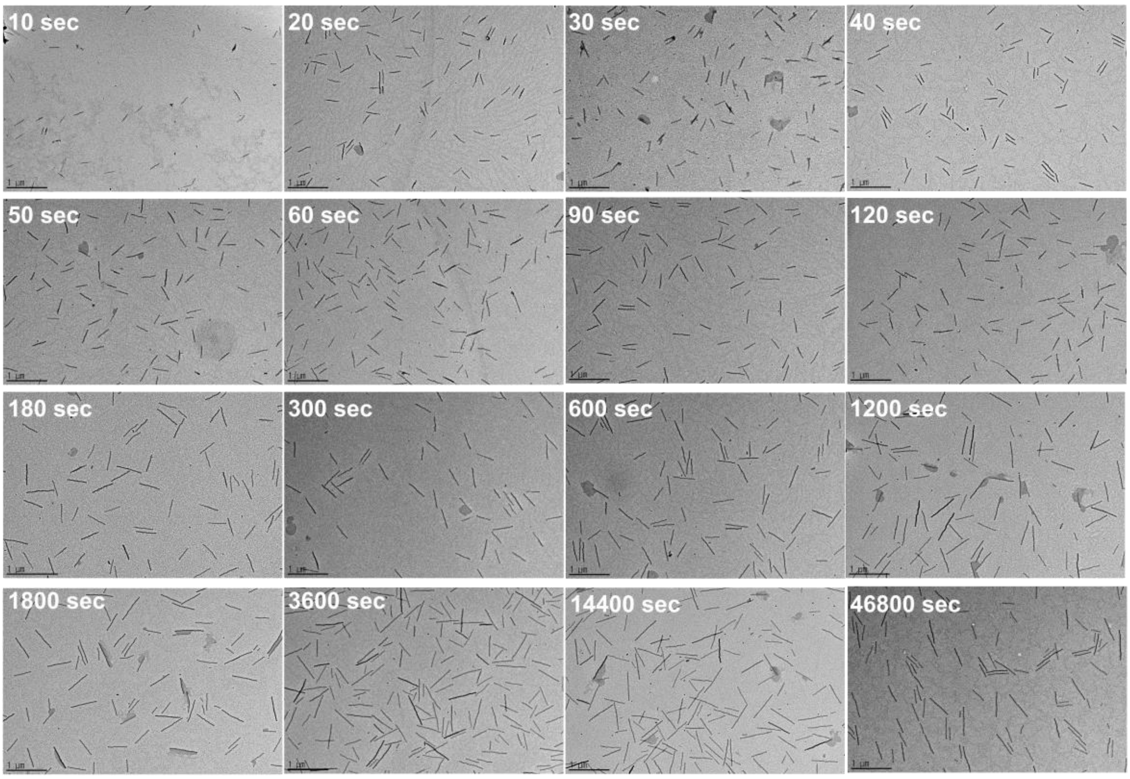

Fig. S47 TEM images of elongation of 1D nanofibers from **P1**$_{50}$-*b*-**P2**$_{33}$ in kinetic studies with a U/S ratio of 2. Upon the addition of the unimers (10 g/L chloroform), seeded growth of seed micelle (0.1 g/L chloroform) occurred at RT.

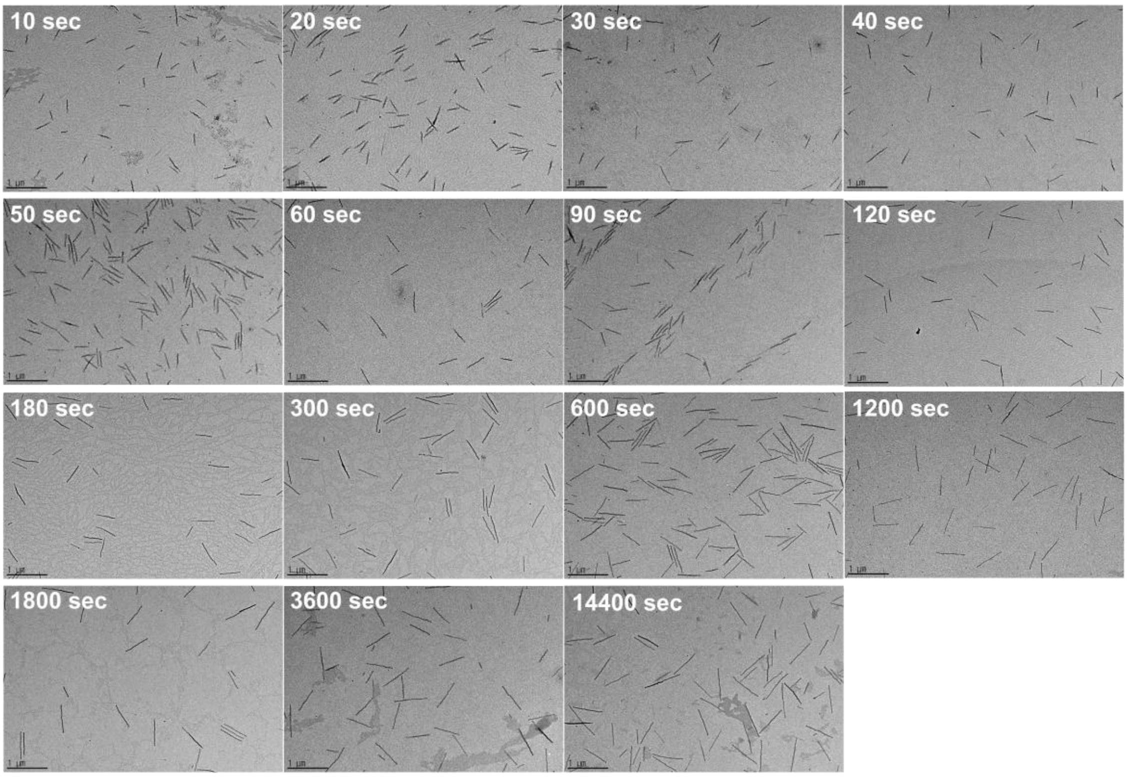

Fig. S48 TEM images of elongation of 1D nanofibers from **P1**$_{50}$-*b*-**P2**$_{33}$ in kinetic studies with a U/S ratio of 3. Upon the addition of the unimers (10 g/L chloroform), seeded growth of seed micelle (0.1 g/L chloroform) occurred at RT.

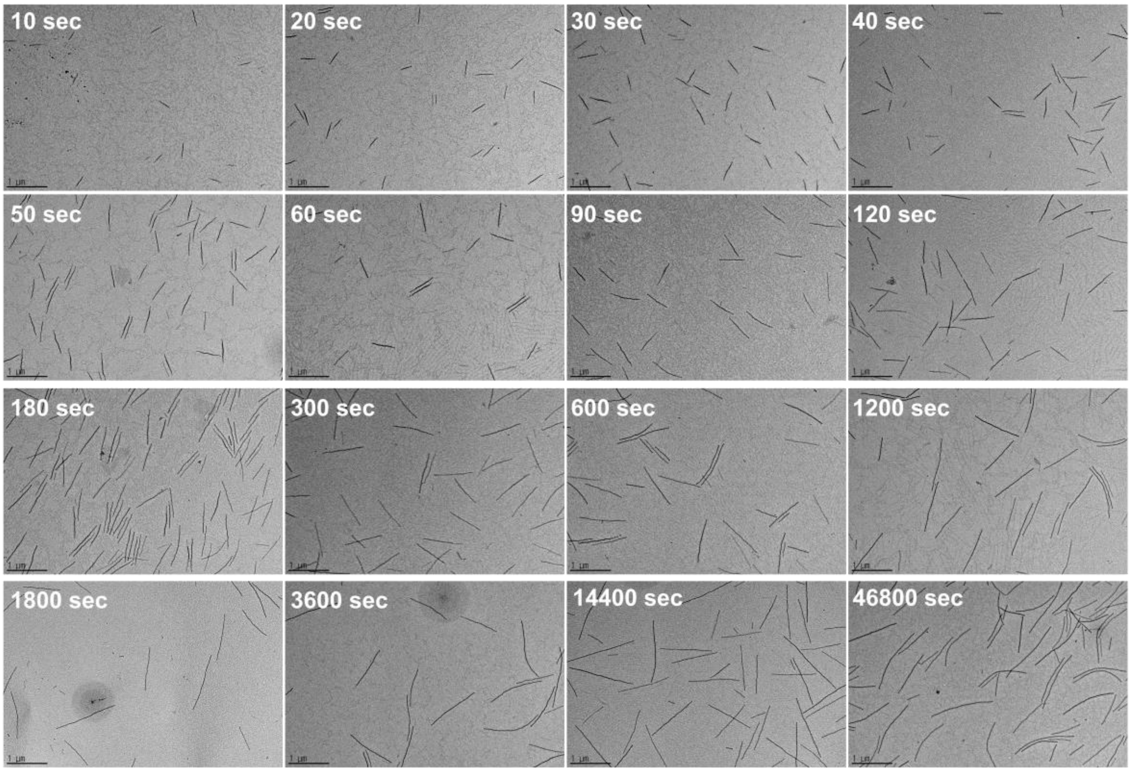

Fig. S49 TEM images of elongation of 1D nanofibers from **P1**$_{50}$-*b*-**P2**$_{33}$ in kinetic studies with a U/S ratio of 5. Upon the addition of the unimers (10 g/L chloroform), seeded growth of seed micelle (0.1 g/L chloroform) occurred at RT.

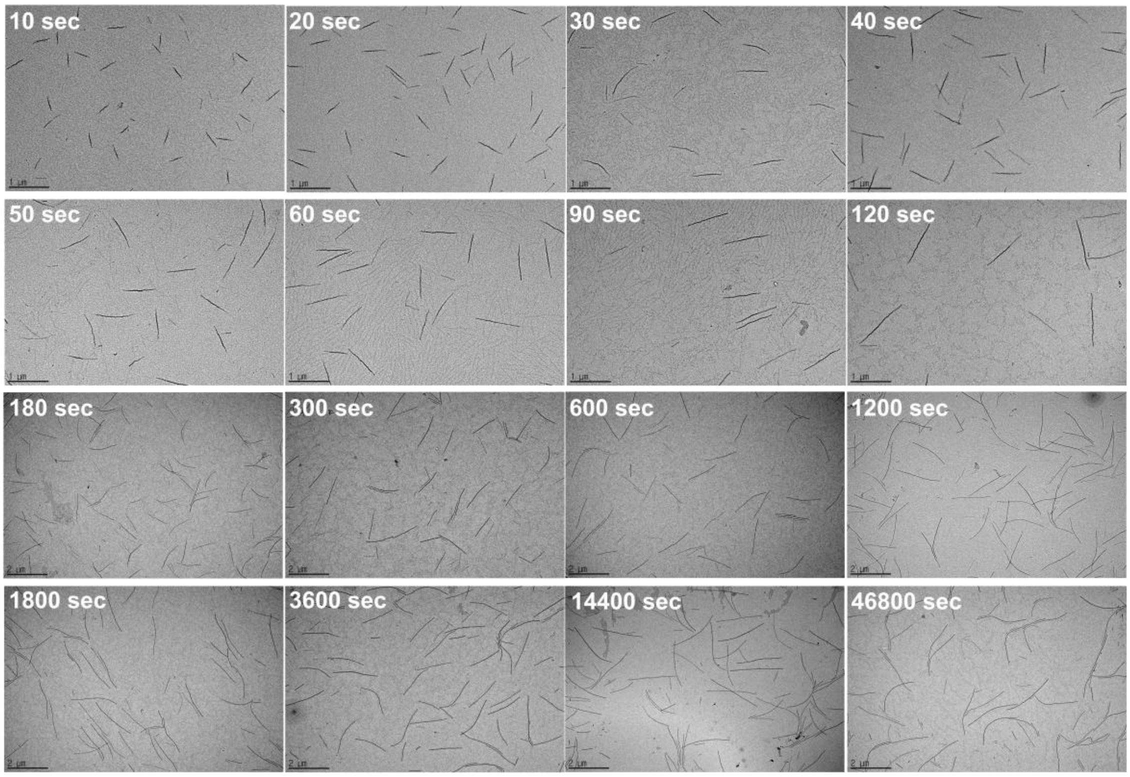

Fig. S50 TEM images of elongation of 1D nanofibers from **P1**$_{50}$-*b*-**P2**$_{33}$ in kinetic studies with a U/S ratio of 10. Upon the addition of the unimers (10 g/L chloroform), seeded growth of seed micelle (0.1 g/L chloroform) occurred at RT.

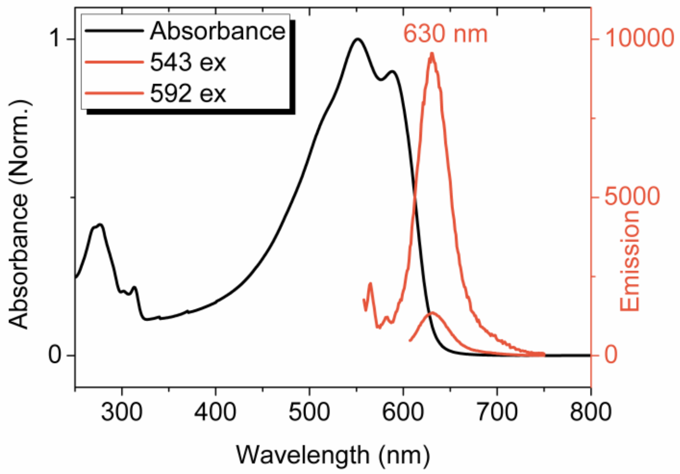

Fig. S51 The absorbance and emission spectra of **P1**$_{50}$-*b*-**P2**$_{22}$ in chloroform with excitation wavelength 543 nm and 592 nm.

4. Supporting Video Files

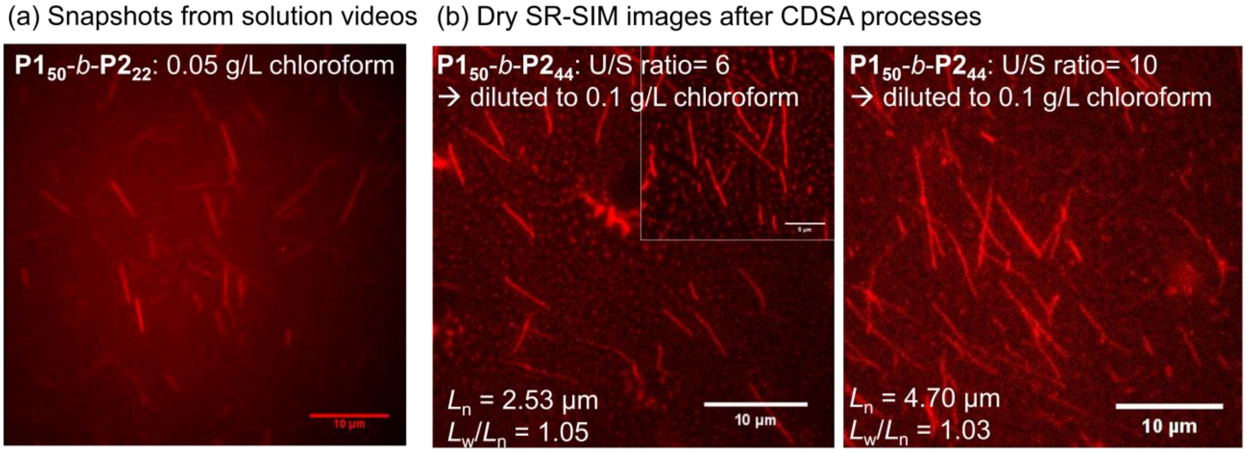

Fig. S52 (a) Fluorescent images captured from a **Video S3** showing the dynamics of 1D nanofibers from **P1**$_{50}$-*b*-**P2**$_{22}$ in 0.05 g/L chloroform. The real-time video indicated the free drift, rotation, and even collision of the 1D nanofibers, while maintaining their shapes (rigidity) and fluorescence. (b) Fluorescent images of dried-samples of 1D nanofibers from **P1**$_{50}$-*b*-**P2**$_{44}$ visualized by super-resolution structured illumination microscopy (SR-SIM). Dilution to observe the nanoparticles with proper density caused small defects.

Descriptions for the videos

Video S1: This movie described the real-time growth of 1D nanofibers from **P1**$_{50}$-*b*-**P2**$_{33}$. The optimized conditions for observing real-time elongation using a laser scanning confocal microscope (LSCM) were slightly different from those of living CDSA by using TEM imaging. Since it is difficult to focus on free-floating seeds, 0.01 g/L seed solution was scattered on the slide glass to obtained attached one to the surface. After adding unimer solution with U/S ratio of 30, slide glass was sealed with a cover-glass in short time, and a real-time movie could be obtained without solvent drying.
Even considering the 120 nm lateral resolution of the LSCM, after 2 minutes, the length of the attached seeds became longer, with a controlled growth rate. However, since real-time monitoring only reflects the growth on the surface, it was difficult to directly compare the results of real-time monitoring by LSCM with growth kinetics studies through TEM imaging.

Video S2: A gray-colored converted version of the **Video S1** showing more clear growth than **Video S2**.

Video S3: A movie of the dynamic movements of 1D nanofibers from **P1**$_{50}$-*b*-**P2**$_{22}$ in 0.05 g/L chloroform.

* The original video rate was 10 fps (10 frames per second). The number at the top left of each video was the number of frames, which allowed us to check the actual time.

5. NMR Spectra for New Block Copolymers

$P1_m$-*b*-$P2_n$ (^{1}H NMR, 500 MHz, $CDCl_3$)

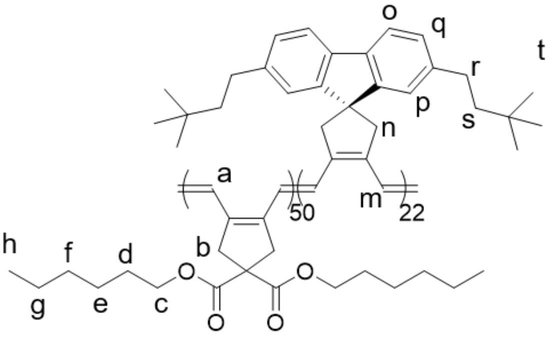

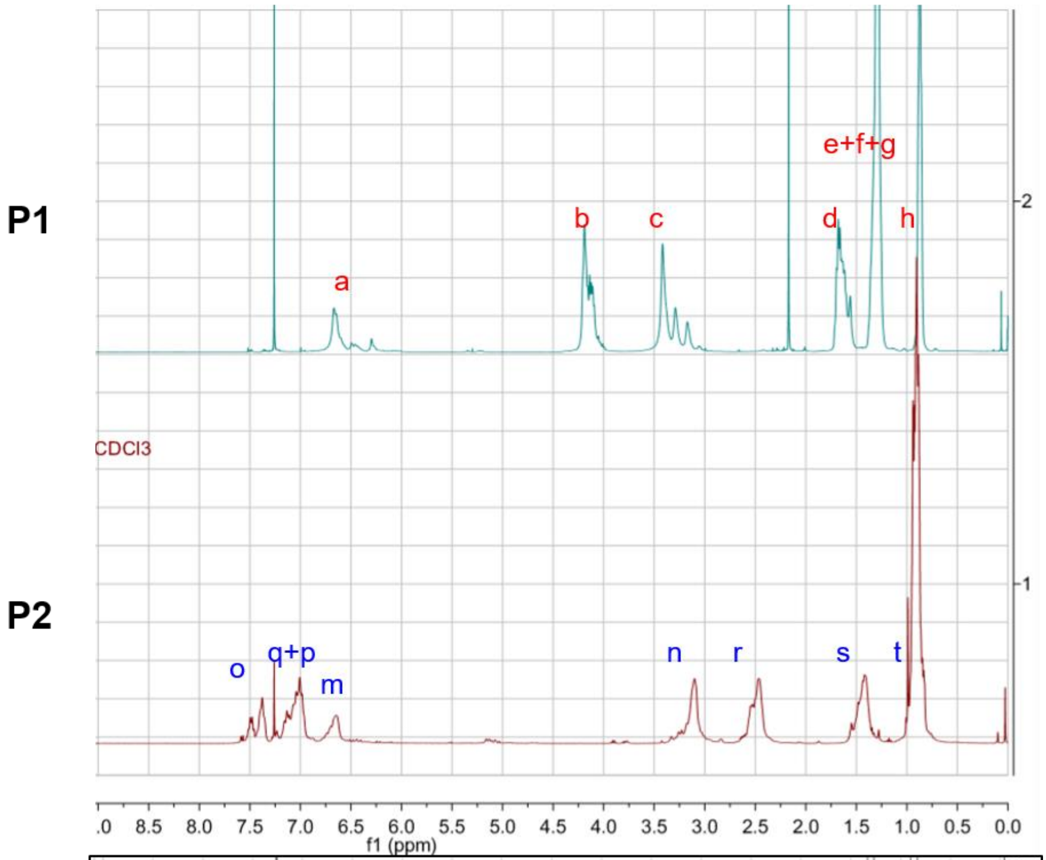

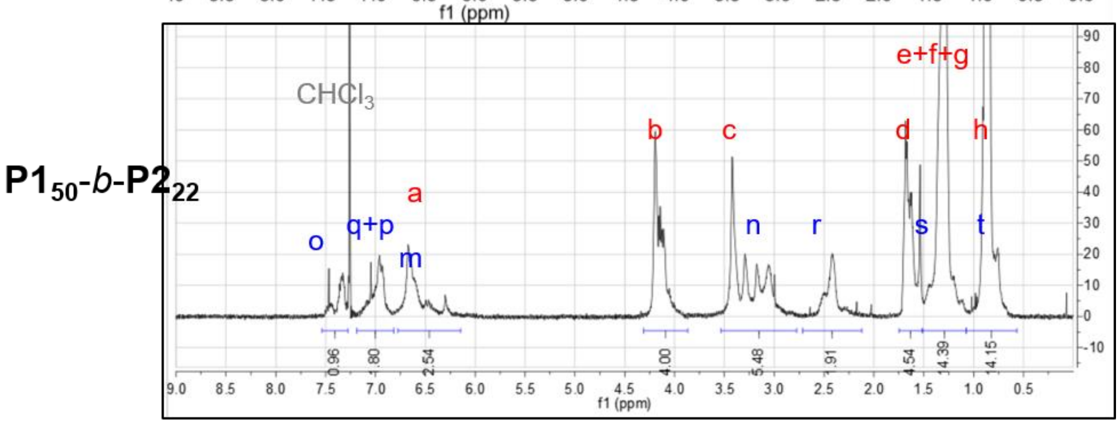

P3$_m$-*b*-P2$_n$ (^{1}H NMR, 500 MHz, $CDCl_3$)

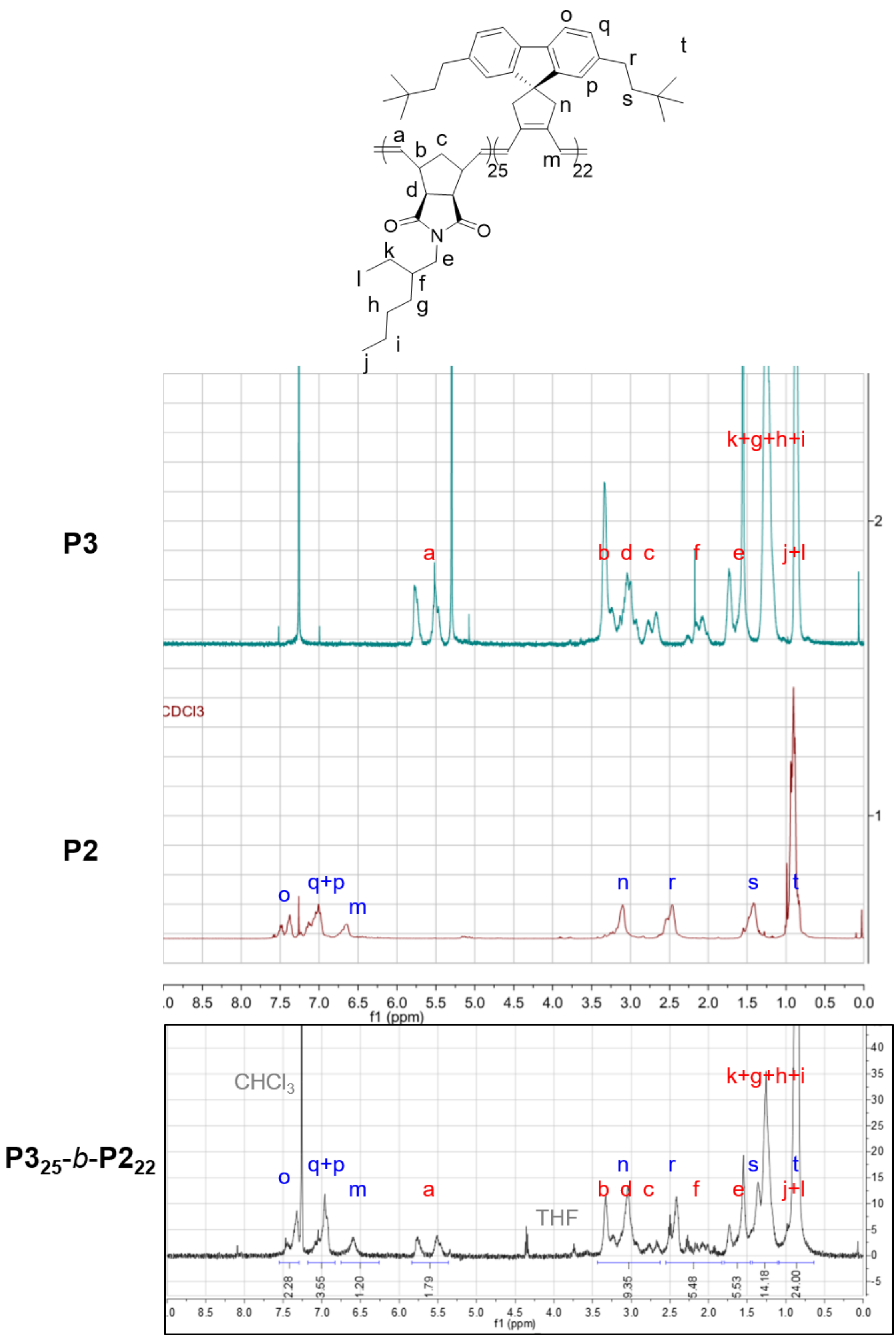

6. References

1. E.-H. Kang, I. S. Lee, T.-L. Choi, *J. Am. Chem. Soc.* **2011**, *133*, 11904–11907.

2. S. Yang, S. Shin, I. Choi, J. Lee, T.-L. Choi, *J. Am. Chem. Soc.* **2017**, *139*, 3082–3088.

3. F. Dutertre, K.-T. Bang, B. Loppinet, I. Choi, T.-L. Choi, G. Fytas, *Macromolecules* **2016**, *49*, 2731–2740.

4. A. C. Makan, T. Otte, H. Pasch, *Macromolecules* **2012**, *45*, 5247–5259.

5. S. Yang, S.-Y. Kang, T.-L. Choi, *J. Am. Chem. Soc.* **2019**, *141*, 19138–19143.

6. S. L. Potisek, D. A. Davis, N. R. Sottos, S. R. White, J. S. Moore, *J. Am. Chem. Soc.* **2007**, *129*, 13808–13809.

7. C. E. Boott, E. M. Leitao, D. W. Hayward, R. F. Laine, P. Mahou, G. Guerin, M. A. Winnik, R. M. Richardson, C. F. Kaminski, G. R. Whittell, I. Manners, *ACS Nano* **2018**, *12*, 8920–8933.
